# The Reactivity of Isomeric Nitrenium Lewis Acids with Phosphines, Carbenes, and Phosphide

**DOI:** 10.1002/chem.202004798

**Published:** 2021-01-14

**Authors:** Diya Zhu, Zheng‐Wang Qu, Jiliang Zhou, Douglas W. Stephan

**Affiliations:** ^1^ Department of Chemistry University of Toronto 80 St George St Toronto Ontario M5S3H6 Canada; ^2^ Mulliken Center for Theoretical Chemistry Institut für Physikalische und Theoretische Chemie Rheinische Friedrich-Wilhelms-Universität Bonn Beringstrasse 4 53115 Bonn Germany

**Keywords:** carbenes, N-Lewis bases, phosphides, phosphines, radicals

## Abstract

Alkylation of spiro[fluorene‐9,3’‐indazole] at N(1) and N(2) with *t*BuCl affords the nitrenium cations [C_6_H_4_N_2_(*t*Bu)C(C_12_H_8_)][BF_4_], **1** and **2**, respectively. Compound **1** converts to **2** over the temperature range 303–323 K with a free energy barrier of 28±5 kcal mol^−1^. Reaction of **1** with PMe_3_ afforded the *N*‐bound phosphine adduct [C_6_H_4_N(*t*Bu)N(PMe_3_)C(C_12_H_8_)]BF_4_] **3**. However, phosphines attack **2** at the *para*‐carbon atom of the aryl group with concurrent cleavage of N(2)−C(1) bond and proton migration to C(1) affording [(R_3_P)C_6_H_3_NN(*t*Bu)CH(C_12_H_8_)][BF_4_] (R=Me **4**, *n*Bu **5**). Analogous reactions of **1** and **2** with the carbene SIMes prompt attack at the *para*‐carbon with concurrent loss of H^.^ affording the radical cation salts [(SIMes)C_6_H_3_N(*t*Bu)NC(C_12_H_8_)^.^][BF_4_] **6** and [(SIMes)C_6_H_3_NN(*t*Bu)C(C_12_H_8_)^.^][BF_4_] **7**, whereas reaction of **2** with BAC gives the Lewis acid‐base adduct, [C_6_H_4_N(BAC)N(*t*Bu)C(C_12_H_8_)][BF_4_] **8**. Finally, reactions of **1** and **2** with KPPh_2_ result in electron transfer affording (PPh_2_)_2_ and the persistent radicals C_6_H_4_N(*t*Bu)NC(C_12_H_8_)^.^ and C_6_H_4_NN(*t*Bu)C(C_12_H_8_)^.^. The detailed reaction mechanisms are also explored by extensive DFT calculations.

## Graphical abstract

**Isomers of nitrenium cations** [C_6_H_4_N_2_(*t*Bu)C(C_12_H_8_)][BF_4_], **1** and **2**, react with carbenes and phosphines and phosphide. Small donors form *N*‐bound Lewis adducts. Larger donors attack at the aryl carbon *para*‐to N, where phosphines induces proton migration ring opening the N_2_C_3_ ring, while carbene prompt loss of H^.^ affording radical cation salts. Reactions of **1** and **2** with KPPh_2_ effects electron transfer affording (PPh_2_)_2_ and persistent radical products.

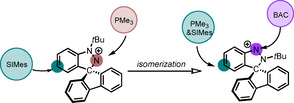

## Introduction

The classification of electron donors and acceptors as bases and acids, respectively delineated by Lewis in the 1920s is a concept that permeates much of chemistry.^[1]^ Indeed, this concept remains relevant today as one of the underpinnings of coordination and main group chemistry. Moreover, built on perturbations to Lewis’ fundamental notion, the concepts of „*umpolung“*[^2^, ^3^, ^4^, ^5^, ^6^, ^7^, ^8^, ^9^, ^10^, ^11^] and „*frustrated Lewis pairs (FLPs)*“[^12^, ^13^, ^14^, ^15^, ^16^] have led to new and unique approaches to synthetic and catalytic chemistry. The combination of these latter two concepts has unveiled new Lewis acids for FLP chemistry. For example, in 2013, we described the development of highly electrophilic phosphonium cations (EPCs) for the activation of C−F bonds.^[17]^ Moreover, those Lewis acids based on phosphorus, an element traditionally used in Lewis donors, proved useful in FLP hydrogenations, transfer hydrogenations and hydrosilylations.^[18]^

**Figure 1:**
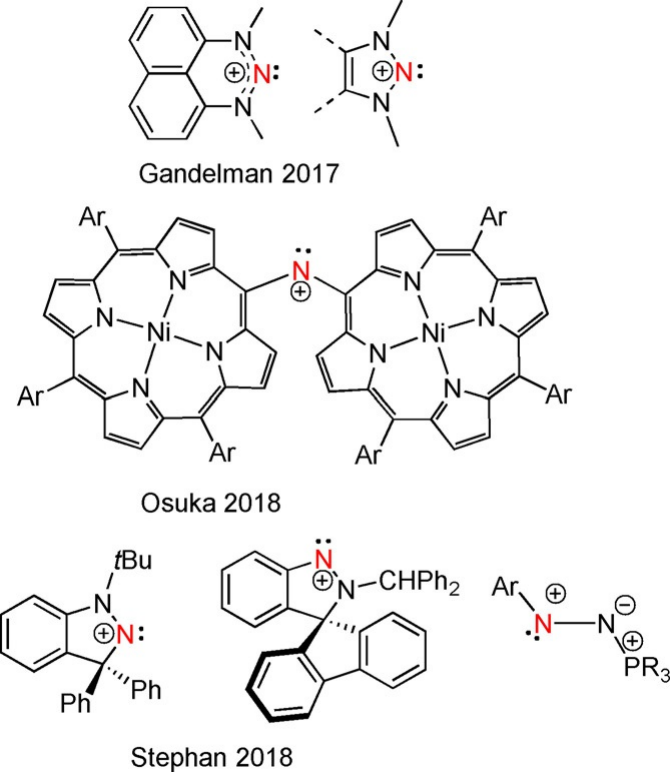
Recent examples of nitrenium cation‐based Lewis acids.

In a similar sense, nitrogen‐containing compounds usually feature an accessible lone pair at the N atom, and thus act as Lewis bases. Nonetheless, there are known examples of *N*‐centered electrophiles.[^19^, ^20^, ^21^, ^22^, ^23^] One class of such nitrogen‐centered electrophiles, nitrenium ions has drawn recent attention. In seminal work, Gandelman showed that a triazinium salt (*N*‐heterocyclic nitrenium (NHN)) can form classic Lewis acid/base adducts with phosphines (Figure 1). Gandelman and co‐workers have also expanded the range of triazolium salts, demonstrating access to a wide variety of N‐based Lewis acids.[^24^, ^25^, ^26^, ^27^] More recently, Mehta and Goicoechia^[28]^ as well as Gandelman^[29]^ have exploited the Lewis acidity of triazinium salts for Lewis acid catalysis and FLP Si−H activation.

In a concurrent effort, Osuka and co‐workers have described the synthesis of a stable diporphyrinylaminyl nitrenium cation.^[30]^ Moreover, they showed that this species was prepared by stepwise oxidation of the corresponding diporphyrinylamine through a stable aminyl radical.

In our own efforts, we developed a cyclic (amino)(alkyl)nitrenium (CAAN) cation that exhibited enhanced electrophilicity (Figure 1)^[31]^ forming Lewis adducts or participating in FLP reactivity when combined with phosphines or carbenes. This finding was also consistent with a computational study that found annulation to be an effective way to stabilize cyclic nitrenium ions.^[32]^ More recently, we also reported that alkylation of spiro[fluorene‐9,3’‐indazole] with Ph_2_CHCl affords a nitrenium cation that is indeed Lewis acidic at nitrogen.^[33]^ Interestingly, upon deprotonation of the benzylic carbon, the resulting zwitterion exhibits Lewis basicity at the nitrogen atom that was formerly acidic. In addition, we demonstrated that diazonium cations of the form [ArN_2_(PR_3_)]^+^ are N‐based Lewis acids and behave as one or two electron acceptors, yielding the corresponding radical and Lewis acid‐base adducts, respectively.^[34]^

In the present work, we further examine more steric‐hindered Lewis acidic nitrenium cations illustrating that alkylation of the N(1)‐position of fluorene‐9,3’‐indazole with a *tert*‐butyl group provides access to a nitrenium cation that thermally isomerizes to N(2)‐alkylated isomer. Moreover, these nitrenium cations exhibit diverse reactivity upon combination with donors such as phosphine, carbene, and phosphide. The observed products are shown to depend on both the steric demands and basicity of the donors.

## Results and Discussion

**Scheme 1:**
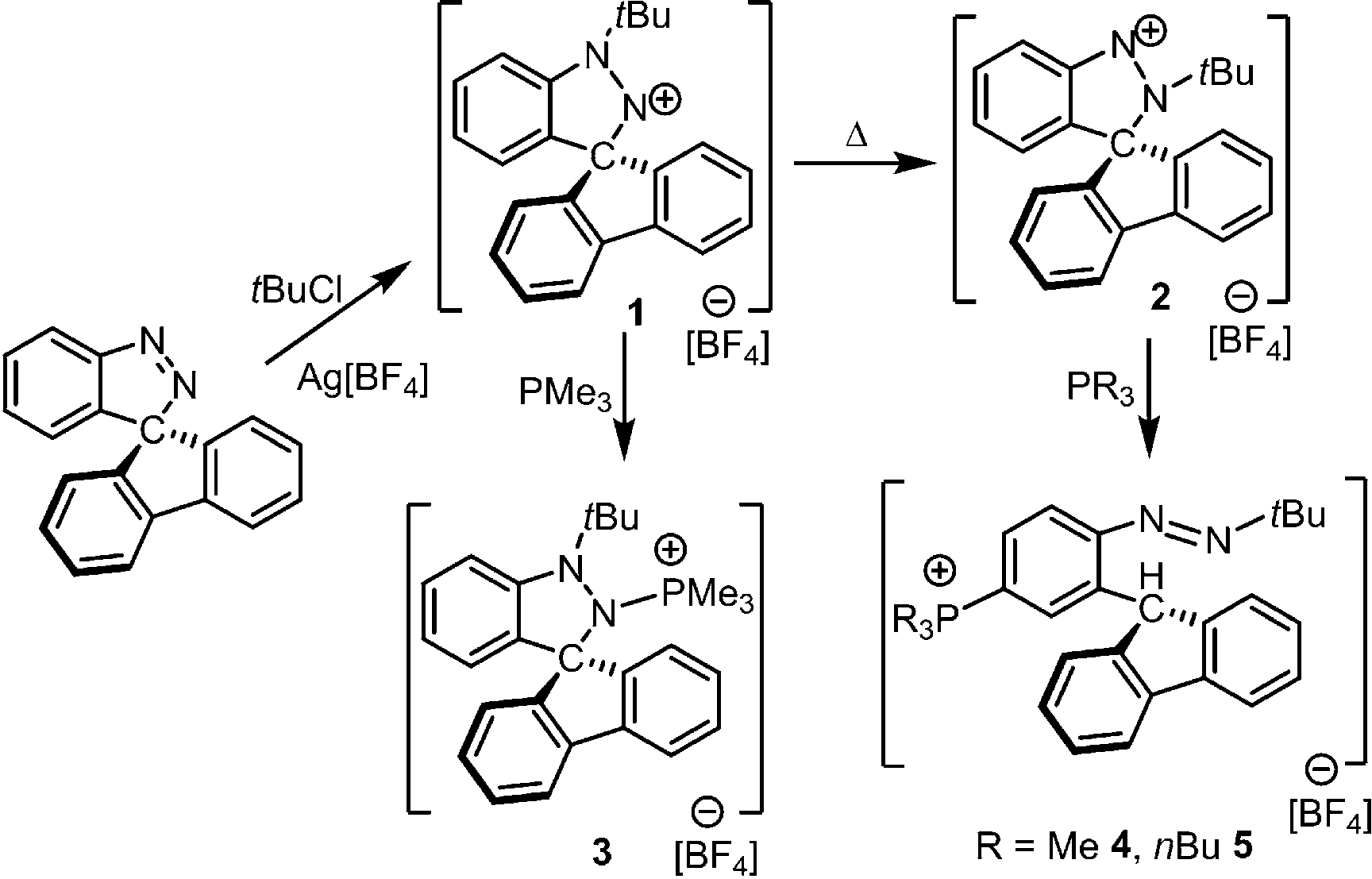
Synthesis of **1**–**5**.

Reaction of the spiro[fluorene‐9,3’‐indazole] with *t*BuCl and Ag[BF_4_] in CH_2_Cl_2_ at room temperature generates two products, **1** and **2** (Scheme 1). Under these conditions, **2** is the dominant product containing about a 1: 2.6 ratio of **1**: **2**. After work‐up, these salts were separated by recrystallization from a saturated CH_2_Cl_2_/pentane solution at −35 °C with the bright orange product **1** precipitating first in 24 % yield and the sandy orange product **2** requiring higher concentrations of pentane was isolated in 65 % yield. These products are readily distinguished by ^1^H NMR spectra and they show distinct resonances at 1.54 and 2.61 ppm attributable to the *t*Bu groups of **1** and **2**, respectively.

**Figure 2:**
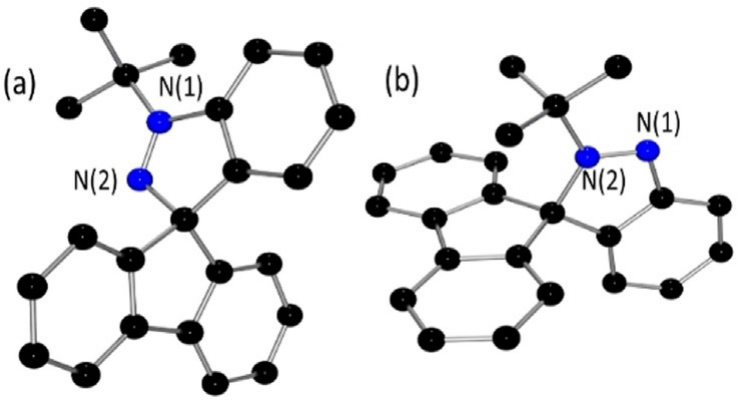
POV‐ray depictions of cations (a) **1** and (b) **2**; hydrogen atoms and BF_4_ anions are omitted for clarity. C: black; N: blue. Selected bond lengths: (a) N(1)−N(2) 1.263(5), (b) N(1)−N(2) 1.265(3) Å.

Crystallographic studies (Figure 2) confirmed the product salts as [C_6_H_4_N(*t*Bu)NC(C_12_H_8_)][BF_4_] **1** and [C_6_H_4_NN(*t*Bu)C(C_12_H_8_)][BF_4_] **2**, which are isomers in which alkylation occurred at the N(1) and N(2) positions, respectively. The metric parameters in the two isomers were similar. For example, the N(1)−N(2) bond lengths in **1** and **2** were found to be 1.263(5) and 1.265(3) Å, respectively, slightly longer than typical N=N double bonds. However, the difference between the torsion angles of N(1)‐N(2)‐C(1)‐C(19) in **1** (124.4(3)°) and N(1)‐N(2)‐C(1)‐C(15) in **2** (128.2(2)°) is consistent with variations in the steric conflict between the bulky fluorene and the *t*Bu‐substituent. In contrast to previously reported CAANs,[^31^, ^33^] compound **1** was observed to slowly convert to **2** in solution at ambient temperature. The initial rates observed for this isomerization was carefully monitored by ^1^H NMR spectroscopy. The rate of formation of **2** showed a first order dependence on the concentration of **1** (see Figure S32 in the Supporting Information). While the solubility of **1** limited the temperature range accessible, an Eyring plot analysis was performed with kinetic data collected over the temperature range 303–323 K, with the free energy Δ*G* (activation enthalpy Δ*H*) barrier of the isomerization determined as 28±5 (34±3) kcal mol^−1^.

**Scheme 2:**
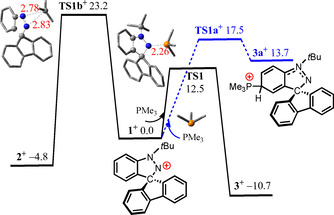
DFT‐computed free energy paths (in kcal mol^−1^, at 298 K and 1 mol L^−1^) of the reaction of cation **1^+^** with PMe_3_.

To further probe the mechanistic implications of these kinetic data, DFT calculations at the PW6B95‐D3/def2‐QZVP+OSMO‐RS//TPSS‐D3/def2‐TZVP+COSMO level of theory in CHCl_3_ solution[^35^, ^36^, ^37^, ^38^, ^39^, ^40^, ^41^, ^42^, ^43^, ^44^, ^45^, ^46^, ^47^, ^48^, ^49^] was undertaken (see the Supporting Information). Both salts **1** and **2** should exist as separated ion pairs **1^+^** and **2^+^**, respectively (see Computational details in the Supporting Information). The conversion from cation **1^+^** to **2^+^** is −4.8 kcal mol^−1^ exergonic over a free energy (activation) barrier of 23.2 (32.6) kcal mol^−1^ via a 1,2 *tert*‐butyl‐migration (via transition structure **TS1 b^+^**, Scheme 2), in excellent agreement with the kinetic experimental data.

**Figure 3:**
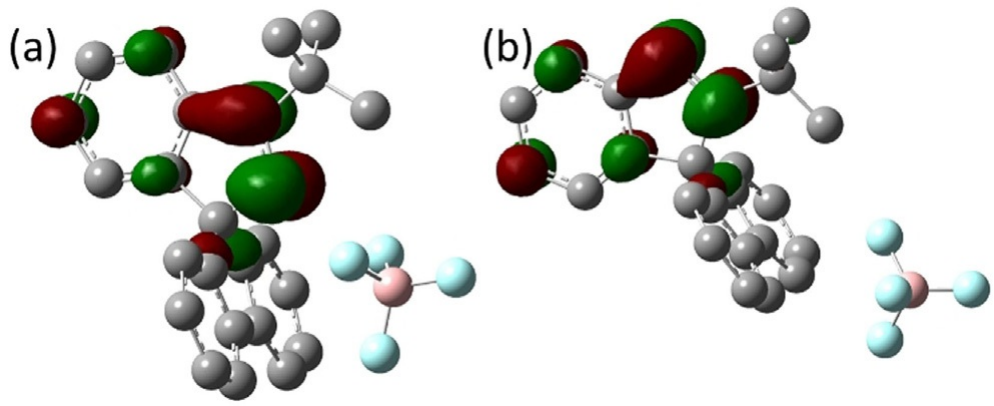
Depictions of the LUMOs of (a) **1**, (b) **2** in CHCl_3_ solution; computed at the TPSS‐D3/def2‐TZVP+COSMO level.

The nature of the LUMOs in the isomeric cations **1** and **2** was probed computationally (Figure 3). In both cases, the LUMOs consist of the π* orbital mainly located on the conjugated N=N bond and its adjacent aryl ring. Given that these contributions to the Lewis acidity of **1** and **2** have distinctly different degrees of steric congestion at nitrogen centers, divergent reactivity toward nucleophiles can be expected.

**Figure 4:**
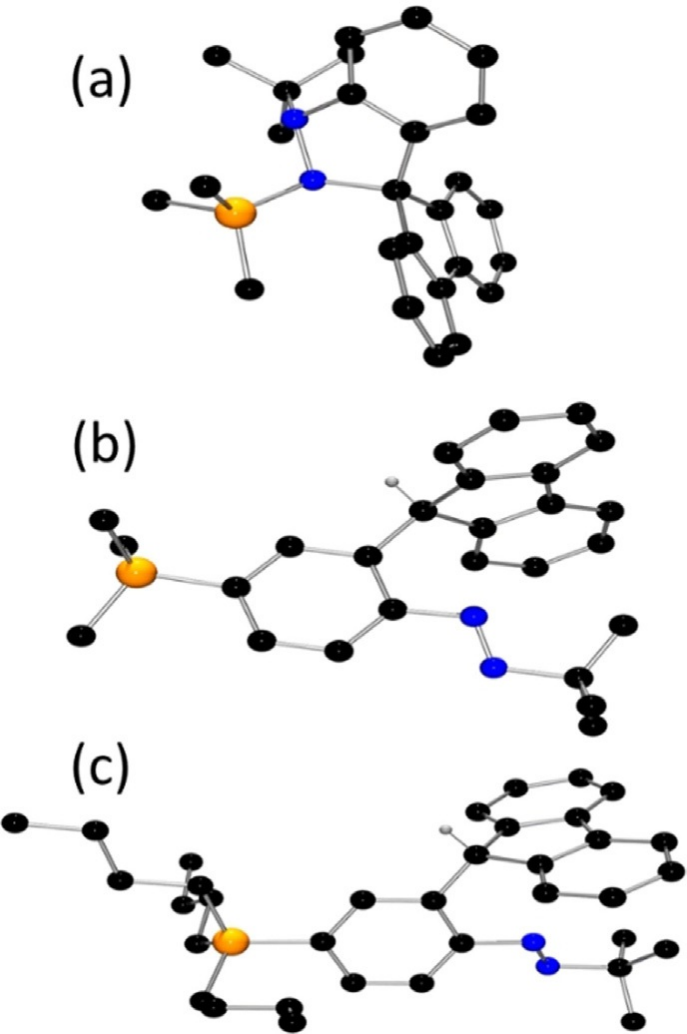
POV‐ray depictions of cations (a) **3**, (b) **4**, and (c) **5**; hydrogen atoms and BF_4_ anions are omitted for clarity. C: black; N: blue; P: orange. Selected bond lengths: **3**: N(1)−N(2) 1.470(4), N(1)−P(1) 1.679(3) Å; **5**: N(1)−N(2) 1.228(5), C(5)−P(1) 1.783(5) Å.

To begin to probe this possibility, reactions of these nitrenium cations with phosphine and carbene donors were probed. Initially, **1** was exposed to an excess amount of PMe_3_. Upon addition, the intensely orange solution of **1** faded to colorless immediately. A white solid **3** was isolated in 95 % yield. The ^31^P NMR spectrum of **3** showed a signal at 64.6 ppm, which is similar to that seen for previously reported CAAN‐phosphine adducts.[^31^, ^33^] Indeed the coordination of phosphine to nitrogen with the pyramidalization of nitrogen atom in [C_6_H_4_N(*t*Bu)N(PMe_3_)C(C_12_H_8_)][BF_4_] **3** was confirmed by an X‐ray diffraction study (Scheme 1, Figure 4). The resulting N−P distance was found to be 1.679(3) Å which is similar to that reported for [C_6_H_4_(*t*Bu)NN(PMe_3_)C(C_6_H_5_)_2_]^+^ (1.693(3) Å),^[31]^ [(C_12_H_8_)(Ph_2_CH)NN(C_6_H_4_)(PMe_3_)]^+^ (1.686(2) Å)^[33]^ and typical of N−P single bonds.^[51]^

The analogous treatment of **2** with PMe_3_ afforded a tan solid **4** in 92 % yield after workup (Scheme 1). The ^31^P{^1^H} NMR spectrum of **4** revealed a sharp singlet at 22.0 ppm while the ^1^H NMR spectrum showed a new resonance appearing at 6.21 ppm. Further, 2D HSQC NMR spectroscopic data affirmed the correlation of this proton signal with the resonances at 47.2 ppm in the ^13^C{^1^H} NMR spectrum arising from a sp^3^ carbon atom. Repeated efforts to characterize **4** by X‐ray crystallographic methods gave consistently poor quality crystals, although diffraction data confirmed the connectivity in which phosphine is bound to the carbon atom *para‐* to N with concurrent cleavage of N(2)−C bond and proton migration affording [(Me_3_P)C_6_H_3_NN(*t*Bu)CH(C_12_H_8_)][BF_4_] **4** (Figure 4).

In a similar fashion, addition of *n*Bu_3_P to **2** resulted phosphonium diazo salt **5** in 95 % yield (Scheme 1). The crystallographic study confirmed the formulation as [(*n*Bu_3_P)C_6_H_3_NN(*t*Bu)CH(C_12_H_8_)][BF_4_]. With two molecules in the asymmetric unit, the average N−N bond length was determined to be 1.232(5) Å, while the P‐C distances were found to be 1.783(5) and 1.802(5) Å. It is noteworthy that the nucleophilic attack at the carbon *para‐* to the Lewis acidic N(1) in **2** is reminiscent of the reaction of bulky phosphines with B(C_6_F_5_)_3_ in which the donor attack *para‐* to the Lewis acidic boron affording the phosphonium‐borate zwitterions, (R_3_P)C_6_F_4_B(C_6_F_5_)_2_F.[^52^, ^53^]

To garner further insight, the free energy paths of the reactions of both salts **1** and **2** with phosphine PMe_3_ in CHCl_3_ solution were computed at the PW6B95‐D3/def2‐QZVP+COSMO‐RS//TPSS‐D3/def2‐TZVP+COSMO level of theory. The reaction of cation **1^+^** with PMe_3_ was computed to proceed via the direct P⋅⋅⋅N nucleophilic attack at the N(2)‐site to give the stable Lewis acid‐base adduct **3**, which is −10.7 kcal mol^−1^ exergonic over a low free energy barrier of 12.5 kcal mol^−1^ (via **TS1**, Scheme 2). The alternative P⋅⋅⋅C nucleophilic attack at the aryl *para*‐carbon is 13.7 kcal mol^−1^ endergonic over a barrier that is 5.0 kcal mol^−1^ higher (via **TS1 a^+^**) to give the adduct **3 a**. Further deprotonation of **3 a** with PMe_3_ is unlikely due to an additional barrier of 21.0 kcal mol^−1^ (see the Supporting Information). It is noteworthy that the formation of **3** is also kinetically 10.7 kcal mol^−1^ favored over the isomerization of **1** to **2** (1,2‐shift of *t*Bu via **TS1 b^+^**).

**Scheme 3:**
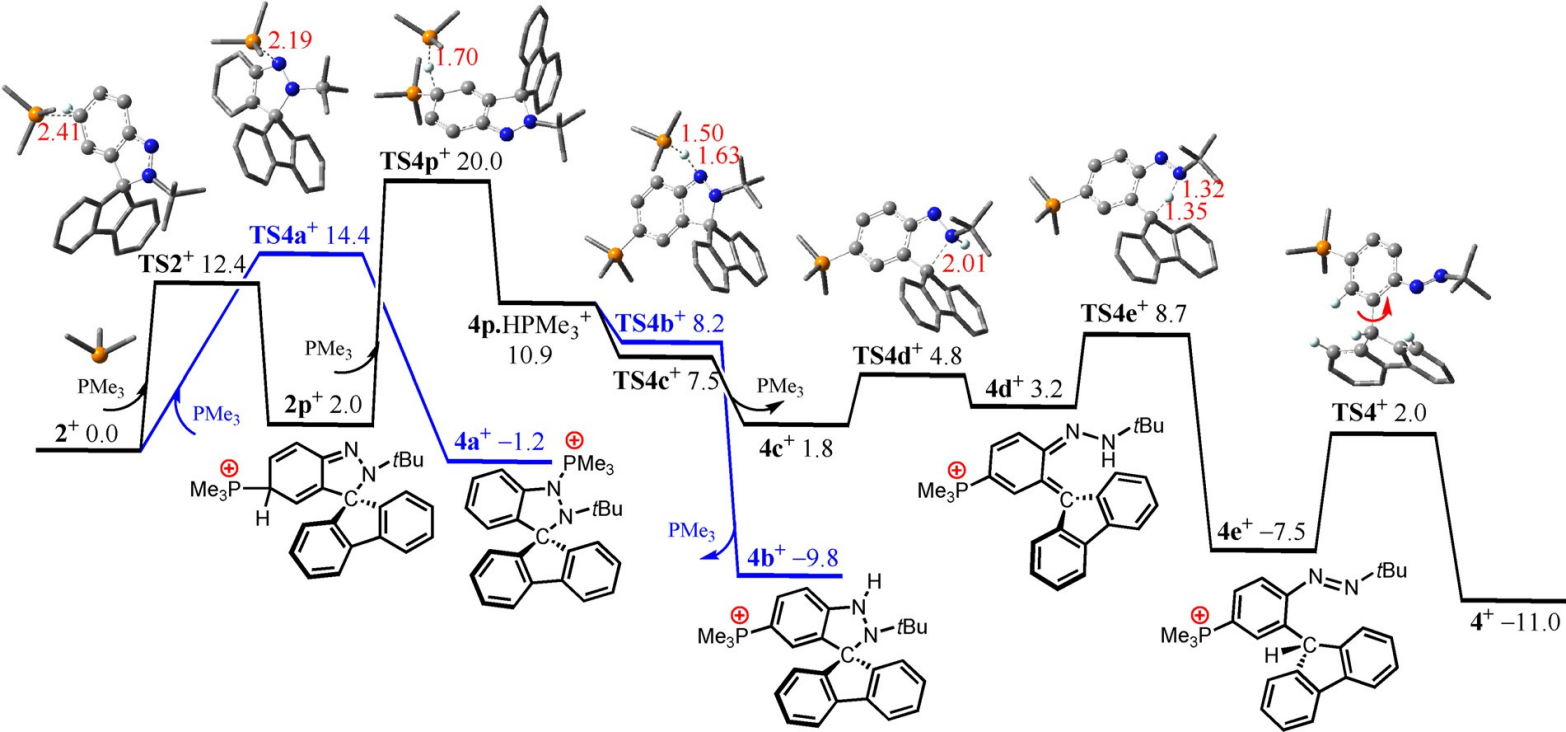
DFT‐computed free energy paths (in kcal mol^−1^, at 298 K and 1 mol L^−1^) for the reaction of cation **2^+^** with PMe_3_.

Computations for the corresponding reaction of the cation **2^+^** with PMe_3_ show that nucleophilic attack at the N(1)‐site is only −1.2 kcal mol^−1^ exergonic over a low barrier of 14.4 kcal mol^−1^ (via **TS4 a^+^**) and thus reversible at ambient temperature (Scheme 3). On the other hand, the nucleophilic attack by PMe_3_ at the aryl *para*‐ to N is 2.0 kcal mol^−1^ endergonic over barrier that is 2.0 kcal mol^−1^ lower in energy (via **TS2^+^**) yielding the transient adduct **2 p^+^**. Deprotonation by additional PMe_3_ is 8.9 kcal mol^−1^ endergonic over a moderate barrier of 18.0 kcal mol^−1^ (via **TS4 p^+^**) However, the resulting transient phosphonium‐complex **4 p**, can mediate protonation of at the basic *N*‐sites with an overall barrier of 21.2 kcal mol^−1^ due to reversibly formed adduct **4 a^+^**. Protonation at the N(1)‐site is barrierless but and −20.7 kcal mol^−1^ exergonic giving the cation **4 b^+^** and free PMe_3_. Alternatively, protonation at the N(2)‐site (via **TS4 c^+^**) is also barrierless, prompting proton transfer to carbon, C−N bond cleavage rotation of 9‐fluorenyl group to give the cation of **4**.

**Scheme 4:**
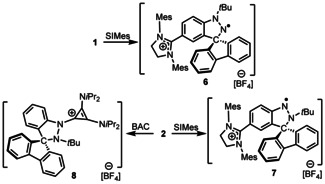
Synthetic routes to **6**–**8**.

**Figure 5:**
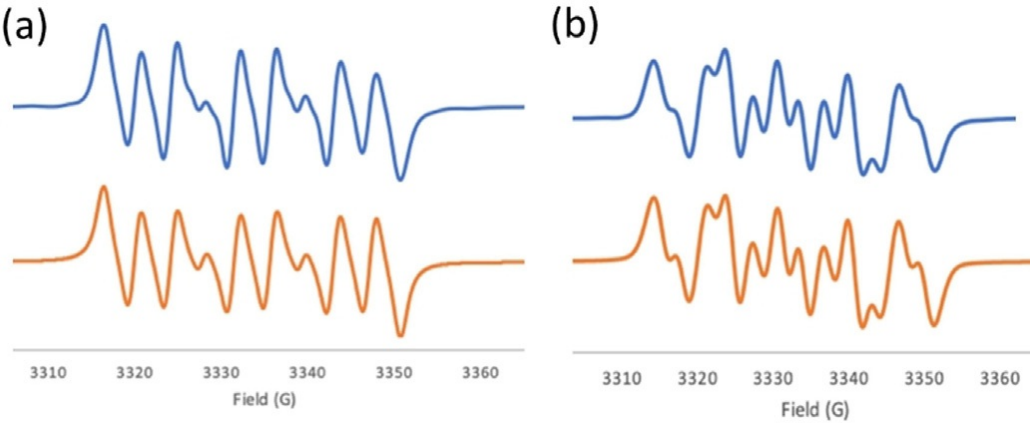
Observed (blue) and simulated (orange) EPR spectra for (a) **6** and (b) **7**.

**Figure 6:**
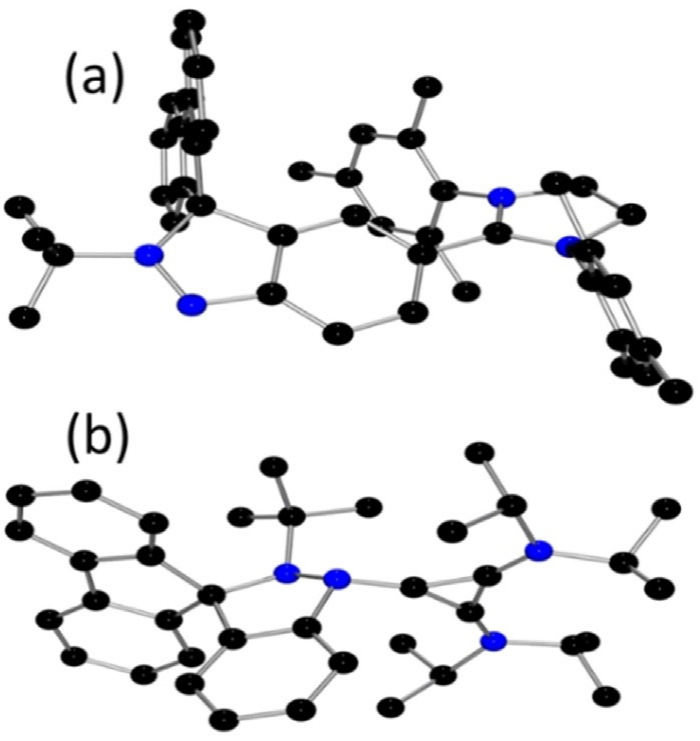
POV‐ray depictions of cations (a) **7** and (b) **8**; hydrogen atoms and BF_4_ anions are omitted for clarity. C: black; N: blue. Selected bond lengths: **7**: N(1)−N(2) 1.350(3), C(6)−C(24) 1.461(4) Å; **8**: N(1)−N(2) 1.505(6), N(1)−C(24) 1.404(7) Å.

To further exploit these cations, **1** was also reacted with 1,3‐bis(2,4,6‐trimethylphenyl)‐4,5‐dihydroimidazol‐2‐ylidene (SIMes) yielding a purple species **6** at 67 % yield (Scheme 4). The ^1^H NMR spectrum of **6** is silent and attempts to characterize radical **6** by X‐ray crystallography were fruitless. However, the EPR spectrum of **6** was recorded and simulated revealing a g value of 2.0049 of which hyperfine couplings of N: 11.50, 4.18, 0.98, 0.33 G and H: 0.59, 0.41, 0.21 G (Figure 5). The corresponding reaction of the *N*‐Lewis acid **2** with the carbene SIMes was performed in *o*‐C_6_F_2_H_4_ affording a red species **7** at 80 % yield. The ^1^H NMR spectrum of **7** is also silent, while the EPR spectrum of **7** was similar to that of **6** with a *g* value of 2.0042 and hyperfine couplings of N: 9.39, 6.67, 0.48, 0.39 G, and H: 2.67, 0.60, 0.53 G (Figure 5). Single crystals for X‐ray diffraction analysis were obtained by layering of pentane into a THF solution of **7** at −35 °C. The solid‐state structure revealed the formulation of **7** as [(SIMes)C_6_H_3_NN(*t*Bu)C(C_12_H_8_)^.^][BF_4_] (Figure 6). The N−N bond length in **7** was found to be 1.350(3) Å, which is longer than that seen in **2**. The unambiguous characterization of **7** suggests an analogous structure for **6** (i.e. [(SIMes)C_6_H_3_N(*t*Bu)NC(C_12_H_8_)^.^][BF_4_]). Indeed, the liberation of H_2_ was confirmed by NMR spectroscopy of the reaction mixtures in a sealed NMR tube in C_6_D_6_.

Compound **2** was also reacted with the sterically smaller, yet more basic carbene, bis(diisopropylamino)‐cyclopropenylidene (BAC), at room temperature. This yielded a black solid **8** in 78 % yield. While the NMR data was consistent with the combination of the two reagents, an X‐ray crystallographic study confirm the formulation of **8** as [C_6_H_4_N(BAC)N(*t*Bu)C(C_12_H_8_)][BF_4_] (Figure 6). In this species, the BAC is coordinated to the electrophilic N(1)‐site leading to pyramidalization of nitrogen atoms and an N−N bond length of 1.505(6) Å. The newly formed N−C bond length was found to be 1.404(7) Å, which is significantly longer than those seen in the carbene‐stabilized nitrogen cation, [(BAC)_3_ n]^3+^ ((1.378(3) Å) reported by Alcarazo and co‐workers.^[54]^

The formation of the radical cations in **6** and **7** suggests that steric demands preclude nucleophilic attack of SIMes at the Lewis‐acidic N(1) site, instead prompting attack at the aryl *para*‐carbon. While this aspect is analogous to the formation of **4** and **5**, the strong σ donation from carbene prompts loss of a H‐atom affording H_2_ and the radical cations. Presumably in the case of BAC, the significantly lesser steric demand results in the classical donor–acceptor bond between the carbene and the Lewis acidic nitrenium cation.

**Figure 7:**
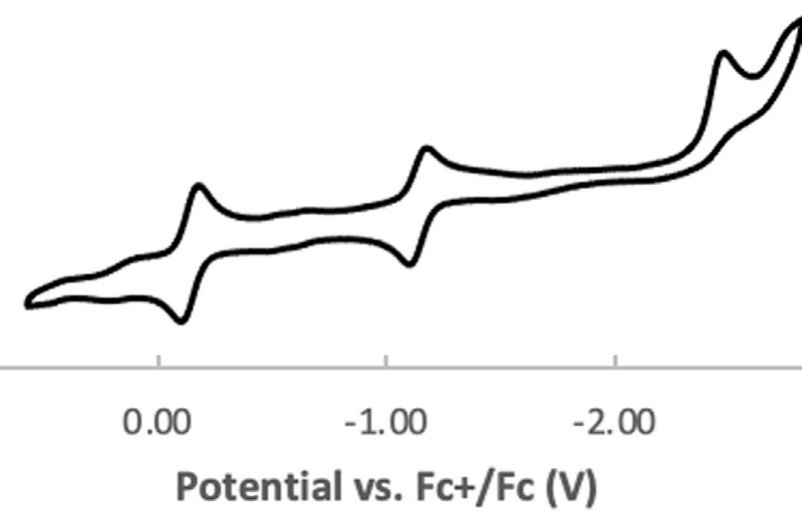
Cyclic voltammogram of compound **7** recording by using [*n*Bu_4_N][BF_4_] as the electrolyte and ferrocene as the standard.

**Scheme 5:**
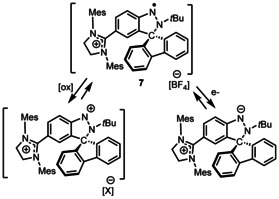
Redox reactivity of **7**.

Interestingly, compounds **6** and **7** exhibit reversible redox behavior at −0.29 and −0.14 V, respectively, as evidenced by the cyclic voltammetry in *o*‐C_6_F_2_H_4_ containing 0.085 m *n*Bu_4_NPF_6_ as a supporting electrolyte (Figures 7 and S42). These were attributed to the facile oxidation of the radical cations to the corresponding dications, reminiscent of the oxidation of aminyl radical reported by Osuka^[30]^ and pyridinyl radicals by Hansman.^[55]^ Compound **7** showed a second reversible redox wave at −1.14 V attribute to the reduction of the cation to the corresponding neutral zwitterionic species.^[56]^ A further reduction of this species is irreversible at −2.47 V. In contrast, compound **6** is reduced irreversibly at about −1.4 V. The high reversible nature of the reduction of **7** are attributed to the stabilization of the zwitterionic species by the adjacent π‐system (Scheme 5).

**Scheme 6:**
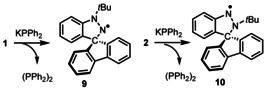
Reactivity of **1** and **2** with KPPh_2_.

**Figure 8:**
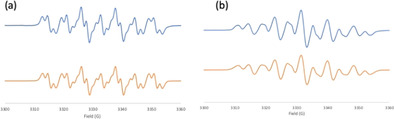
Experimental (blue) and simulated (orange) X‐band EPR spectra of (a) **9** and (b) **10**.

In related efforts compound **1** or **2** were treated with 1 equiv of KPPh_2_ in THF. This prompted the immediate color change to a deep green color while the ^31^P{^1^H} spectrum of the reaction crude showed the formation of (Ph_2_P)_2._ (Figures S35 and S37). Monitoring the reactions by EPR spectroscopy showed signals suggesting the formation of the respective radicals **9** and **10**. These observations suggest a net electron transfer from phosphide to the corresponding nitrenium‐cation and thus the formulations of **9** and **10** as the aminyl radicals C_6_H_4_N(*t*Bu)NC(C_12_H_8_)^.^ and C_6_H_4_NN(*t*Bu)C(C_12_H_8_)^.^, respectively. EPR data for these species were consistent with g values of 2.0038 and 2.0037 for **9** and **10**, respectively (Figures S36 and S38). In addition, spectral simulations revealed hyperfine couplings to two N atoms and four hydrogen atoms, consistent with these formulations (Scheme 6, Figure 8, see the Supporting Information). While these radicals were not isolable in analytically pure form as only spiro[fluorene‐9,3’‐indazole] was recovered, these formulations were further supported by the observation of a reversible reductions in the cyclic voltammograms of **1** and **2** at ca.‐0.70 and −0.54 V vs. Fc^+^/Fc, respectively (see Figures S40 and S41 in the Supporting Information).

The reaction of **1** and **2** with phosphines and carbenes suggests that the reactions with phosphide could involve nucleophilic attack at either the electrophilic *N*‐positions or at the *para*‐carbon atom. It is noteworthy that in related work, Gandelman et al.^[27]^ recently described the analogous reaction involving a N‐based Lewis acid derived from a triazolium derivative with phosphide suggesting that this proceeds via a transient nitrenium‐phosphide adduct. However, DFT calculations for the present reactions of **1** and **2** with dimer (KPPh_2_)_2_ show that the initial electron transfer effects the reductions affording K(PPh_2_)_2_^.^ and **9** and **10** respectively are exothermic by 4.5 and 8.0 kcal mol^−1^ reductions, while further electron transfer to **1** or **2** affording (PPh_2_)_2_ is even more exothermic (−60 kcal mol^−1^) (see the Supporting Information).

## Conclusions

Alkylation of spiro[fluorene‐9,3’‐indazole] with *t*BuCl affords the product of alkylation at the N(1) position, **1**, which undergoes a thermal rearrangement to give alkylation at N(2), **2**. While experimental and computational data show that each of these species are nitrenium cation salts, reactions of these species with various donors demonstrates Lewis acidity at both the nitrenium *N*‐atom and the aryl carbon *para*‐to N. For example, **1** binds PMe_3_ at the Lewis acidic N(2) center to give **3**, while **2** reacts with phosphines at the *para*‐carbon resulting in proton migration to carbon affording **4** and **5**. The bulky, yet stronger donor carbene, SIMes, reacts with **1** or **2** at the *para*‐carbon, inducing loss of H^.^ to give NHC‐substituted radicals cations **6** and **7**. In contrast, the smaller carbene nucleophile, BAC, reacts with **2** to form the classical adduct **8** at the electrophilic N atom. Finally, in reactions of **1** and 2 with KPPh_2_, electron transfer affords the (PPh_2_)_2_ and the corresponding diazo‐radicals **9** and **10**. The access to one and two electron chemistry derived from these nitrenium cation‐based Lewis acids augurs well for the development of a rich chemistry of N‐based Lewis acids. We are continuing to explore related systems, with a particular interest in the applications in FLPs and Lewis acid catalysis.

## Experimental Section

**Crystallographic data**: Deposition number(s) 2035309, 2035310, 2035311, 2035312, 2035313, 2035314, and 2035315 contain the supplementary crystallographic data for this paper. These data are provided free of charge by the joint Cambridge Crystallographic Data Centre and Fachinformationszentrum Karlsruhe Access Structures service.

## Conflict of interest

The authors declare no conflict of interest.

## Supporting information

As a service to our authors and readers, this journal provides supporting information supplied by the authors. Such materials are peer reviewed and may be re‐organized for online delivery, but are not copy‐edited or typeset. Technical support issues arising from supporting information (other than missing files) should be addressed to the authors.

Supplementary

## References

[1] G. N. Lewis , Valence and the Structure of Atoms and Molecules, Chemical Catalogue Company, New York, 1923.

[2] D. Seebach , Angew. Chem. Int. Ed. Engl. 1979, 18, 239–258;

[3] D. Seebach , D. Enders , Angew. Chem. Int. Ed. Engl. 1975, 14, 15–32;

[4] X. Bugaut , F. Glorius , Chem. Soc. Rev. 2012, 41, 3511–3522. 22377957 10.1039/c2cs15333e

[5] D. W. Stephan , Angew. Chem. Int. Ed. 2017, 56, 5984–5992; 10.1002/anie.201700721 28195386

[6] D. Enders , O. Niemeier , A. Henseler , Chem. Rev. 2007, 107, 5606–5655. 17956132 10.1021/cr068372z

[7] D. M. Flanigan , F. Romanov-Michailidis , N. A. White , T. Rovis , Chem. Rev. 2015, 115, 9307–9387. 25992594 10.1021/acs.chemrev.5b00060 PMC4986729

[8] M. N. Hopkinson , C. Richter , M. Schedler , F. Glorius , Nature 2014, 510, 485–496. 24965649 10.1038/nature13384

[9] V. Nair , R. S. Menon , A. T. Biju , C. R. Sinu , R. R. Paul , A. Jose , V. Sreekumar , Chem. Soc. Rev. 2011, 40, 5336–5346. 21776483 10.1039/c1cs15139h

[10] M. H. Wang , K. A. Scheidt , Angew. Chem. Int. Ed. 2016, 55, 14912–14922; 10.1002/anie.201605319 27763702

[11] C. Zhang , J. F. Hooper , D. W. Lupton , ACS Catal. 2017, 7, 2583–2596.

[12] A. R. Jupp , D. W. Stephan , Trends Chem. 2019, 1, 35–48.

[13] D. W. Stephan , Science 2016, 354, aaf7229.27940818

[14] D. W. Stephan , G. Erker , Angew. Chem. Int. Ed. 2015, 54, 6400–6441; 10.1002/anie.201409800 25974714

[15] D. W. Stephan , J. Am. Chem. Soc. 2015, 137, 10018–10032. 26214241 10.1021/jacs.5b06794

[16] D. W. Stephan , Acc. Chem. Res. 2015, 48, 306–316. 25535796 10.1021/ar500375j

[17] C. B. Caputo , L. J. Hounjet , R. Dobrovetsky , D. W. Stephan , Science 2013, 341, 1374–1377. 24052304 10.1126/science.1241764

[18] J. M. Bayne , D. W. Stephan , Chem. Soc. Rev. 2016, 45, 765–774. 26255595 10.1039/c5cs00516g

[19] D. E. Falvey , Nitrenes and Nitrenium Ions, Wiley, New York, 2013, pp. 191–216.

[20] J. L. Duffy , K. K. Laali , J. Org. Chem. 1991, 56, 3006–3009.

[21] S. Moebs-Sanchez , G. Bouhadir , N. Saffon , L. Maron , D. Bourissou , Chem. Commun. 2008, 3435–3437. 10.1039/b805161e 18633514

[22] G. A. Olah , J. A. Olah , N. A. Overchuk , J. Org. Chem. 1965, 30, 3373–3376.

[23] F. Dielmann , O. Back , M. Henry-Ellinger , P. Jerabek , G. Frenking , G. Bertrand , Science 2012, 337, 1526–1528. 22997335 10.1126/science.1226022

[24] Y. Tulchinsky , M. A. Iron , M. Botoshansky , M. Gandelman , Nat. Chem. 2011, 3, 525. 21697872 10.1038/nchem.1068

[25] Y. Tulchinsky , S. Kozuch , P. Saha , M. Botoshansky , L. J. W. Shimon , M. Gandelman , Chem. Sci. 2014, 5, 1305–1311. 10.1002/chem.201405526 25783449

[26] Y. Tulchinsky , S. Kozuch , P. Saha , A. Mauda , G. Nisnevich , M. Botoshansky , L. J. W. Shimon , M. Gandelman , Chem. Eur. J. 2015, 21, 7099–7110. 25783449 10.1002/chem.201405526

[27] A. Pogoreltsev , Y. Tulchinsky , N. Fridman , M. Gandelman , J. Am. Chem. Soc. 2017, 139, 4062–4067. 28240551 10.1021/jacs.6b12360

[28] M. Mehta , J. M. Goicoechea , Angew. Chem. Int. Ed. 2020, 59, 2715–2719; 10.1002/anie.201915547 31808974

[29] I. Avigdori , A. Pogoreltsev , A. Kaushanski , N. Fridman , M. Gandelman , Angew. Chem. Int. Ed. 2020, 59, 23476–23479; 10.1002/anie.202008798 33405343

[30] D. Shimizu , K. Fujimoto , A. Osuka , Angew. Chem. Int. Ed. 2018, 57, 9434–9438; 10.1002/anie.201805385 29882340

[31] J. L. Zhou , L. L. Liu , L. L. Cao , D. W. Stephan , Angew. Chem. Int. Ed. 2018, 57, 3322–3226; 10.1002/anie.201713118 29397010

[32] S. S. Ullah , C. Kashyap , A. K. Guha , ChemistrySelect 2018, 3, 6715–6718.

[33] J. Zhou , L. L. Liu , L. Cao , D. W. Stephan , Chem. Commun. 2018, 54, 4390–4393. 10.1039/c8cc01331d 29527605

[34] A. E. Waked , R. O. Memar , D. W. Stephan , Angew. Chem. Int. Ed. 2018, 57, 11934–11938; 10.1002/anie.201804183 29806886

[35] **2018**, TURBOMOLE, version 7.3, See http://www.turbomole.com, TURBOMOLE GmbH, Karlsruhe.

[36] J. Tao , J. P. Perdew , V. N. Staroverov , G. E. Scuseria , Phys. Rev. Lett. 2003, 91, 146401. 14611541 10.1103/PhysRevLett.91.146401

[37] S. Grimme , Chem. Eur. J. 2012, 18, 9955. 22782805 10.1002/chem.201200497

[38] S. Grimme , J. Antony , S. Ehrlich , H. Krieg , J. Chem. Phys. 2010, 132, 154104. 20423165 10.1063/1.3382344

[39] S. Grimme , S. Ehrlich , L. Goerigk , J. Comput. Chem. 2011, 32, 1456–1465. 21370243 10.1002/jcc.21759

[40] P. Deglmann , K. May , F. Furche , R. Ahlrichs , Chem. Phys. Lett. 2004, 384, 103.

[41] F. Weigend , M. Häser , H. Patzelt , R. Ahlrichs , Chem. Phys. Lett. 1998, 143, 294.

[42] F. Weigend , F. Furche , R. Ahlrichs , J. Chem. Phys. 2003, 119, 12753.

[43] F. Weigend , R. Ahlrichs , Phys. Chem. Chem. Phys. 2005, 7, 3297–3305. 16240044 10.1039/b508541a

[44] K. Eichkorn , F. Weigend , O. Treutler , R. Ahlrichs , Theor. Chem. Acc. 1997, 97, 119.

[45] **2015**, COSMOtherm, Version C3.0, Release 16.01, COSMOlogic GmbH & Co., Leverkusen, Germany.

[46] F. Eckert , A. Klamt , AIChE J. 2002, 48, 369–385.

[47] A. Klamt , G. Schüürmann , J. Chem. Soc. Perkin Trans. 1993, 799.

[48] F. Weigend , Phys. Chem. Chem. Phys. 2006, 8, 1057–1065. 16633586 10.1039/b515623h

[49] Y. Zhao , D. G. Truhlar , J. Phys. Chem. A 2005, 109, 5656–5667. 16833898 10.1021/jp050536c

[50] T. Araki , M. Hirai , A. Wakamiya , W. E. Piers , S. Yamaguchi , Chem. Lett. 2017, 46, 1714–1717.

[51] F. H. Allen , O. Kennard , D. G. Watson , L. Brammer , A. G. Orpen , R. Taylor , J. Chem. Soc. Perkin Trans. 2 1987, S1–S19.

[52] G. C. Welch , R. R. S. Juan , J. D. Masuda , D. W. Stephan , Science 2006, 314, 1124–1126. 17110572 10.1126/science.1134230

[53] G. C. Welch , J. D. Masuda , D. W. Stephan , Inorg. Chem. 2006, 45, 478–480. 16411674 10.1021/ic051713r

[54] A. Kozma , G. Gopakumar , C. Fares , W. Thiel , M. Alcarazo , Chem. Eur. J. 2013, 19, 3542–3546. 23436732 10.1002/chem.201204186

[55] P. W. Antoni , T. Bruckhoff , M. M. Hansmann , J. Am. Chem. Soc. 2019, 141, 9701–9711. 31125215 10.1021/jacs.9b04249

[56] D. Rottschäfer , B. Neumann , H. G. Stammler , M. van Gastel , D. M. Andrada , R. S. Ghadwal , Angew. Chem. Int. Ed. 2018, 57, 4765–4768; 10.1002/anie.201801596 29465816

