# Supplementary material for: The Reactivity of Isomeric Nitrenium Lewis Acids with Phosphines, Carbenes, and Phosphide
Source: Chemistry. 2021 Jan 14;27(8):2861–7. doi: 10.1002/chem.202004798 (PMC7898612; doi:10.1002/chem.202004798)
Supplement: Supplementary file 1 — Supplementary [file CHEM-27-2861-s001.pdf]

Chemistry–A European Journal

Supporting Information

The Reactivity of Isomeric Nitrenium Lewis Acids with Phosphines, Carbenes, and Phosphide

Diya Zhu,[a] Zheng-Wang Qu,*[b] Jiliang Zhou,[a] and Douglas W. Stephan*[a]

This PDF file includes:

1. General Remarks 2
2. Synthesis and Characterisations 2
    2.1 Compound 1 and 2 2
    2.2 Compound 3 7
    2.3 Compound 4 10
    2.4 Compound 5 13
    2.5 Compound 6 16
    2.6 Compound 7 17
    2.7 Compound 8 18
3. Crystallographic data 20
4. Kinetic data 23
5. Observation of the radical 9 25
6. Observation of the radical 10 26
7. Observation of H_2 in reaction of 2 and SIMes 27
8. **Electrochemistry** **27**
9. **Computational details** **29**

1. General Remarks

All reactions and work-up procedures were performed under an inert atmosphere of dry, oxygen-free N_2 by means of standard Schlenk techniques or glovebox techniques (MBraun glovebox equipped with a -35 °C freezer) unless otherwise specified. All glassware was oven-dried and cooled under vacuum before use. Dichloromethane (CH_2Cl_2), 1,2-difluorobenzene (ODFB), and toluene were distilled over CaH_2 and tetrahydrofuran (THF) was distilled over Na/benzophenone. Pentane and hexane were collected from a Grubbs-type column system manufactured by Innovative Technology and degassed. Solvents were stored over activated 4 Å molecular sieves. Molecular sieves, type 4 Å (pellets, 3.2 mm diameter) purchased from Sigma Aldrich were activated prior to usage by iteratively heating under vacuum for 24 hours. $CDCl_3$ purchased from Cambridge Isotope Laboratories was vacuum distilled over CaH_2. Unless otherwise mentioned, chemicals were purchased from Sigma Aldrich or TCI. Spiro[fluorene-9,3'-indazole],[S1] 1,3-Bis(2,4,6-trimethylphenyl)-4,5-dihydroimidazol-2-ylidene (SIMes),[S2] and bis(diisopropylamino)cyclopropenylidene (BAC)[S3] were prepared according to previously reported synthetic procedures. Tertbutyl chloride was degassed and stored over activated 4 Å molecular sieves in a Schlenk flask prior to use. NMR spectra were recorded at room temperature (298K) unless otherwise mentioned on a Bruker Avance III 400 MHz, an Agilent DD2 500, and an Agilent DD2 600 Spectrometers. Spectra were referenced to the residual solvent signals ($CDCl_3$: ^{1}H= 7.26 ppm and ^{13}C = 77.2 ppm). Chemical shifts (δ) are reported in ppm and coupling constants (*J*) are listed as absolute values in Hz. Multiplicities are reported as singlet (s), doublet (d), triplet (t), quartet (q), multiplet (m), overlapping (ov), and broad (br). Electron paramagnetic resonance (EPR) measurements were performed at 298 K using a Bruker ECS-EMX X-band EPR spectrometer equipped with an EP4119HS cavity. Simulations were performed using PEST WinSIM software. High resolution mass spectrometry was performed in house employing electrospray ionisation techniques in positive ion mode on an AB/Sciex QStarXL mass spectrometer (ESI).

S1. G. Baum and H. Shechter, *J. Org. Chem.* **1976**, *12*, 2120.
S2. X. Bantreil and S. P. Nolan, *Nature Protocols* **2011,** *6*, 69.
S3. V. Lavallo, Y. Canac, B. Donnadieu, W. W. Schoeller, and G. Bertrand, *Science* **2006**, *5774*, 722.

2. Synthesis and Characterisations

2.1 Compound 1 and 2

Tert-butyl chloride (165 μL, 1.50 mmol) was added dropwise to a dichloromethane solution of spiro[fluorene-9,3'-indazole] (335.4 mg, 1.25 mmol) and $AgBF_4$ (243.3 mg, 1.25 mmol). Upon addition, the solution turned to dark red with white precipitates and was allowed to stir at room temperature for 10 min. The suspension was filtered and the volatiles were removed under vacuum. The orange solid was washed with dichloromethane and toluene (v/v=1/4), dried under vacuum. The crude mixture was again dissolved in dichloromethane (2 mL), layer with pentane (0.8 mL), and stored at -35°C overnight. Compound **1** was decanted, dried under vacuum, and obtained as bright orange crystals. To the remaining aliquot, 1 mL of pentane was added and the solution was stored at -35°C overnight again. Compound **2** was decanted, dried under vacuum, and obtained as a dusty orange powder. If

1 and **2** still have a trace amount of impurities, further recrystallization from saturated CH_2Cl_2 solution layering with pentane would yield pristine products.

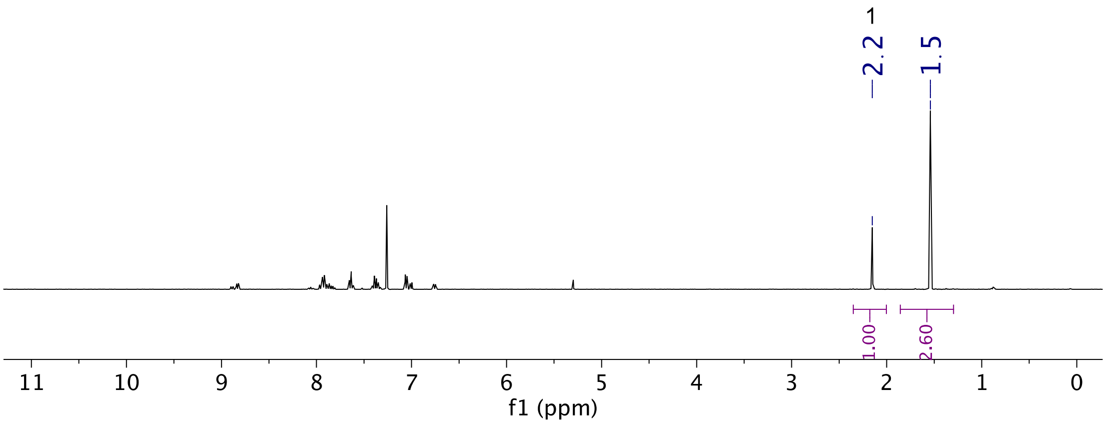

Figure S1: ^{1}H ($CDCl_3$) NMR spectrum of the crude mixture.

1:

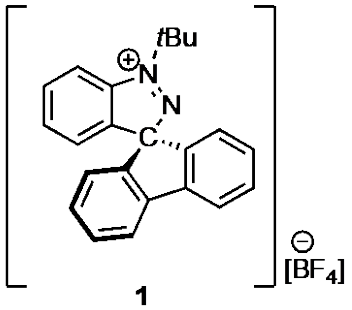

Yield: 122.6 mg (24% isolated yield). ^{1}H NMR (400 MHz, $CDCl_3$): δ 8.91 (d, $^3J_{H\text{-}H}$ = 8.6 Hz, Ar, 1H), 8.07 (t, $^3J_{H\text{-}H}$ = 8.1 Hz, Ar, 1H), 7.96 (d, $^3J_{H\text{-}H}$ = 7.8 Hz, Ar, 2H), 7.83 (t, $^3J_{H\text{-}H}$ = 7.6 Hz, Ar, 1H), 7.64 (t, $^3J_{H\text{-}H}$ = 7.6 Hz, Ar, 2H), 7.35 (ov, Ar, 3H), 6.76 (d, $^3J_{H\text{-}H}$ = 7.8 Hz, Ar, 2H), 2.16 (s, *t*Bu, 9H). ^{11}B{^{1}H} NMR (128 MHz, $CDCl_3$): δ -0.9 (s). ^{19}F{^{1}H} NMR (377 MHz, $CDCl_3$): δ -152.8 (s). ^{13}C{^{1}H} NMR (101 MHz, $CDCl_3$) δ 145.6, 144.7, 144.1, 136.0, 132.9, 132.3, 132.1, 130.0, 125.0, 124.6, 122.4, 122.4, 97.1 (fluorene-C), 79.5, 29.3. ESI MS: m/z: 325.1699 (calcd for M+: 325.1699).

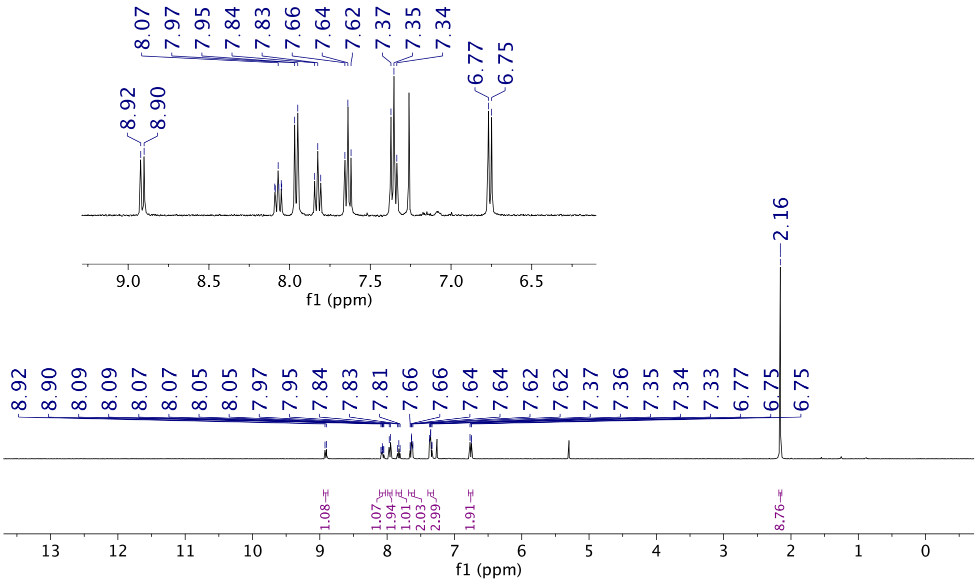

Figure S2: ^{1}H ($CDCl_3$) NMR spectrum of **1**.

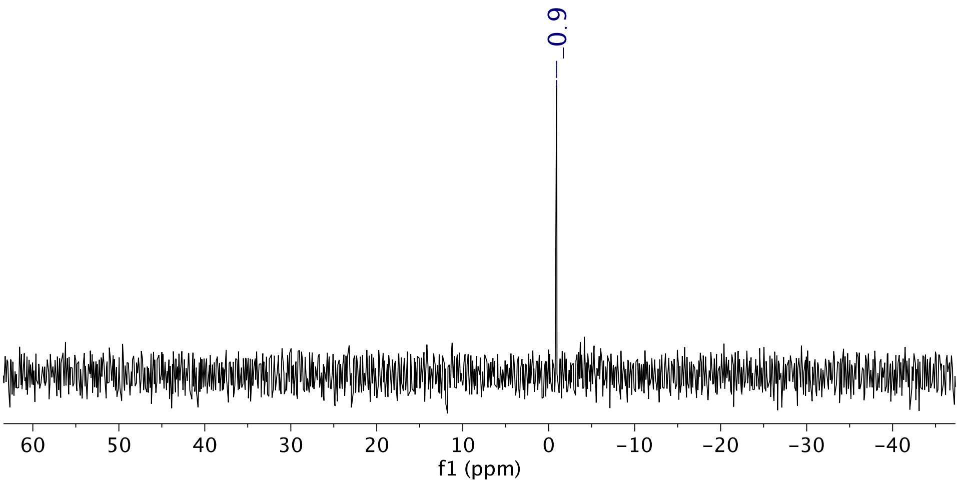

Figure S3: $^{11}B\{^{1}H\}$ ($CDCl_3$) NMR spectrum of **1**.

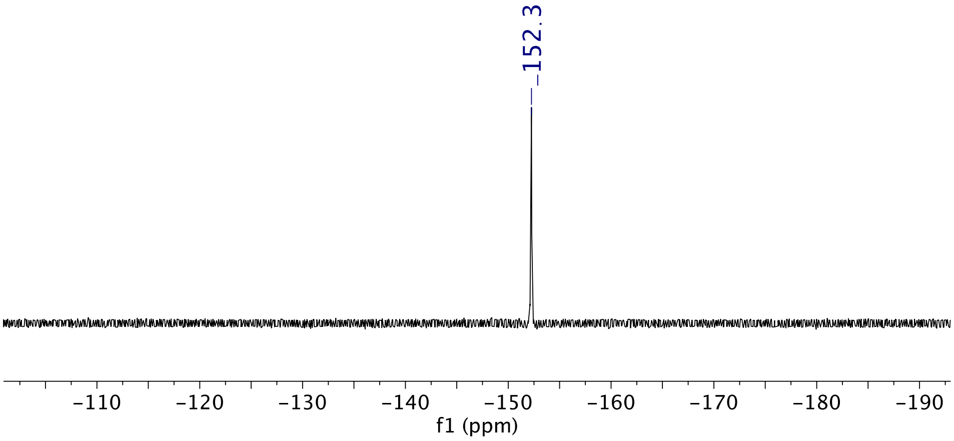

Figure S4: $^{19}F\{^1H\}$ ($CDCl_3$) NMR spectrum of **1**.

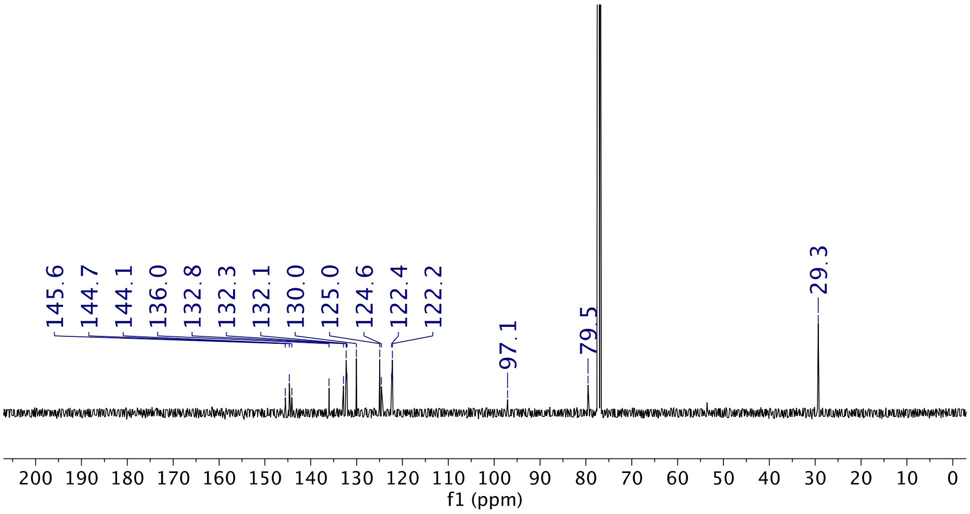

Figure S5: $^{13}C\{^1H\}$ ($CDCl_3$) NMR spectrum of **1**.

2:

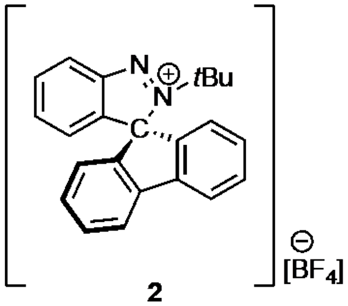

2

Yield: 336.1 mg (65% isolated yield). ^1H NMR (400 MHz, $CDCl_3$): δ 8.83 (d, $^3J_{H-H}$ = 8.1 Hz, Ar, 1H), 8.00 – 7.82 (ov, Ar, 4H), 7.64 (td, $^3J_{H-H}$ = 7.6 Hz, $^4J_{H-H}$ = 1.0 Hz, Ar, 2H), 7.39 (td, $^3J_{H-H}$ = 7.6 Hz, $^4J_{H-H}$ = 1.0 Hz, Ar, 2H), 7.05 (dd, $^3J_{H-H}$ = 7.8 Hz, $^4J_{H-H}$ = 0.9 Hz, Ar, 2H), 7.01 (d, $^3J_{H-H}$ = 6.6 Hz, Ar, 1H), 1.54 (s, *t*Bu, 9H).$^{11}B\{^1H\}$ NMR (128 MHz, $CDCl_3$): δ -1.0 (s). $^{19}F\{^1H\}$ NMR (377 MHz, $CDCl_3$): δ -152.5 (s). $^{13}C\{^1H\}$ NMR (126 MHz, $CDCl_3$): δ 151.1, 143.1, 142.5, 140.2, 133.0, 132.6, 132.5, 130.4, 129.9, 125.1, 122.5, 122.1, 99.6 (fluorene-C), 79.7, 30.1. ESI MS: m/z: 325.1693 (calcd for M+: 325.1699).

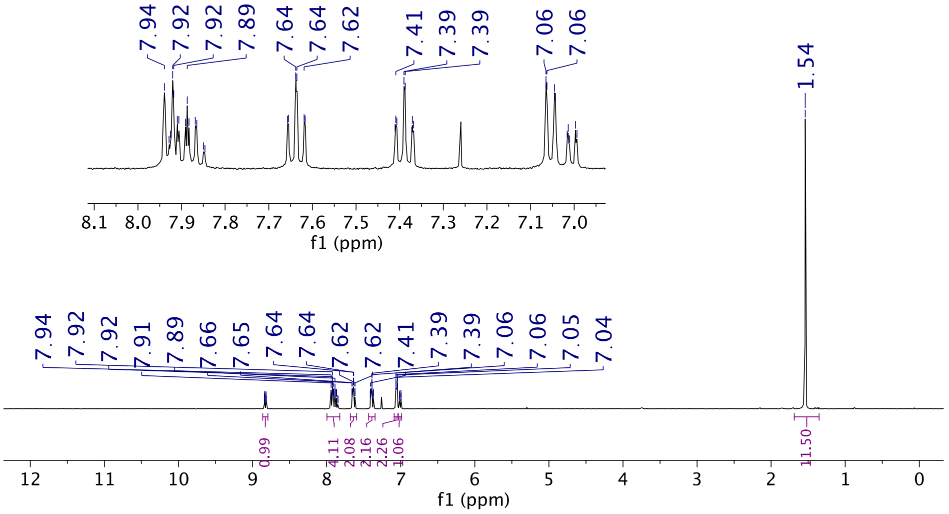

Figure S5: ^{1}H ($CDCl_3$) NMR spectrum of **2**.

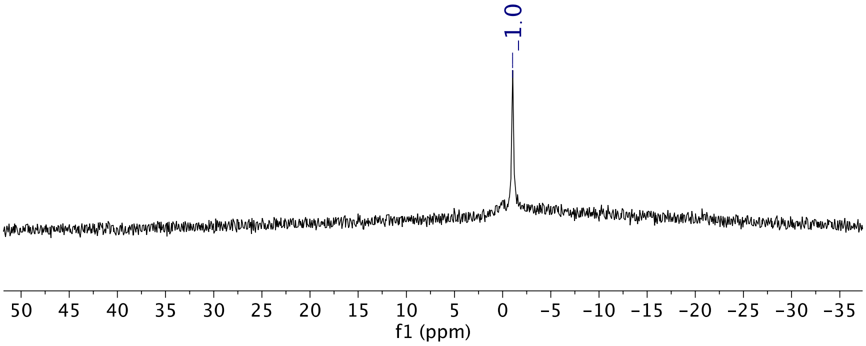

Figure S6: ^{11}B{^{1}H} ($CDCl_3$) NMR spectrum of **2**.

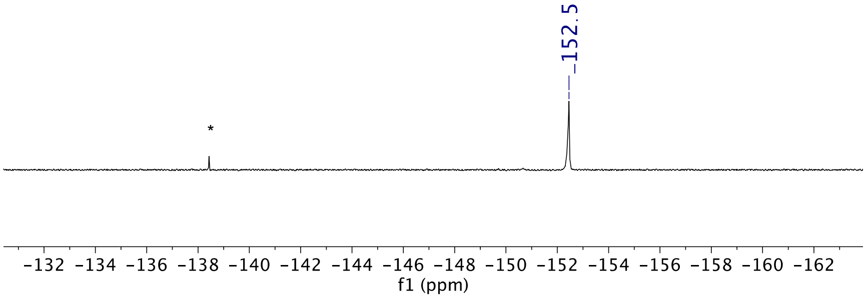

Figure S7: ^{19}F{^{1}H} ($CDCl_3$) NMR spectrum of **2** (Asterisk denoted ODFB impurity in $CDCl_3$).

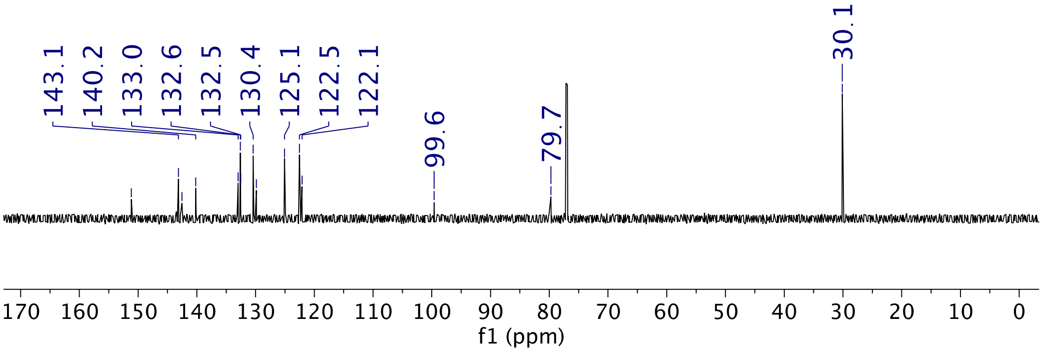

Figure S8: $^{13}C\{^{1}H\}$ ($CDCl_3$) NMR spectrum of **2**.

2.2 Compound 3

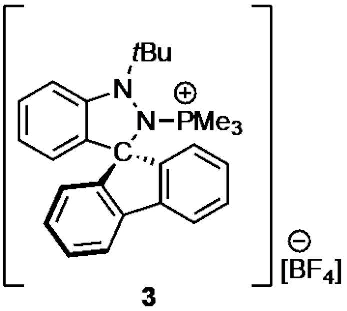

3

To a solution of **1** (41.2 mg, 0.10 mmol) in dichloromethane (3 mL), a THF solution of PMe_3 (1.0 M, 0.12 mL, 0.12 mmol) was added dropwise. The solution was allowed to stir at ambient temperature for 10 mins. All volatiles were removed *in vacuo* and the residue was washed with hexane (2 X 1 mL) and dried under vacuum. Compound **3** was obtained as a white powder. Single crystals suitable for X-ray diffraction were grown from liquid diffusion of pentane into a saturated dichloromethane solution at -35 °C. Yield: 46.3 mg (95% isolated yield). ^{1}H NMR (400 MHz, $CDCl_3$): δ 7.76 (t, $^{3}J_{H-H}$ = 8.1 Hz, Ar, 2H), 7.59 – 7.46 (m, Ar, 3H), 7.42 – 7.22 (m, Ar, 4H), 7.11 (td, $^{3}J_{H-H}$ = 7.5 Hz, $^{4}J_{H-H}$ = 1.1 Hz, Ar, 1H), 6.80 (d, $^{3}J_{H-H}$ = 7.7 Hz, Ar, 1H), 6.63 (d, $^{3}J_{H-H}$ = 7.7 Hz, Ar, 1H), 1.74 (d, $^{2}J_{P-H}$ = 12.8 Hz, PMe_3, 9H), 1.40 (s, *t*Bu, 9H). $^{11}B\{^{1}H\}$ NMR (128 MHz, $CDCl_3$): δ -1.1 (s). $^{31}P\{^{1}H\}$ NMR (162 MHz, $CDCl_3$): δ 62.8 (s). $^{13}C\{^{1}H\}$ NMR (126 MHz, CDCl3) δ 150.1 (d, J_{C-P} = 2.2 Hz), 146.9 (d, J_{C-P} = 7.0 Hz), 145.3 (d, J_{C-P} = 6.8 Hz), 142.2, 138.7, 135.9, 132.1, 130.4, 128.9, 128.7, 128.3, 126.8, 126.4, 125.5, 123.7, 121.1 (d, J_{C-P} = 11.6 Hz), 116.8, 81.3 (d, J_{C-P} = 3.2 Hz), 64.2 (d, J_{C-P} = 5.8 Hz), 30.2, 13.2 (d, J_{C-P} = 64.1 Hz). ESI MS: m/z: 401.2141 (calcd for M+: 401.2141).

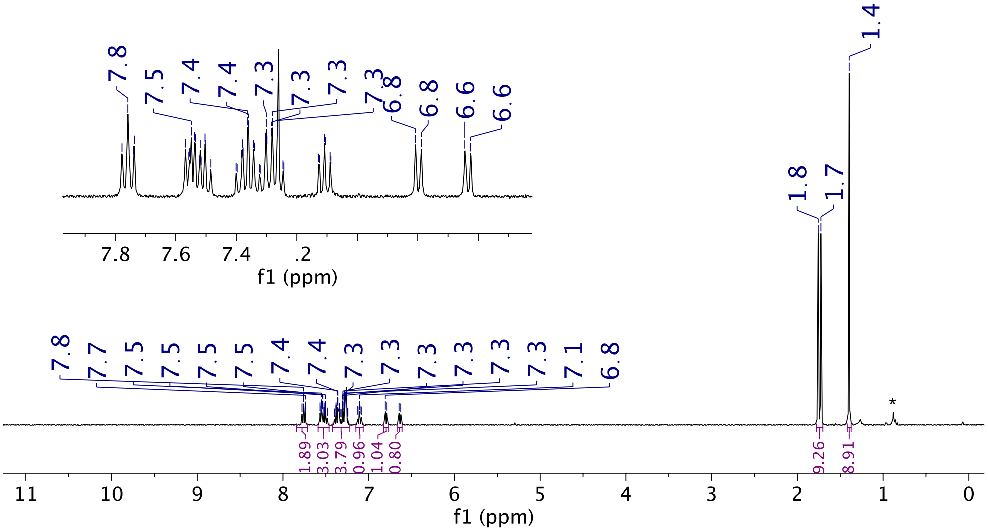

Figure S9: ^{1}H ($CDCl_3$) NMR spectrum of **3** (Asterisk denoted hexane).

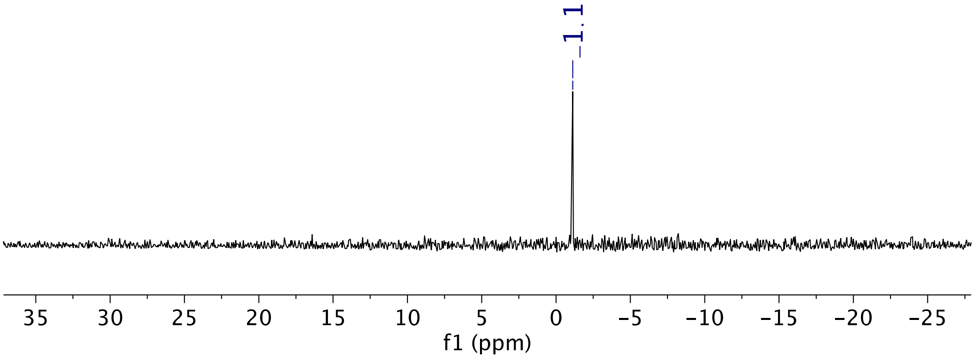

Figure S10: $^{11}B\{^{1}H\}$ ($CDCl_3$) NMR spectrum of **3**.

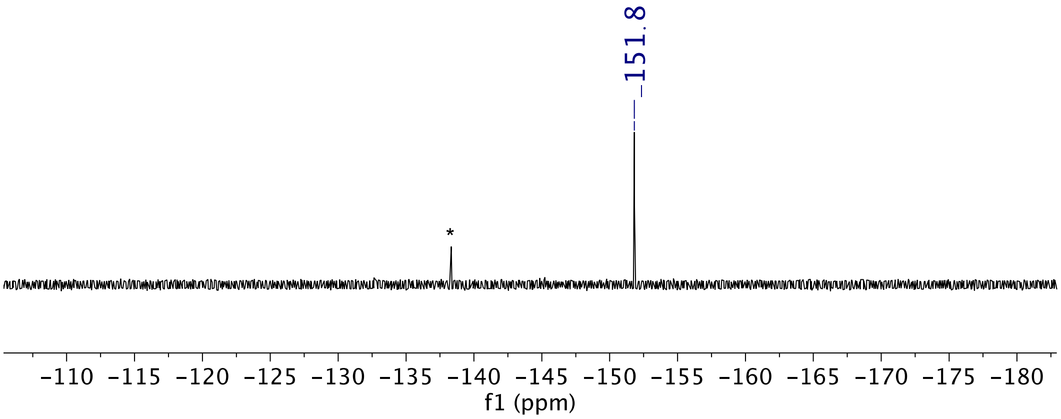

Figure S11: $^{19}F\{^1H\}$ ($CDCl_3$) NMR spectrum of **3**. (Asterisk denoted ODFB residue in $CDCl_3$)

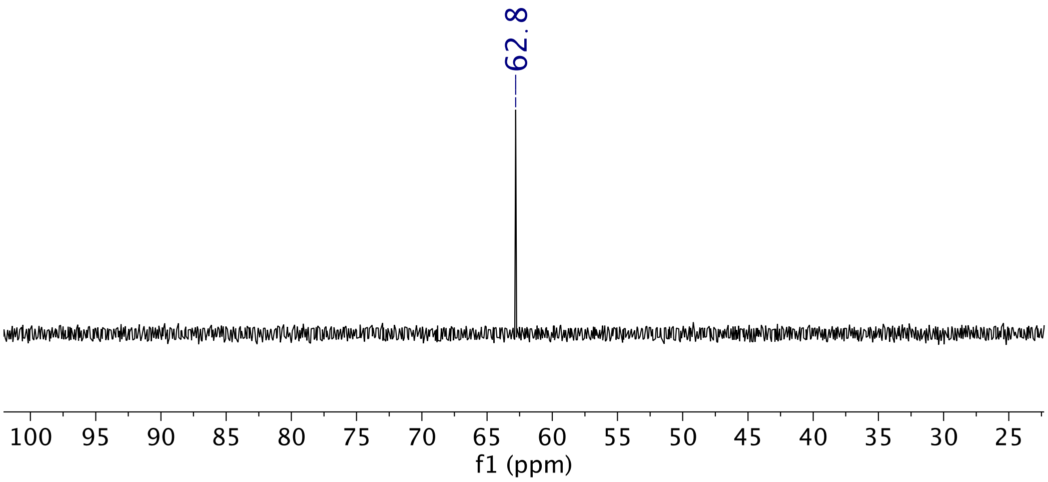

Figure S12: $^{31}P\{^1H\}$ ($CDCl_3$) NMR spectrum of **3**.

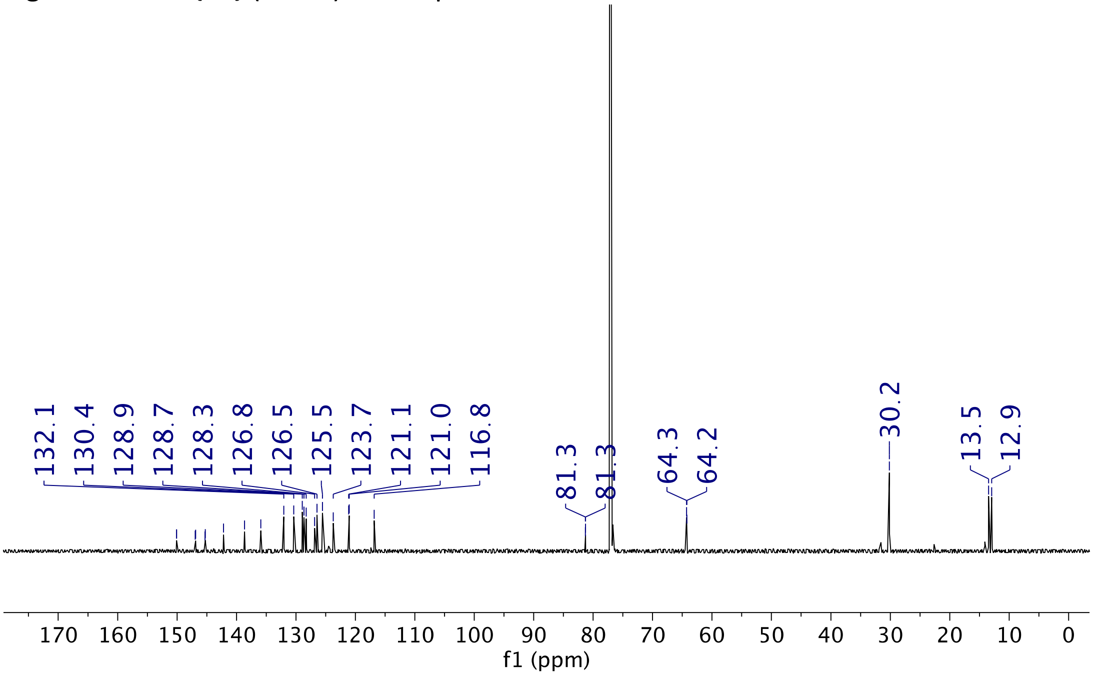

Figure S13: $^{11}C\{^1H\}$ ($CDCl_3$) NMR spectrum of **3**.

2.3 Compound 4

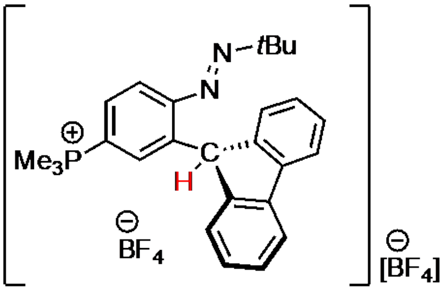

4

To a solution of **2** (41.2 mg 0.1 mmol) in dichloromethane (3 mL), a THF solution of PMe_3 (1.0 M, 0.12 mL, 0.12 mmol) was added dropwise. The solution was allowed to stir at room temperature for 10 min. All volatiles were removed *in vacuo* and the residue was washed with hexane (2 X 1 mL) and dried under vacuum. Compound **4** was obtained as an orange powder. Crystals were grown from liquid diffusion of pentane into a saturated dichloromethane solution at room temperature. Yield: 44.1 mg (92% isolated yield). ^{1}H NMR (400 MHz, $CDCl_3$) δ 7.87 – 7.78 (ov, 4H), 7.68 (m, 1H), 7.44 (t, $^3J_{H\text{-}H}$ = 7.5 Hz, 2H), 7.33 – 7.16 (m, ov with $CDCl_3$ signal, proposed 3H), 6.78 (d, $^3J_{H\text{-}P}$ = 13.5 Hz, 1H), 6.28 (s, CH, 1H), 2.00 (d, $^2J_{H\text{-}P}$ = 14.2 Hz, PMe_3, 9H), 1.46 (s, *t*Bu, 9H). ^{11}B{^{1}H} NMR (128 MHz, $CDCl_3$): δ -1.0 (s). ^{31}P{^{1}H} NMR (162 MHz, $CDCl_3$): δ 22.0 (s). ^{19}F{^{1}H} NMR (377 MHz, $CDCl_3$): δ -149.6. ^{13}C{^{1}H} NMR (126 MHz, $CDCl_3$): δ 154.2, 146.6, 141.8 (d, $J_{C\text{-}P}$ = 12.9 Hz), 141.4, 130.5 (d, $J_{C\text{-}P}$ = 11.3 Hz), 129.7 (d, $J_{C\text{-}P}$ = 11.3 Hz), 128.0 (d, $J_{C\text{-}P}$ = 31.9 Hz), 125.3, 123.5, 122.8, 120.5, 118.9 (d, $J_{C\text{-}P}$ = 13.4 Hz), 70.4, 47.2 (fluorene-C), 27.1, 9.3 (d, $J_{C\text{-}P}$ = 56.8 Hz). ESI MS: m/z: 401.2145 (calcd for M+: 401.2141).

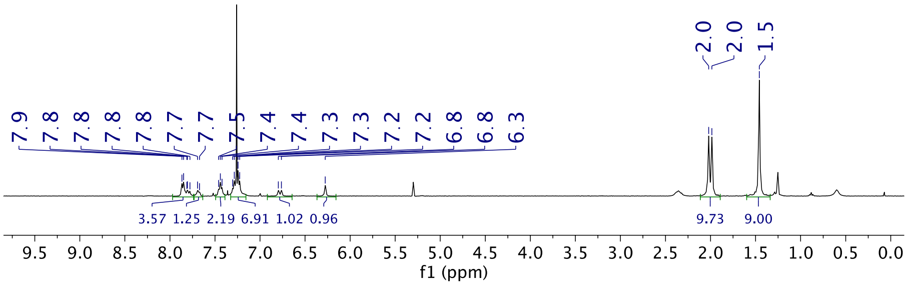

Figure S14: ^{1}H ($CDCl_3$) NMR spectrum of **4**.

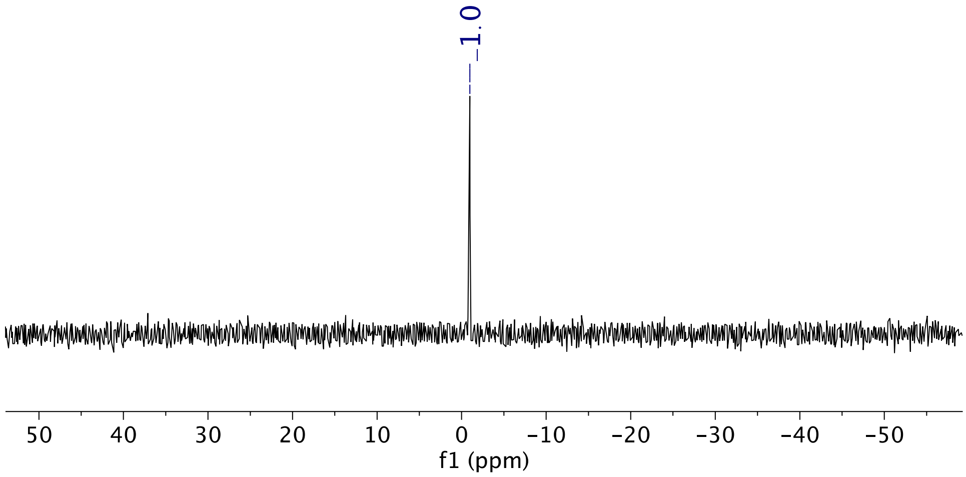

Figure S15: $^{11}B\{^{1}H\}$ ($CDCl_3$) NMR spectrum of **4**.

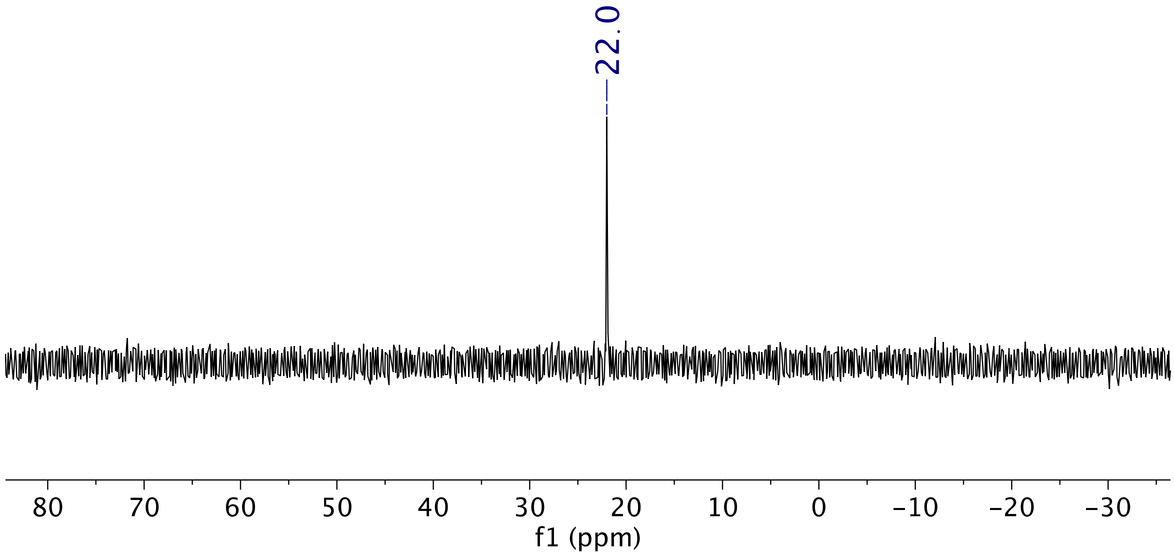

Figure S16: $^{31}P\{^{1}H\}$ ($CDCl_3$) NMR spectrum of **4**.

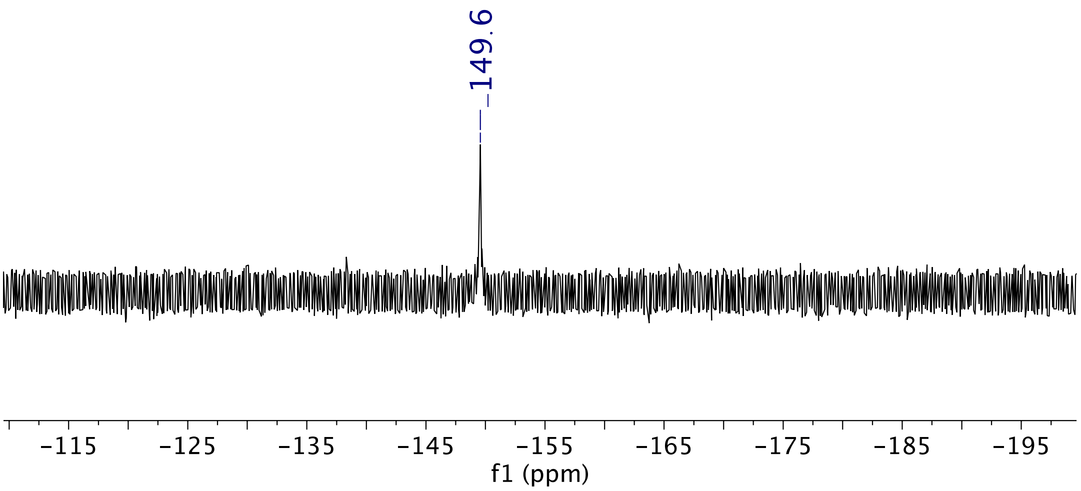

Figure S17: $^{19}F\{^1H\}$ ($CDCl_3$) NMR spectrum of **4**.

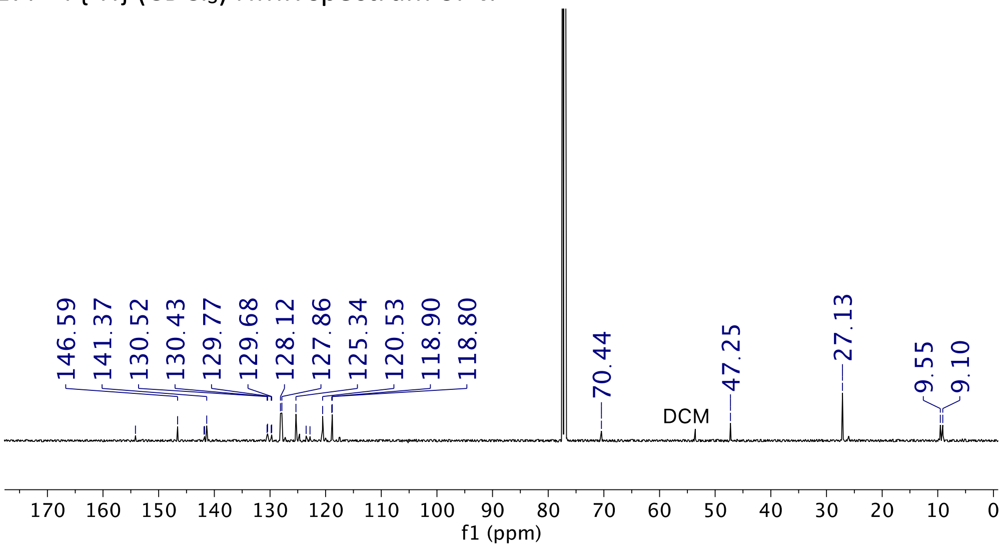

Figure S18: $^{13}C\{^1H\}$ ($CDCl_3$) NMR spectrum of **4**.

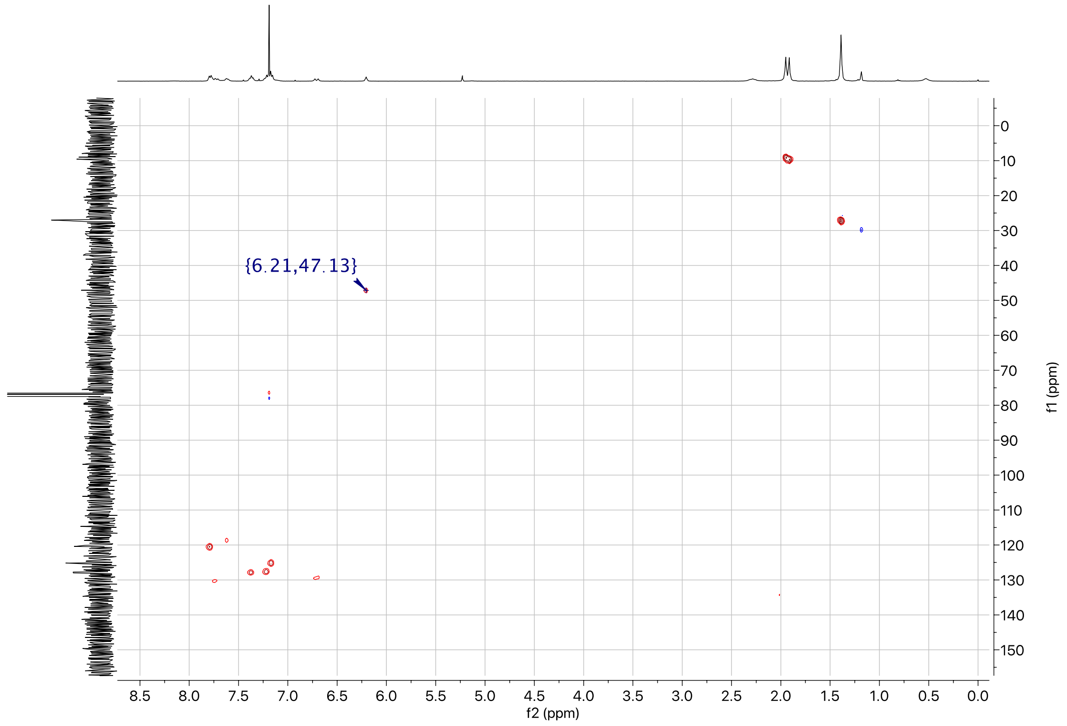

Figure S19: HSQC 2D ($CDCl_3$) NMR spectrum of **4**.

2.4 Compound 5

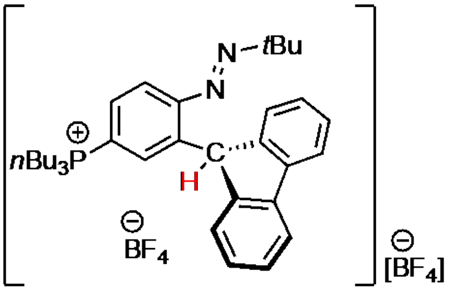

5

To a solution of **2** (41.2 mg 0.1 mmol) in dichloromethane (3 mL), *n*Bu$_3$P (22.7 mg, 0.11 mmol) was added dropwise. The solution was allowed to stir at room temperature for 10 min. All volatiles were removed *in vacuo* and the residue was washed with hexane (2 X 1 mL) and dried under vacuum. Compound **5** was obtained as an orange powder. Single crystals suitable for X-ray diffraction were grown from liquid diffusion of pentane into a saturated dichloromethane solution at -35 °C. Yield: 241.3 mg (95% isolated yield). ^{1}H NMR (500 MHz, $CDCl_3$): δ 7.86 (d, $^3J_{H-H}$ = 7.7 Hz, Ar, 2H), 7.76 – 7.68 (ov, Ar, 2H), 7.44 (t, $^3J_{H-H}$ = 7.4 Hz, Ar, 2H), 7.31 – 7.21 (ov, Ar, 4H), 6.65 (d, $^3J_{H-P}$ = 12.3 Hz, Ar, 1H), 6.28 (s, CH, 1H), 2.27 (td, $^3J_{H-P}$ = 12.2 Hz, $^3J_{H-H}$ = 7.0 Hz, P*n*Bu$_3$, 6H), 1.48 (s, *t*Bu, 9H), 1.25 (dt, $^2J_{H-P}$ = 32.0 Hz, $^3J_{H-H}$ = 8.3 Hz, P*n*Bu$_3$, 12H), 0.77 (t, $^3J_{H-H}$ = 7.2 Hz, , P*n*Bu$_3$, 9H). ^{11}B{^{1}H} NMR (128 MHz, $CDCl_3$): δ -1.0 (s). ^{31}P{^{1}H} NMR (162 MHz, $CDCl_3$): δ 29.9 (s). ^{19}F{^{1}H} NMR (377 MHz, $CDCl_3$): δ -151.4. ^{13}C{^{1}H} NMR (126 MHz, $CDCl_3$): δ 153.8, 146.9, 141.9 (d, J_{C-P} = 11.4 Hz), 141.2, 132.4, 131.4 (d, J_{C-P} = 9.3 Hz), 130.9 (d, J_{C-P} = 9.1 Hz), 127.8 (d, J_{C-P} = 39.5 Hz), 125.1, 120.4, 118.9 (d, J_{C-P} = 12.3 Hz), 70.3, 46.9 (fluorene-C), 27.0, 23.6 (overlapped with the doublet of 23.5 ppm), 23.5, 19.40(d, J_{C-P} = 48.4 Hz), 13.2. ESI MS: m/z: 527.3544 (calcd for M+: 527.3550).

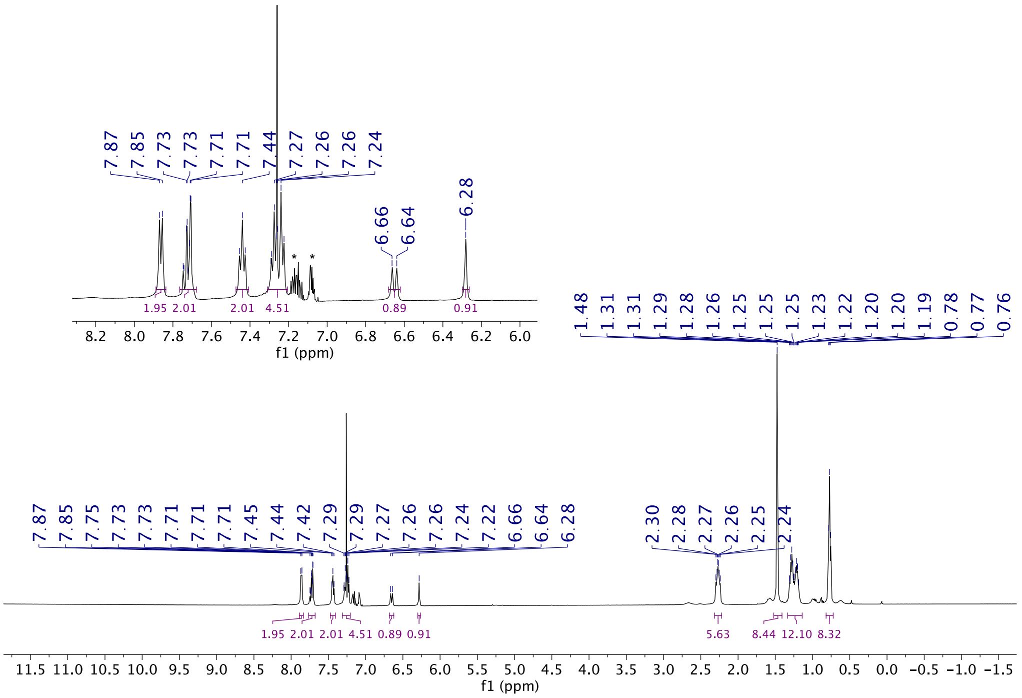

Figure S20: ^{1}H ($CDCl_3$) NMR spectrum of **5** (Asterisk denoted ODFB residue in $CDCl_3$).

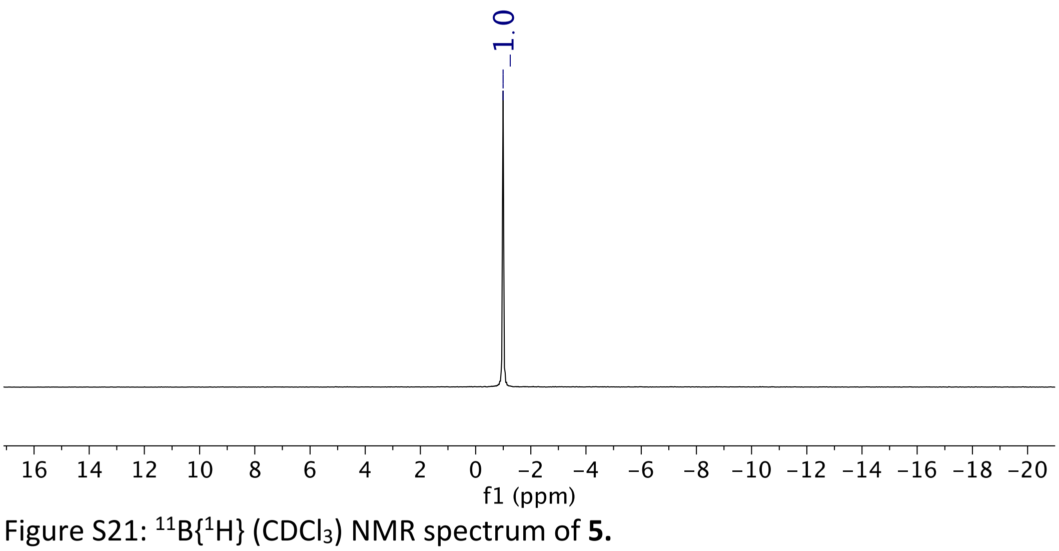

Figure S21: $^{11}B\{^{1}H\}$ ($CDCl_3$) NMR spectrum of **5.**

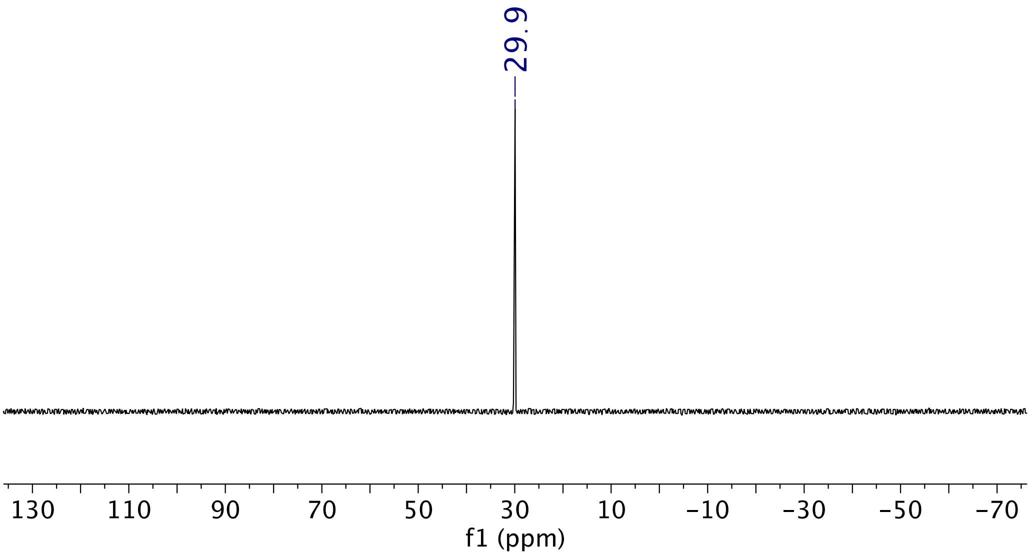

Figure S22: $^{31}P\{^1H\}$ ($CDCl_3$) NMR spectrum of **5**.

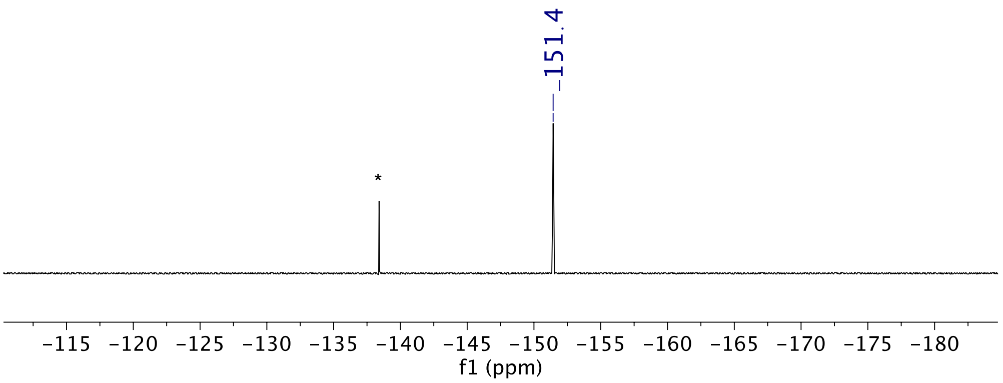

Figure S23: $^{19}F\{^1H\}$ ($CDCl_3$) NMR spectrum of **5.** (Asterisk denoted ODFB residue in $CDCl_3$).

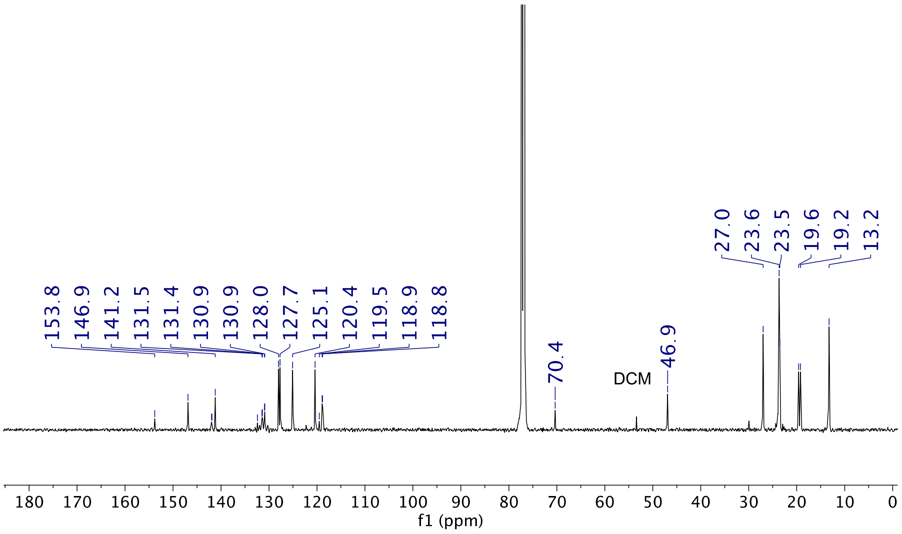

Figure S24: $^{13}C\{^{1}H\}$ ($CDCl_3$) NMR spectrum of **5**.

2.5 Compound 6

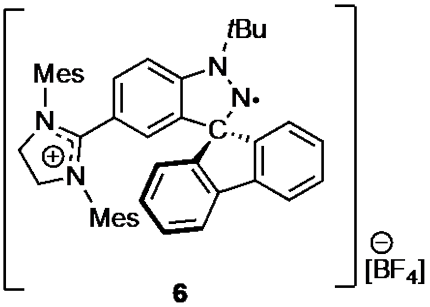

6

To a solution of **1** (41.2 mg, 0.1 mmol) in ODFB (2 mL), a solution of SIMes (30.8 mg, 0.1 mmol) in ODFB was added dropwise. The solution was allowed to stir at ambient temperature for 10 min. All volatiles were removed *in vacuo* and the residue was washed with hexane (2 X 1 mL) and dried under vacuum. Compound **6** was obtained as a purple powder. Yield: 48.3 mg (67% isolated yield). Compound **6** decomposes readily in halogenated solvents. Attempts to observe the product by high-resolution mass spectrometry failed due to the instability of the compound under mass spectrometry conditions, by either ESI or DART methods. In positive mode ESI, $[M-1]^+$ and $[M-2]^+$ were observed.

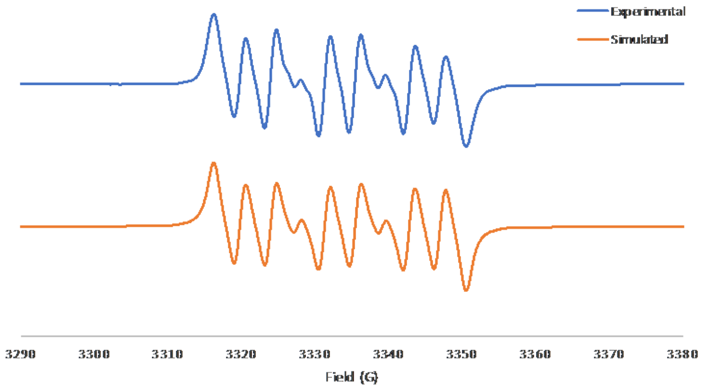

Figure S25: Experimental and simulated EPR spectra of **6**.

Table S1. Experimental and simulated EPR data of **6**.

MF (Par file)	HCF (sim)	G shift	g-factor	Hyperfine couplings (sim)
9.35385	3335.4	-2.097	2.004933387	(^{14}N) 11.50 G, (^{14}N) 4.18 G, (^{1}H) 0.59 G, (^{1}H) 0.41 G, (^{1}H) 0.21 G, (^{14}N) 0.98G, (^{14}N) 0.33 G

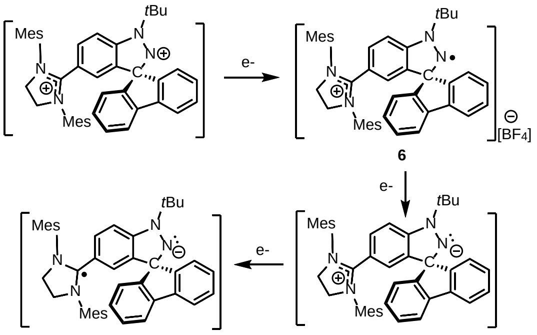

Scheme S1. proposed redox scheme of **6.**

2.6 Compound 7

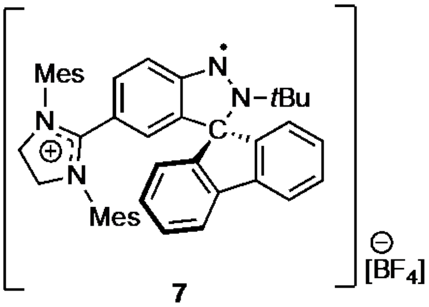

7

To a solution of **2** (41.2 mg, 0.1 mmol) in ODFB (2 mL), a solution of SIMes (30.8 mg, 0.1 mmol) in ODFB was added dropwise. The solution was allowed to stir at ambient temperature for 10 min. All volatiles were removed *in vacuo* and the residue was washed with hexane (2 X 1 mL) and dried under vacuum. Compound **7** was obtained as a red powder. Single crystals suitable for X-ray diffraction were grown from liquid diffusion of pentane into a saturated THF solution at -35 °C. Yield: 57.6 mg (80% isolated yield). Compound **7** decomposes readily in halogenated solvents. Attempts to observe the product by high-resolution mass spectrometry failed due to the instability of the compound under mass spectrometry conditions, by either ESI or DART methods. In positive mode ESI, $[M-1]^+$ and $[M-2]^+$ were observed.

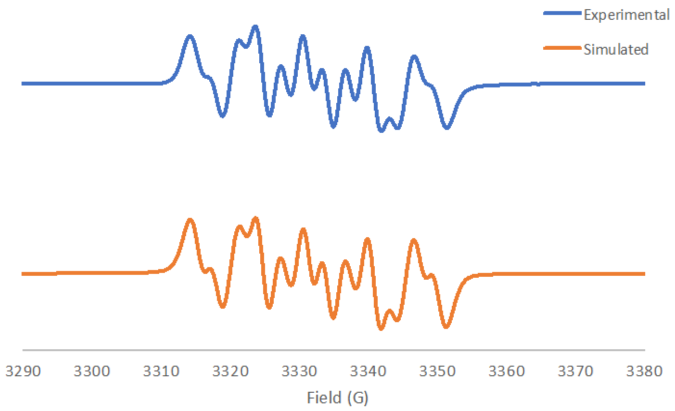

Figure S26: Experimental and simulated EPR spectra of **7**.

Table S2. Experimental and simulated EPR data of **7**.

MF (Par file)	HCF (sim)	G shift	g-factor	Hyperfine couplings (sim)
9.346389	3332	-0.195	2.004234885	(^{14}N) 6.67 G, (^{14}N) 9.386 G, (^{1}H) 0.597 G, (^{1}H) 2.666 G, (^{1}H) 0.532 G, (^{14}N) 0.481 G, (^{14}N) 0.391 G

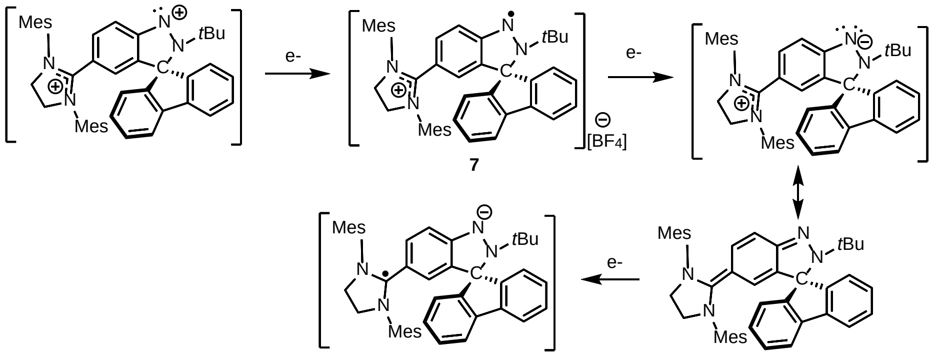

Scheme S2. Proposed redox scheme of **7**.

2.7 Compound 8

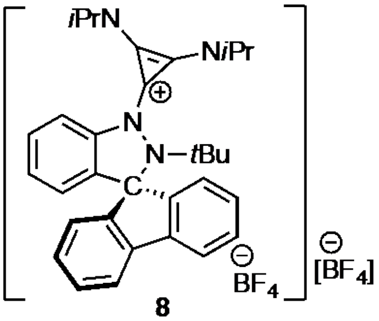

8

To a solution of **2** (41.2 mg, 0.1 mmol) in ODFB (2 mL), a solution of BAC (24.0 mg, 0.1 mmol) in ODFB was added dropwise. The solution was allowed to stir at ambient temperature for 10 min. All volatiles were removed *in vacuo* and the residue was washed with hexane (2 X 1 mL) and dried under vacuum. Compound **8** was isolated as a black powder. Single crystals suitable for X-ray diffraction were grown from liquid diffusion of pentane into a saturated THF solution at -35 °C. Yield: 49.8 mg (78% isolated yield). ^{1}H NMR (500 MHz, $CDCl_3$): δ 7.79 – 7.70 (m, Ar, 2H), 7.42-7.39 (ov, Ar, 4H), 7.31 – 7.20 (ov, Ar, 3H), 6.92 (d, $^{3}J_{H\text{-}H}$ = 8.2 Hz, Ar,

1H), 6.78 (t, $^3J_{H-H}$ = 7.5 Hz, Ar, 1H), 6.14 (d, $^3J_{H-H}$ = 7.6 Hz, Ar, 1H), 4.19 (hept, $^3J_{H-H}$ = 6.8 Hz, *i*Pr, 4H), 1.52 – 1.45 (m, *i*Pr, 25H), 1.02 (s, *t*Bu, 9H). $^{11}B\{^1H\}$ NMR (128 MHz, $CDCl_3$): δ -1.0 (s). $^{19}F\{^1H\}$ NMR (377 MHz, $CDCl_3$): δ -153.0 (br). $^{13}C\{^1H\}$ NMR (126 MHz, $CDCl_3$): δ 145.24, 134.70, 130.92, 129.33, 129.05, 127.80, 124.79, 121.52, 120.37, 115.29, 112.94, 79.81 (fluorene-C), 68.14, 62.10, 29.76, 25.77. Attempts to observe the product by high-resolution mass spectrometry failed due to the instability of the compound under mass spectrometry conditions, by either ESI or DART methods. In positive mode ESI, $[C_{23}H_{21}N_2]^+$ was observed.

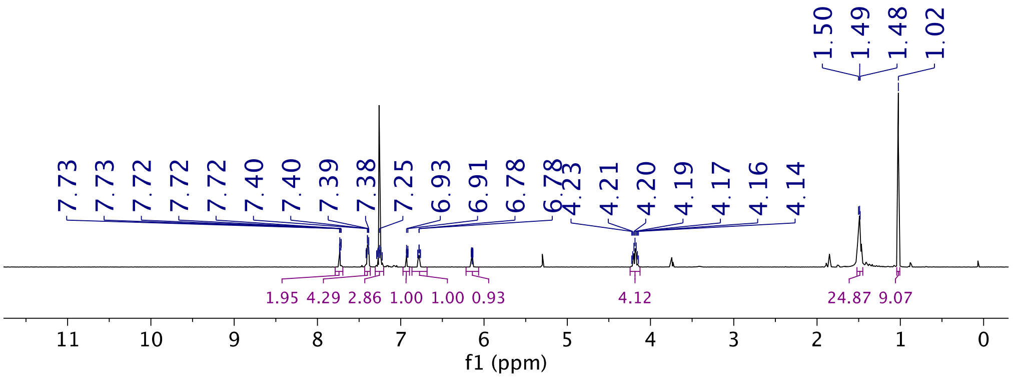

Figure S27: ^1H ($CDCl_3$) NMR spectrum of **8**.

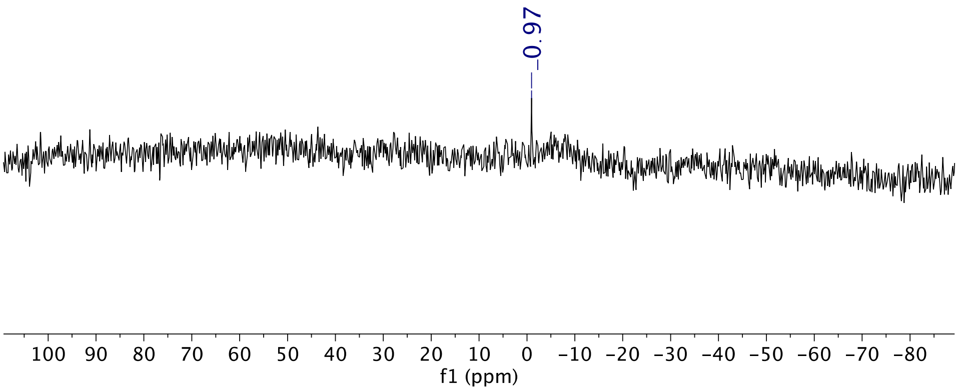

Figure S28: $^{11}B\{^1H\}$ ($CDCl_3$) NMR spectrum of **8.**

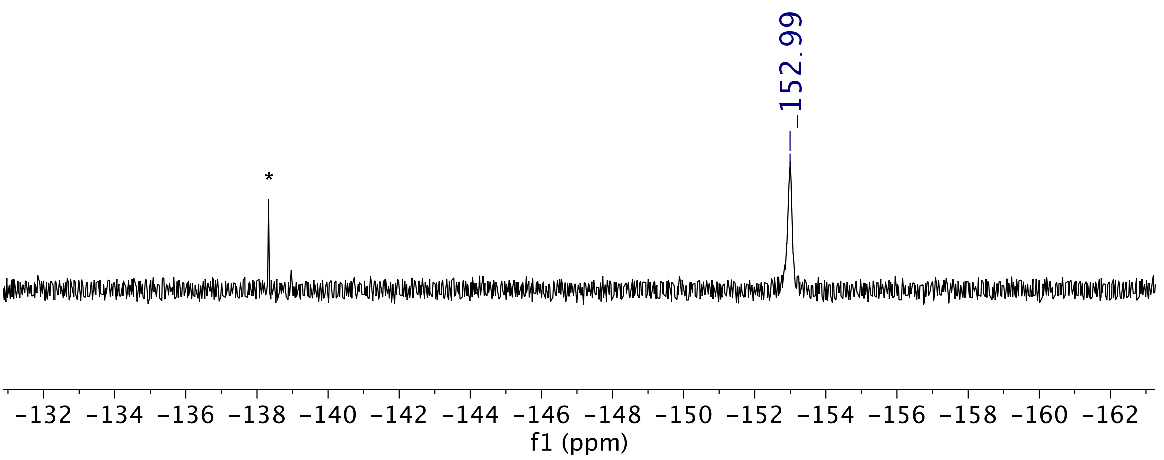

Figure S29: $^{19}F\{^{1}H\}$ ($CDCl_3$) NMR spectrum of **8** (Asterisk denoted ODFB impurity in $CDCl_3$).

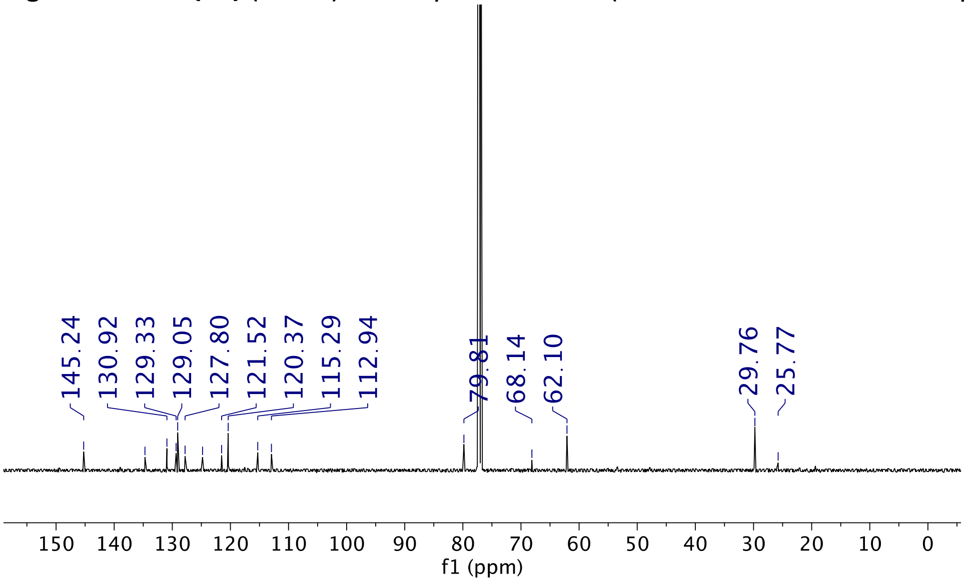

Figure S30: $^{13}C\{^{1}H\}$ ($CDCl_3$) NMR spectrum of **8**.

3. Crystallographic data

X-ray Diffraction Studies

Single crystals were coated with paratone oil, mounted on a cryoloop and frozen under a stream of cold nitrogen. Data were collected on a Bruker Apex2 X-ray diffractometer at 150(2) K for compound **1-3** and **5-7** crystals using graphite monochromated Mo-Kα radiation (0.71073 Å). Data were collected using Bruker APEX-2 software and processed using SHELX and an absorption correction applied using multi-scan within the APEX-2 program. Compound **4** was collected with a Cu microsurce and Bruker CMOS PHOTON II detector gave very weak data. However the structure obtained is of sufficient precision to allow us to identify and confirm the compound synthesised. All structures were solved and refined by

intrinsic phasing within the SHELXTL package.[S4] These data can be obtained free of charge from the Cambridge Crystallographic Data Centre.

S4. (a) G. M. Sheldrick, SADABS, An Empirical Absorption Correction Program for Area Detector Data; University of Göttingen, Göttingen, Germany, 1996; (b) G. M. Sheldrick, SHELXS-97 and SHELXL-97, University of Göttingen, Göttingen, Germany, 1997 and 2008; (c) G. M. Sheldrick, SHELXL-2014, University of Göttingen, Göttingen, German, 2014; (d) O. V. Dolomanov, L. J. Bourhis, R. J. Gildea, J. A. K. Howard and H. Puschmann, OLEX2: a complete structure solution, refinement and analysis program, J. Appl. Cryst., 2009, 42, 339-341; (e) SMART Version 5.628, Bruker AXS Inc., Madison, WI, 2002; (f) SAINT+ Version 6.22a, Bruker AXS Inc., Madison, WI, 2002; (g) SAINT+ Version v7.68A, Bruker AXS Inc., Madison, WI, 2009; (h) SHELXTL NT/2000, Version 6.1, Bruker AXS Inc., Madison, WI, 2002.

Table S3. Crystallographic data and refinement parameters for **1-5**,**7**, and **8**.

	**1**	**2**	**3**
Empirical formula	$C_{25}H_{25}BCl_4F_4N_2$	$C_{23}H_{21}BF_4N_2$	$C_{26}H_{30}BF_4N_2P$
Formula weight	582.08	412.23	488.30
Colour	Red	Orange	Colourless
Crystal system	monoclinic	monoclinic	orthorhombic
Space group	C2/c	$P2_1/n$	$P2_12_12_1$
a/Å	22.3554(13)	11.099(7)	10.895(2)
b/Å	11.5871(6)	16.092(12)	14.037(3)
c/Å	22.7385(19)	11.325(5)	15.791(3)
α/°	90	90	90
β/°	113.902(3)	94.27(2)	90
γ/°	90	90	90
Volume/Å^3	5384.9(6)	2017(2)	2414.9(7)
Z	8	4	4
ρ_{calc}g/cm^3	1.436	1.357	1.343
F(000)	2384.0	856.0	1024.0
T(K)	150(2)	150(2)	150(2)
2θ range for data collection/°	3.918 to 52.04	4.406 to 52.896	4.542 to 54.37
Reflections collected	34565	30168	44163
Independent reflections	5323 [R_{int} = 0.0488, R_{sigma} = 0.0471]	4151 [R_{int} = 0.1154, R_{sigma} = 0.0938]	5341 [R_{int} = 0.1233, R_{sigma} = 0.0884]
Data/restraints/parameters	5323/30/338	4151/0/274	5341/0/313
Goodness-of-fit on F^2	1.050	1.011	1.032
Final R indexes [I>=2σ (I)]	R_1 = 0.0730, wR_2 = 0.1618	R_1 = 0.0597, wR_2 = 0.1102	R_1 = 0.0513, wR_2 = 0.0878
Largest diff. peak/hole / e Å^{-3}	0.92/-0.90	0.48/-0.47	0.25/-0.36

	**4**
Empirical formula	C29.50H34BF4N2P
Formula weight	534.36
Wavelength (Å)	1.54178
Colour	Orange
Crystal system	Monoclinic
Space group	$P2_1/c$

a/Å	14.1333(13)
b/Å	13.8313(13)
c/Å	15.7694(14)
α/°	90
β/°	115.864(6)
γ/°	90
Volume/$Å^3$	2773.9(5)
Z	4
$ρ_{calc}g/cm^3$	1.280
F(000)	1124
T(K)	150(2)
θ range for data collection/°	3.475 to 66.132
Reflections collected	44606
Independent reflections	4795 [R(int) = 0.3444]
Data/restraints/parameters	4795 / 83 / 396
Goodness-of-fit on F^2	1.013
Final R indexes [I>=2σ (I)]	R1 = 0.0988, wR2 = 0.2321
Largest diff. peak/hole / e $Å^{-3}$	0.586 and -0.322 e.$Å^{-3}$

	**5**	**7**	**8**
Empirical formula	$C_{76}H_{100}B_2ClF_8N_4P_2$	$C_{52}H_{63}F_4N_4BO_2$	$C_{42}H_{57}BF_4N_4O$
Formula weight	1340.60	862.87	720.72
Colour	Orange	Red	Black
Crystal system	triclinic	triclinic	orthorhombic
Space group	P-1	P-1	$Pna2_1$
a/Å	13.9515(18)	13.236(3)	15.1948(7)
b/Å	16.383(2)	13.457(3)	13.4053(7)
c/Å	18.022(2)	15.124(3)	19.3295(8)
α/°	85.244(3)	91.958(5)	90
β/°	79.244(3)	111.437(6)	90
γ/°	67.835(3)	110.427(5)	90
Volume/$Å^3$	3747.4(8)	2308.9(7)	3937.2(3)
Z	2	2	4
$ρ_{calc}g/cm^3$	1.188	1.241	1.216
F(000)	1426.0	920.0	1544.0
T(K)	150(2)	150(2)	150(2)
2θ range for data collection/°	2.684 to 50.914	3.61 to 52.852	3.698 to 51.39
Reflections collected	77967	41640	59201
Independent reflections	13742 [R_{int} = 0.1466, R_{sigma} = 0.2244]	9480 [R_{int} = 0.0559, R_{sigma} = 0.0768]	7477 [R_{int} = 0.1051, R_{sigma} = 0.0821]
Data/restraints/parameters	13742/151/819	9480/365/691	7477/31/492
Goodness-of-fit on F^2	0.905	1.037	1.039
Final R indexes [I>=2σ (I)]	R_1 = 0.0855, wR_2 = 0.2219	R_1 = 0.0680, wR_2 = 0.1802	R_1 = 0.0607, wR_2 = 0.1308
Largest diff. peak/hole / e $Å^{-3}$	0.71/-0.75	0.36/-0.52	0.34/-0.29

4. Kinetic data

Compound **1** dissolves in chloroform, CH_2Cl_2, THF, and ODFB. In order to be able to monitor the reactions by ^{1}H NMR spectroscopy, we chose affordable $CDCl_3$ as solvent.

Compound **1** was dissolved in $CDCl_3$ in a sealed NMR tube. The concentrations of **1** and **2** were monitored by ^{1}H NMR spectroscopy at 50 °C (323 K) over 175 mins. 1,2-Dichloroethane was used as the internal standard.

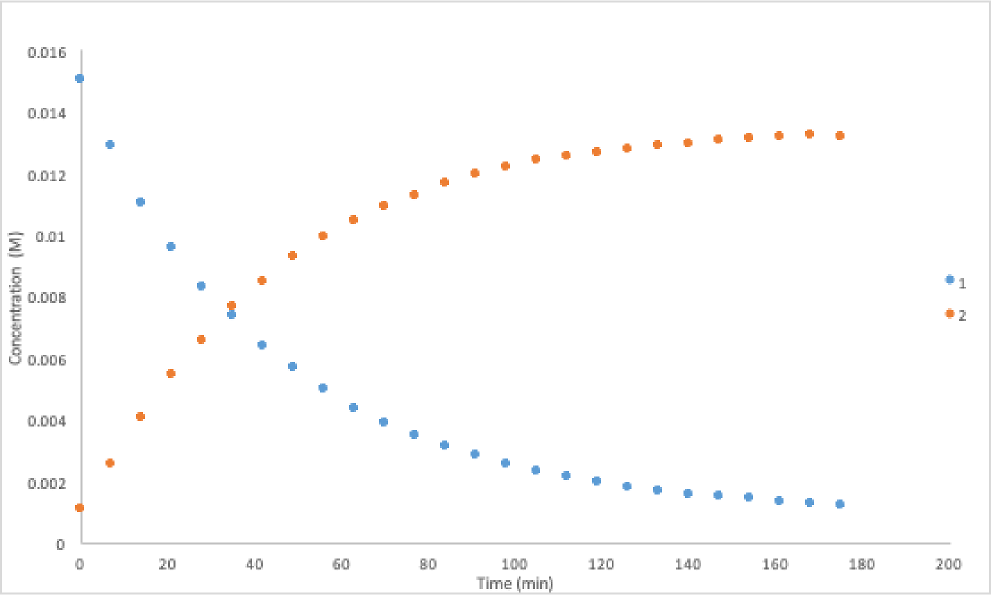

Figure S31: Concentrations of compound **1** and **2** at 323 K over 175 mins.

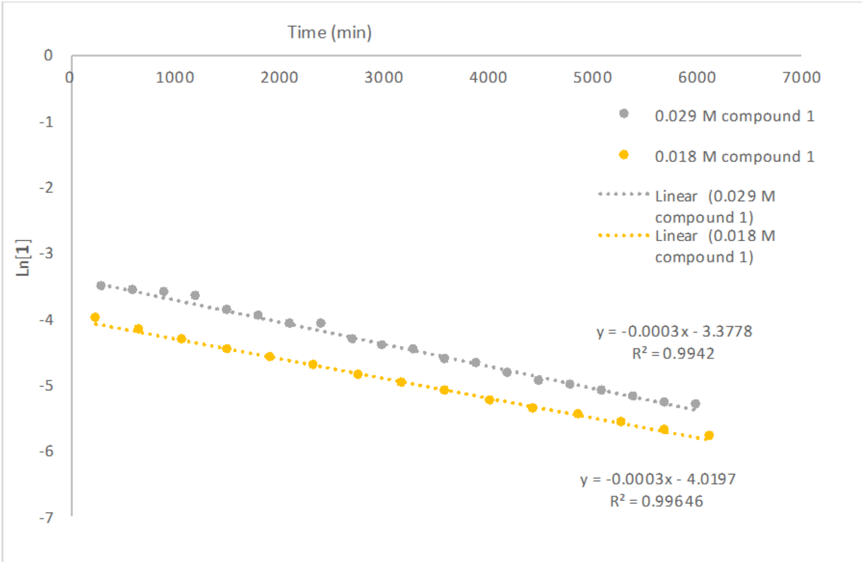

Figure S32: Kinetic data of compound **1** at two concentrations over 100 mins at 323 K.

The initial experiment run to full consumption showed the kinetic profile of the formation of **2** to be linear until ca. 33 minutes (Figure S31). As such, only aliquots taken in the first 33 minutes (n = 5) were analyzed for all parallel experiments from 303 to 323 K. **1** (ca. 4.4 mg) was dissolved in 0.6 mL of $CDCl_3$ in a sealed NMR tube. The concentration of **2** was

monitored by ^{1}H NMR spectroscopy. 1,2-Dichloroethane (0.0399 M) was used as the internal standard.

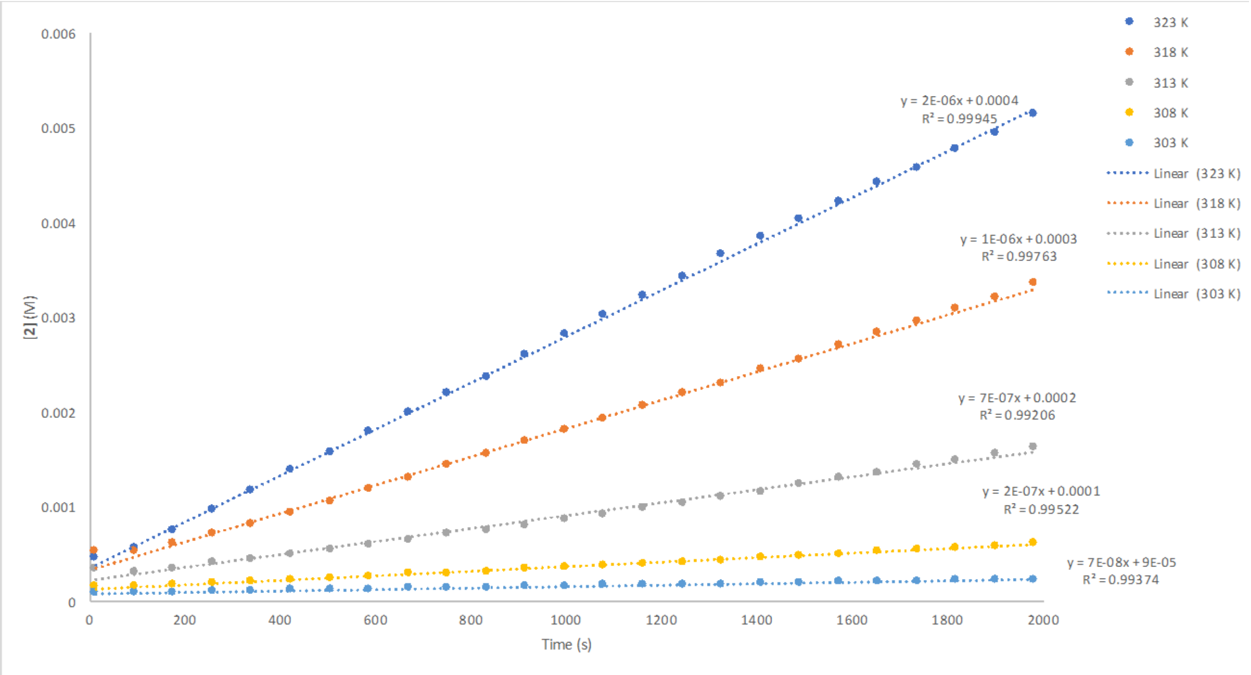

Figure S33: Concentrations of compound **2** under various temperatures.

Table S4. Rate constants derived from kinetic experiments (Figure S33).

T (K)	1/T (1/K)	k(Ms^{-1})	ln(k/T)
323	0.00309	2.44E-06	-18.70
318	0.00314	1.49E-06	-19.17
313	0.00319	6.84E-07	-19.94
308	0.00324	2.37E-07	-20.98
303	0.00330	7.42E-08	-22.12

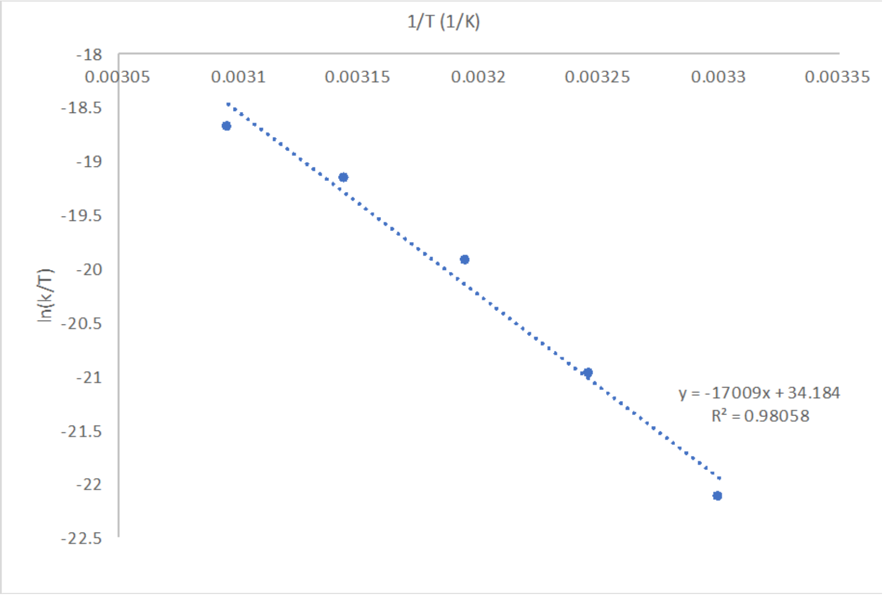

Figure S34: Eyring plot of data from table S4.

Standard errors were calculated using regression analysis in the Microsoft Excel program. The activation parameters for the reaction were found to be (at 95% confident level): $\Delta H^{\ddagger}$ = 34 ± 3 kcal/mol, $\Delta S^{\ddagger}$ = 21 ± 9 cal/(mol·K) and an associated $\Delta G^{\ddagger}_{298\ K}$ = 28 ± 5 kcal/mol.

5. Observation of the radical 9

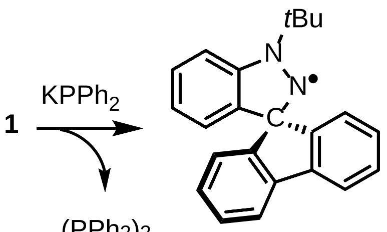

9

A THF solution of $KPPh_2$ (0.1 mL, 0.5 M) was slowly added to a solution of **1** (21.8 mg, 0.053 mmol) while stirring. The solution immediately turned dark green and was stirred for an additional 15 minutes until submitted to EPR analysis. Formation of spiro[fluorene-9,3'-indazole] was confirmed by X-ray crystallography.

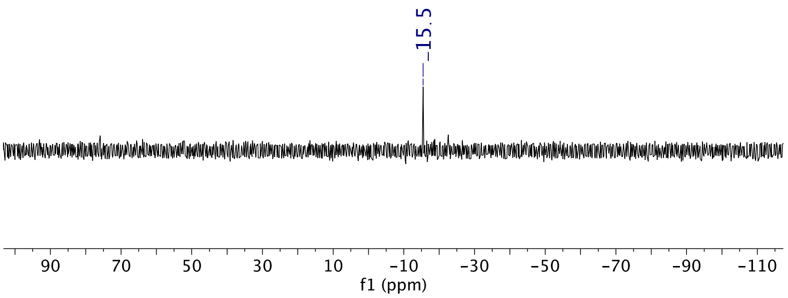

Figure S35: $^{31}P\{^1H\}$ NMR spectrum of reaction mixture in THF.

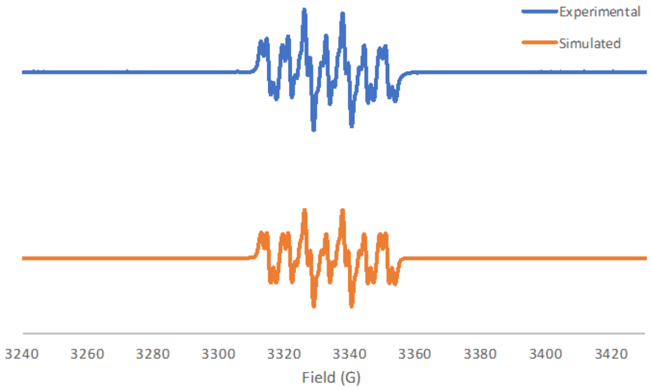

Figure S36: Experimental and simulated EPR spectra of **9** in THF.

Table S5. Experimental and simulated EPR data of radical **9.**

MF (Par file)	HCF (sim)	G shift	g-factor	Hyperfine couplings (sim)
9.348171	3334.19	-1.032	2.0038033	(^{14}N) 11.603 G, (^{14}N) 6.534 G, (^{1}H) 1.673, (^{1}H) 1.753 G, (^{1}H) 0.489 G, (^{1}H) 0.307 G,

6. Observation of the radical 10

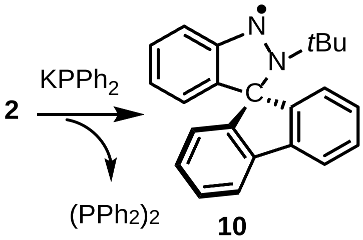

10

A THF solution of $KPPh_2$ (0.1 mL, 0.5 M) was slowly added to a solution of **2** (21.8 mg, 0.053 mmol) while stirring. The solution immediately turned dark green and was stirred for an additional 15 minutes until submitted to EPR analysis. Formation of spiro[fluorene-9,3'-indazole] was confirmed by X-ray crystallography.

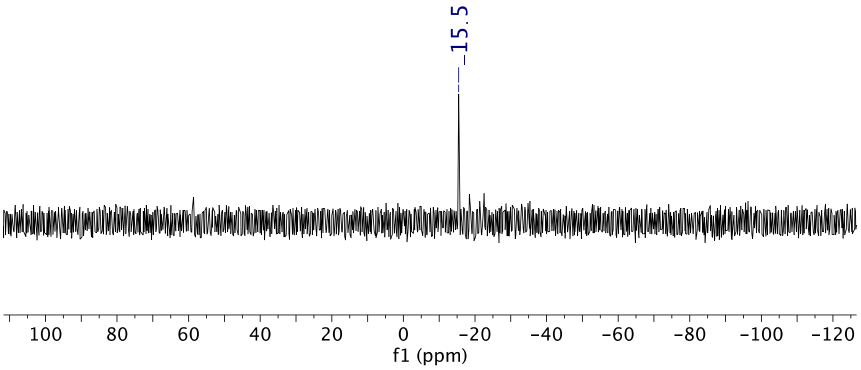

Figure S37: $^{31}P\{^{1}H\}$ NMR spectrum of reaction mixture in THF.

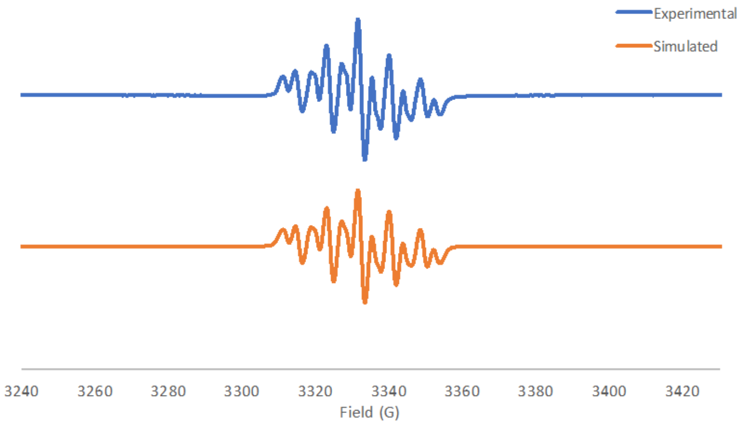

Figure S38: Experimental and simulated EPR spectra of **10** in THF.

Table S6. Experimental and simulated EPR data of radical **10.**

MF (Par file)	HCF (sim)	G shift	g-factor	Hyperfine couplings (sim)
9.345129	3334.6	-2.285	2.00365799	(^{14}N) 8.481 G, (^{14}N) 8.486 G, (^{1}H) 3.115, (^{1}H) 3.482 G, (^{1}H) 0.276 G, (^{1}H) 0.2 G,

7. Observation of H_2 in reaction of 2 and SIMes

2 (10.0 mg, 0. 024mmol) was carefully transferred to a J-Young tube. A solution of SIMes (12.8 mg, 0.024 mmol) in C_6D_6 (0.5 mL) was added. Due to the poor solubility of compound **2** in C_6D_6, the reaction preceded very slowly and the reaction was monitored at room temperature by ^{1}H NMR spectroscopy.

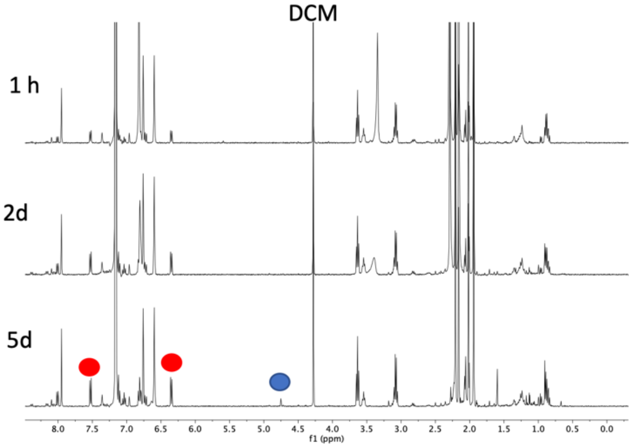

Figure S39: ^{1}H NMR spectra of reaction of **2** and SIMes at room temperature over time. Blue dot indicates the formation of H_2 and red dots indicate unknown organic species.

8. Electrochemistry

Cyclic voltammetry experiments performed using BASi-Epsilon RDE-2 model. A standard three-electrode cell configuration was employed using a glassy graphite working electrode, a platinum wire counter electrode, and a silver wire serving as a reference electrode. Formal redox potentials were referenced to the ferrocenium/ferrocene redox couple. [*n*Bu4N][BF4] (0.085 M) was used as supporting electrolyte. CVs of compound **1** and **2** are measured with 2 mM analytes while CVs of compound **6** and **7** are measured with 1 mM analytes. All measurements were performed at room temperature.

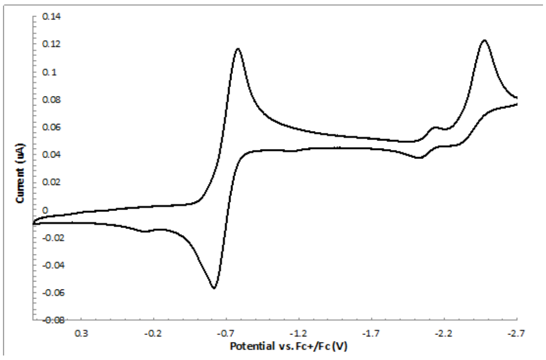

Figure S40: Cyclic voltammogram of **1** in ODFB (scan rate: 200mV/s).

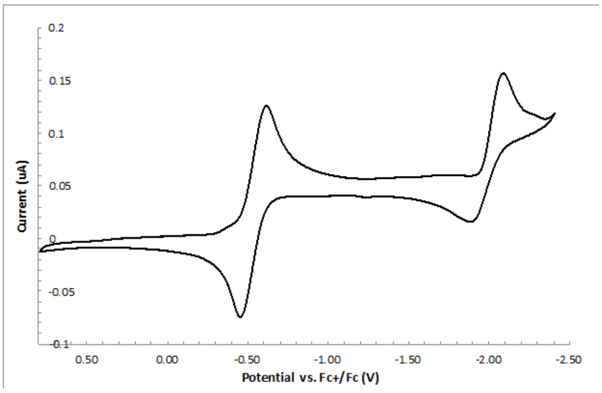

Figure S41: Cyclic voltammogram of **2** in ODFB (scan rate: 200mV/s).

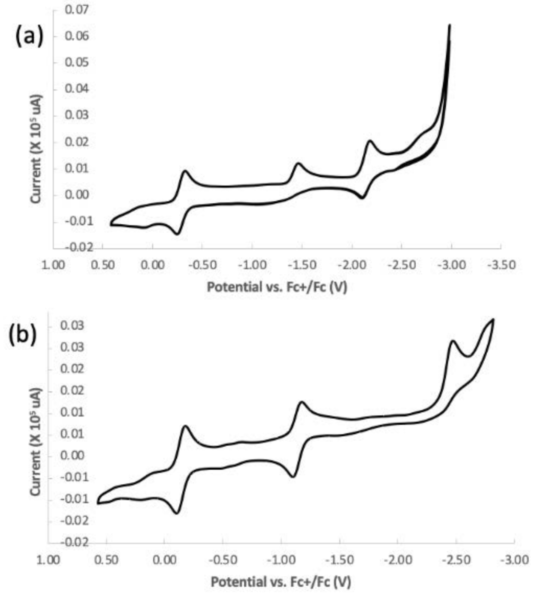

Figure S42. Cyclic voltammograms of (a) **6** and (b) **7** (scan rate: 100 mV/s)

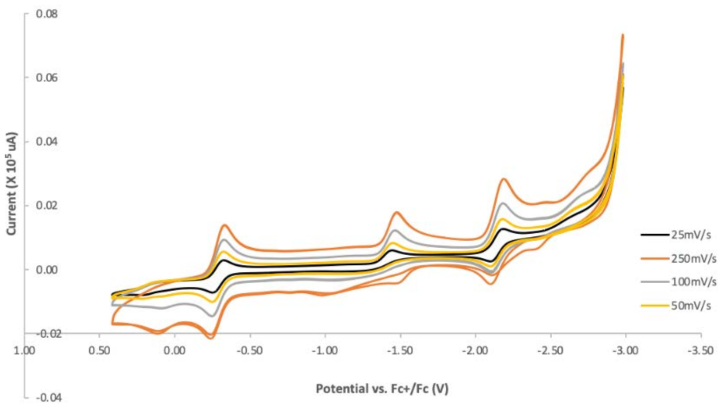

Figure S43. Cyclic voltammograms of **6** at various scan rates.

9. Computational details

The quantum chemical DFT calculations have been performed with the TURBOMOLE 7.3 suite of programs[1] The structures are fully optimized at the TPSS-D3/def2-TZVP + COSMO($CHCl_3$) level of theory, which combines the TPSS meta-GGA density functional[2] with the BJ-damped DFT-D3 dispersion correction[3, 4] and the def2-TZVP basis set,[5, 6] using the Conductor-like Screening Model (COSMO) continuum solvation model[7] for $CHCl_3$ solvent (dielectric constant ε = 4.8 and solvent diameter R_{solv} = 3.17 Å). The density-fitting RI-J approach[5, 8, 9] is used to accelerate the geometry optimization and numerical harmonic frequency calculations[10] in solution. The optimized structures are characterized by frequency analysis to identify the nature of located stationary points (no imaginary frequency for true minima and only one imaginary frequency for transition state) and to provide thermal corrections (at 298.15 K and 1 atm) according to the modified ideal gas–rigid rotor–harmonic oscillator model.[11] This choice of dispersion-corrected meta-GGA functional makes the efficient exploration of all potential reaction paths possible.

The final solvation free energies in $CHCl_3$ are computed with the COSMO-RS solvation model[12] (parameter file: BP_TZVP_C30_1601.ctd) using the COSMOtherm program package[13 on the above TPSS-D3 optimized structures, and corrected by +1.89 kcal·mol^{-1} to account for higher reference solute concentration of 1 mol·L^{-1} usually used in solution. To check the effects of the chosen DFT functional on the reaction energies and barriers, single-point calculations at the meta-GGA TPSS-D3[2] and hybrid-meta-GGA PW6B95-D3[14] levels are performed using a larger def2-QZVP basis set.[6, 15] The final reaction Gibbs free energies (ΔG) are determined from the electronic single-point energies plus TPSS-D3 thermal corrections and COSMO-RS solvation free energies. The computed reaction free energies from both DFT functionals are in good mutual agreement of -0.9 $\pm$ 1.9 for reaction free energies (average and standard deviations, see Table S1 below) despite 3.5 $\pm$ 2.2 kcal/mol higher barriers at PW6B95-D3 level as expected. In our discussion, higher-level PW6B95-D3 Gibbs free energies (in kcal/mol, at 298.15 K and 1 mol/L concentration) will be used in our discussion unless specified otherwise.

Table S7. TPSS-D3/def2-TZVP + COSMO computed imaginary frequency (ImF), zero-point energies (ZPE), gas-phase enthalpic (Hc) and Gibbs free-energy (Gc) corrections; the COSMO-RS computed solvation enthalpic (Hsol) and Gibbs free-energy (Gsol) corrections in $CHCl_3$ solution; TPSS-D3/def2-QZVP and PW6B95-D3/def2-QZVP single-point energies (TPSS-D3 and PW6B95-D3); the total PW6B95-D3 free energies G_P; the relative electronic energies (ΔE_T and ΔE_P) and Gibbs free-energies (ΔG_T and ΔG_P) at the TPSS-D3 and PW6B95-D3 levels.

Reactions	ImF cm^{-1}	ZPE kcal /mol	Hc kcal /mol	Gc kcal /mol	Hsol kcal /mol	Gsol kcal /mol	TPSS-D3 Eh	PW6B95-D3 Eh	G_P Eh	ΔE_T kcal /mol	ΔE_P kcal /mol	*ΔG_P kcal /mol*	ΔG_T kcal /mol
*Separated ion pairs of cations **1$^+$**, **2$^+$** and PMe_3H^+ and anion BF_4^- in $CHCl_3$ solution*													
**1$^+$** + BF_4^-	0	246.10	262.31	202.45	-127.69	-104.12	-1423.81046	-1425.32366	-1425.16094	0.00	0.00	*0.00*	0.00
**1**	0	247.08	263.82	214.95	-59.97	-43.58	-1423.91236	-1425.42660	-1425.15049	-63.94	-64.60	*6.55*	7.21
**2$^+$** + BF_4^-	0	245.30	261.80	201.42	-126.64	-103.35	-1423.81730	-1425.33086	-1425.16857	0.00	0.00	*0.00*	0.00
**2**	0	246.03	263.09	213.84	-50.18	-35.08	-1423.93269	-1425.44792	-1425.16004	-72.41	-73.46	*5.35*	6.40
PMe_3H^+ + BF_4^-	0	87.68	95.37	54.63	-128.81	-108.16	-886.38863	-887.20525	-887.28452	0.00	0.00	*0.00*	0.00
$PMe_3H^+BF_4^-$	0	87.87	95.87	64.82	-35.57	-24.54	-886.53170	-887.34933	-887.28213	-89.78	-90.41	*1.50*	2.13
*Facile N..P addition reaction of **1$^+$** and phosphine PMe_3*													
**1$^+$** + PMe_3	0	307.79	325.11	262.71	-62.56	-50.71	-1460.27076	-1461.74167	-1461.39780	0.00	0.00	*0.00*	0.00
**TS1$^+$**	110i	306.06	324.17	273.23	-53.10	-45.95	-1460.27907	-1461.74306	-1461.37785	-5.22	-0.87	*12.52*	8.17
**3$^+$**	0	308.04	325.55	276.27	-54.87	-47.69	-1460.30758	-1461.78212	-1461.41484	-23.11	-25.38	*-10.70*	-8.42
**TS1a$^+$**	204i	305.29	323.77	271.71	-56.49	-48.23	-1460.26672	-1461.72911	-1461.36997	2.53	7.88	*17.46*	12.11
**3a$^+$**	0	308.67	326.09	276.80	-60.35	-51.29	-1460.27086	-1461.73841	-1461.37602	-0.07	2.05	*13.67*	11.56
**3b$^+$**	0	309.71	326.99	277.96	-58.82	-50.64	-1460.31162	-1461.78881	-1461.42354	-21.35	-25.06	*-11.37*	-7.66
..tBu$^+$ transfer to PMe_3 is prevented by a high barrier													
**TS1m$^+$**	263i	303.75	323.01	268.87	-54.72	-46.72	-1460.24217	-1461.70359	-1461.34656	17.94	23.89	*32.15*	26.20
**1m** + *t*BuPMe_3^+	0	307.34	324.60	261.42	-75.08	-62.78	-1460.28661	-1461.76112	-1461.43855	-9.95	-12.20	*-25.57*	-23.31
*..PMe_3 aided H$^+$ shift within **3a$^+$** is exergonic but prevented by a high barrier via **TS3p$^+$***													
**1$^+$** + 2PMe_3	0	378.10	399.92	315.70	-72.79	-55.80	-1921.50530	-1923.36507	-1922.94185	0.00	0.00	*0.00*	0.00
**3a$^+$** + PMe_3	0	378.97	400.90	329.79	-70.58	-56.37	-1921.50541	-1923.36181	-1922.92007	-0.07	2.05	*13.67*	11.56
**TS3p$^+$**	972i	372.97	396.57	334.61	-65.55	-54.31	-1921.48736	-1923.33619	-1922.88650	11.26	18.12	*34.73*	27.86
**3p** + PMe_3H^+	0	380.14	401.56	331.43	-96.52	-79.53	-1921.44698	-1923.30247	-1922.89502	36.60	39.28	*29.39*	26.70
**TS3b$^+$**	0	374.33	398.33	335.41	-57.65	-48.58	-1921.52115	-1923.37588	-1922.91577	-9.95	-6.78	*16.36*	13.20
**3b$^+$** + PMe_3	0	380.01	401.79	330.95	-69.05	-55.72	-1921.54617	-1923.41221	-1922.96759	-25.64	-29.58	*-16.15*	-12.22

*Isomerization from cation **1$^+$** to **2$^+$** via 1,2-tBu shift*

**1**$^+$	0	237.49	250.30	209.72	-52.33	-45.62	-999.03621	-1000.11827	-999.85375	0.00	0.00	*0.00*	0.00
**TS1b**$^+$	111i	231.49	245.76	201.56	-60.64	-50.64	-998.97819	-1000.06031	-999.81679	36.41	36.37	*23.19*	23.23
**2**$^+$	0	236.68	249.79	208.69	-51.27	-44.86	-999.04305	-1000.12547	-999.86137	-4.29	-4.52	*-4.79*	-4.56
**1m** + *t*Bu$^+$	0	231.88	245.52	190.36	-79.35	-67.79	-998.94025	-1000.02450	-999.82315	60.22	58.84	*19.20*	20.58
*Single electron transfer (SET) reduction of cations **1**$^+$ and **2**$^+$*													
**1**$^+$	0	237.48	250.30	209.72	-52.33	-45.62	-999.03621	-1000.11827	-999.85375	0.00	0.00	*0.00*	0.00
**1r** (or **9**$^{\cdot}$)	0	238.93	251.13	211.86	-26.91	-18.79	-999.23361	-1000.32114	-1000.01045	-123.87	-127.30	*-98.33*	-94.90
*...**2**$^+$ is 3.6 kcal/mol more oxidative*													
**2**$^+$	0	236.68	249.79	208.69	-51.27	-44.86	-999.04305	-1000.12547	-999.86137	0.00	0.00	*0.00*	0.00
**2r** (or **10**$^{\cdot}$)	0	236.47	249.31	208.80	-24.29	-17.25	-999.24441	-1000.33206	-1000.02379	-126.35	-129.63	*-101.92*	-98.64
**2**$^-$	0	233.54	246.68	205.65	-78.99	-60.70	-999.27713	-1000.36462	-1000.13061	-146.89	-150.07	*-168.95*	-165.77
*...Using dimeric $(KPPh_2)_2$ as SET reductant: only first reduction to radicals **1r** and **2r** is efficient.*													
$(KPPh_2)_2$	0	225.47	243.61	190.65	-56.14	-37.92	-2810.03332	-2812.40616	-2812.15975	0.00	0.00	*0.00*	0.00
$(KPPh_2)_2^+$	0	227.42	244.97	193.75	-74.52	-63.57	-2809.85337	-2812.22062	-2812.01015	112.92	116.43	*93.87*	90.36
$2KPPh_2$	0	224.68	242.11	175.36	-90.13	-61.42	-2809.97436	-2812.34401	-2812.15642	37.00	39.00	*2.09*	0.09
…$(PPh_2)_2$ formation…													
$(KPPh_2)_2$ + **1**$^+$	0	462.96	493.91	400.37	-108.47	-83.54	-3809.06953	-3812.52443	-3812.01350	0.00	0.00	*0.00*	0.00
$(KPPh_2)_2^+$ + **1r**	0	466.35	496.10	405.60	-101.44	-82.35	-3809.08699	-3812.54176	-3812.02061	-10.95	-10.88	*-4.46*	-4.54
$(KPPh_2)_2^+$ + BF_4^-	0	236.04	256.97	186.47	-149.89	-122.06	-3234.62762	-3237.42601	-3237.31734	0.00	0.00	*0.00*	0.00
KBF_4 + $K(PPh_2)_2$	0	236.82	257.50	185.92	-80.03	-58.89	-3234.73902	-3237.53604	-3237.32758	-69.91	-69.04	*-6.42*	-7.29
$K(PPh_2)_2$ + **1**$^+$	0	465.15	493.94	405.55	-100.91	-81.65	-3209.04525	-3212.08500	-3211.56280	0.00	0.00	*0.00*	0.00
$K(PPh_2)_2^+$ + **1r**	0	465.90	494.57	406.25	-93.08	-76.34	-3209.11221	-3212.16037	-3211.62860	-42.02	-47.30	*-41.29*	-36.01
$K(PPh_2)_2^+$ + BF_4^-	0	235.59	255.44	187.12	-141.53	-116.05	-2634.65285	-2637.04462	-2636.92534	0.00	0.00	*0.00*	0.00
KBF_4 + $(PPh_2)_2$	0	235.40	255.11	185.95	-59.00	-42.85	-2634.77855	-2637.17220	-2636.93813	-78.88	-80.06	*-8.03*	-6.85
$(KPPh_2)_2$ + **2**$^+$	0	462.15	493.40	399.34	-107.41	-82.78	-3809.07637	-3812.53163	-3812.02112	0.00	0.00	*0.00*	0.00
$(KPPh_2)_2^+$ + **2r**	0	463.90	494.28	402.55	-98.81	-80.81	-3809.09779	-3812.55268	-3812.03394	-13.44	-13.21	*-8.05*	-8.27
…Phosphine PMe_3 is not reductive enough for SET reaction													
PMe_3 + **1**$^+$	0	307.79	325.11	262.71	-62.56	-50.71	-1460.27076	-1461.74167	-1461.39780	0.00	0.00	*0.00*	0.00
PMe_3^+ + **1r**	0	311.30	327.54	267.28	-80.79	-68.75	-1460.18892	-1461.66117	-1461.33876	51.35	50.51	*37.05*	37.89
***2**$^+$ addition with phosphine PMe_3: thermodynamic control for selective para-C site*													
Reversible P…para-C and P…N additions													

**2**$^+$ + PMe_3	0	306.99	324.60	261.68	-61.50	-49.94	-1460.27760	-1461.74887	-1461.40542	0.00	0.00	*0.00*	0.00
**TS2**$^+$	182i	305.23	323.75	271.65	-54.07	-46.67	-1460.28335	-1461.74713	-1461.38559	-3.61	1.09	*12.45*	7.74
**2p**$^+$	0	307.59	325.38	275.36	-58.60	-50.62	-1460.29131	-1461.76342	-1461.40226	-8.60	-9.13	*1.98*	2.51
**TS4a**$^+$	159i	305.47	323.77	272.36	-54.25	-47.06	-1460.27711	-1461.74453	-1461.38248	0.31	2.72	*14.40*	11.98
**4a**$^+$	0	307.47	325.26	275.24	-56.09	-48.90	-1460.29468	-1461.77098	-1461.40727	-10.72	-13.87	*-1.16*	2.00
**4b**$^+$	0	308.96	326.37	277.18	-58.48	-50.79	-1460.30679	-1461.78481	-1461.42102	-18.32	-22.55	*-9.79*	-5.55
*..tBu$^+$ transfer from cation **2**$^+$ to PMe_3 is prevented by a high barrier*													
**2**$^+$ + PMe_3	0	306.99	324.60	261.68	-61.50	-49.94	-1460.27760	-1461.74887	-1461.40542	0.00	0.00	*0.00*	0.00
**TS2m**$^+$	284i	303.23	322.71	267.64	-53.35	-45.68	-1460.24659	-1461.70956	-1461.35283	19.46	24.67	*33.00*	27.79
**1m** + *t*BuPMe_3^+	0	307.34	324.60	261.42	-75.08	-62.78	-1460.28661	-1461.76112	-1461.43855	-5.65	-7.68	*-20.79*	-18.75
*..Facile PMe_3-aided H-shift within **2p**$^+$ as rate-limiting step via **TS4p**$^+$*													
**2**$^+$ + 2PMe_3	0	377.29	399.41	314.67	-71.73	-55.03	-1921.51214	-1923.37227	-1922.94948	0.00	0.00	*0.00*	0.00
**TS4p**$^+$	988i	372.79	396.52	334.39	-64.55	-54.19	-1921.51269	-1923.36712	-1922.91759	-0.34	3.23	*20.01*	16.44
**4p** + PMe_3H^+	0	379.84	401.35	331.10	-95.66	-78.95	-1921.47616	-1923.33664	-1922.92878	22.58	22.36	*12.98*	13.21
**4p**.$HPMe_3^+$	0	378.54	400.67	342.42	-63.47	-53.08	-1921.53800	-1923.39628	-1922.93216	-16.22	-15.06	*10.87*	9.70
**TS4b**$^+$	36i	374.21	397.43	336.35	-62.94	-53.03	-1921.53356	-1923.39094	-1922.93644	-13.44	-11.71	*8.18*	6.46
**4b**$^+$ + PMe_3	0	379.26	401.17	330.17	-68.71	-55.88	-1921.54133	-1923.40821	-1922.96508	-18.32	-22.55	*-9.79*	-5.55
**TS4c**$^+$	836i	373.81	397.10	336.04	-61.22	-51.16	-1921.53771	-1923.39447	-1922.93748	-16.04	-13.93	*7.53*	5.42
**4c**$^+$ + PMe_3	0	380.36	402.01	331.47	-68.99	-54.53	-1921.53184	-1923.39388	-1922.94653	-12.36	-13.56	*1.85*	3.05
*..further C-N ring-openning (**TS4d**$^+$) followed by NH..C proton transfer (**TS4e**$^+$) and aryl rotation (**TS4**$^+$)*													
**2**$^+$ + PMe_3	0	306.99	324.60	261.68	-61.50	-49.94	-1460.27760	-1461.74887	-1461.40542	0.00	0.00	*0.00*	0.00
**4c**$^+$	0	310.05	327.20	278.48	-58.76	-49.44	-1460.29729	-1461.77048	-1461.40248	-12.36	-13.56	*1.85*	3.05
**TS4d**$^+$	140i	306.04	324.25	272.79	-58.47	-49.59	-1460.29163	-1461.75657	-1461.39786	-8.80	-4.83	*4.75*	0.77
**4d**$^+$	0	310.07	327.24	278.43	-59.22	-51.00	-1460.29976	-1461.76578	-1461.40035	-13.90	-10.61	*3.18*	-0.11
**TS4e**$^+$	1367i	302.39	320.96	268.62	-60.57	-52.25	-1460.27567	-1461.73935	-1461.39153	1.21	5.97	*8.72*	3.96
**4e**$^+$	0	309.89	327.19	277.68	-61.40	-52.67	-1460.30393	-1461.77890	-1461.41731	-16.52	-18.84	*-7.46*	-5.14
**TS4**$^+$	65i	305.95	323.52	273.55	-61.88	-52.91	-1460.28412	-1461.75682	-1461.40219	-4.09	-4.99	*2.03*	2.92
**4**$^+$	0	307.64	325.67	274.91	-63.68	-54.70	-1460.30198	-1461.77682	-1461.42289	-15.30	-17.54	*-10.96*	-8.72

Table S8. The TPSS-D3/def2-TZVP + COSMO optimized atomic Cartesian coordinates (in Å) in $CHCl_3$ solution. Each structure is labeled by the specific name (See also **Table S7**), followed by the number of atoms, the total energy (in hartrees), and the detailed atomic coordinates (in double-column text list).

1m *t*Bu$^+$ removed from cation **1**$^+$

33

Energy = -841.2474913933

C	2.6415312	-0.6081287	3.1341054
C	1.3922718	-0.0773088	3.4755209
C	2.9018461	-1.0537436	1.8354006
H	3.4187128	-0.6756161	3.8900611
C	0.3744209	0.0199100	2.5193826
H	1.2106705	0.2620305	4.4910325
C	1.8896822	-0.9596017	0.8784139
H	3.8744945	-1.4652266	1.5805004
C	0.6348333	-0.4246223	1.2310558
H	-0.5948717	0.4326269	2.7852334
C	1.8550977	-1.3454266	-0.5364139
C	-0.2904465	-0.4174363	0.0233845
C	0.5787166	-1.0486798	-1.0540048
C	2.8237646	-1.9246649	-1.3581493
C	-1.6601065	-0.9873770	0.2131614
N	-0.6004785	1.0392881	-0.3671461
C	0.2540399	-1.3173561	-2.3759191
C	2.4986967	-2.1976666	-2.6895779
H	3.8124485	-2.1607706	-0.9746102
C	-2.5603696	0.0501835	-0.0488516
C	-2.1278729	-2.2441247	0.5684558
N	-1.8429314	1.2363244	-0.3909308
C	1.2283277	-1.8991809	-3.1955465
H	-0.7316935	-1.0854651	-2.7697723
H	3.2414745	-2.6490148	-3.3412001
C	-3.9398862	-0.1121553	0.0291840
C	-3.5146218	-2.4267811	0.6521978
H	-1.4482640	-3.0651044	0.7763780
H	0.9960657	-2.1211860	-4.2329385
C	-4.4075670	-1.3788908	0.3872608
H	-4.6163739	0.7105315	-0.1794338
H	-3.9072934	-3.4011084	0.9281532
H	-5.4760425	-1.5567330	0.4620442

1 ion pair of cation **1**$^+$ and BF_4^-

51

Energy = -1423.874067963

C	3.2388542	-4.6181446	0.1329345
C	3.3303299	-3.5050266	0.9760794
C	2.1132146	-4.8276320	-0.6680804
H	4.0566728	-5.3317920	0.1020577
C	2.2904815	-2.5701750	1.0347592
H	4.2133428	-3.3649163	1.5915007
C	1.0718647	-3.9001966	-0.6156985
H	2.0544891	-5.6953898	-1.3182729
C	1.1755708	-2.7875759	0.2402003
H	2.3605870	-1.7080265	1.6917788
C	-0.2152160	-3.8478092	-1.3146133
C	-0.0860676	-1.9254673	0.1292055
C	-0.9187218	-2.7027245	-0.8966322
C	-0.7945499	-4.7083759	-2.2479336
C	-0.7649038	-1.5360561	1.4051723
N	0.2920812	-0.6319127	-0.4840182
C	-2.1748252	-2.3875040	-1.3908065
C	-2.0624974	-4.4003724	-2.7478630
H	-0.2728504	-5.5998542	-2.5829842
C	-0.7558103	-0.1413530	1.4689443
C	-1.3405438	-2.2825963	2.4215004
N	-0.0928730	0.3042787	0.2749315
C	-2.7482938	-3.2556439	-2.3266714
H	-2.7087322	-1.5004737	-1.0621032
H	-2.5247097	-5.0608538	-3.4753190
C	-1.3105640	0.5713106	2.5310229
C	-1.9029452	-1.5885167	3.4967005
H	-1.3547240	-3.3666363	2.3850966
C	0.1595979	1.7553495	-0.1228397
H	-3.7334854	-3.0383985	-2.7269990
C	-1.8870386	-0.1869320	3.5482848
H	-1.3056022	1.6509041	2.5842574
H	-2.3615770	-2.1433273	4.3088283
C	0.8848750	1.7691617	-1.4642107
C	-1.2164684	2.4281788	-0.2472665
C	1.0412837	2.3743608	0.9729288
H	-2.3318597	0.3227965	4.3961069

H 1.0545173 2.8171119 -1.7212129
H 1.8479707 1.2559238 -1.4036345
H 0.2848154 1.3004479 -2.2482914
H -1.7559768 2.4659072 0.7001771
H -1.0475704 3.4515644 -0.5887519
H -1.8292276 1.9059809 -0.9884901
H 1.2596148 3.4007269 0.6709415
H 0.5503984 2.3994803 1.9468394
H 1.9825698 1.8232745 1.0599944
B 0.6444712 5.7441795 -0.9333857
F 1.8995100 5.0825346 -0.9138263
F -0.1666563 5.1622712 -1.9416169
F 0.0124602 5.5696976 0.3242996
F 0.8276595 7.1135100 -1.1943454

1$^+$
46
Energy = -999.0255927172
C 3.3186116 3.0182236 -0.4561981
C 2.0185876 3.4614032 -0.1872285
C 3.6177972 1.6553942 -0.5294819
H 4.1089445 3.7471318 -0.6074838
C 0.9815241 2.5432641 0.0116260
H 1.8124405 4.5253423 -0.1284545
C 2.5900941 0.7324503 -0.3313886
H 4.6311299 1.3250745 -0.7366602
C 1.2881331 1.1930966 -0.0582339
H -0.0262154 2.8876439 0.2257070
C 2.5899900 -0.7325385 -0.3314542
C 0.3422752 0.0001117 0.1243638
C 1.2879120 -1.1930320 -0.0585926
C 3.6175416 -1.6556356 -0.5296251
C -0.5487459 -0.0000775 1.3260180
N -0.5962321 0.0003419 -1.0181775
C 0.9810279 -2.5431508 0.0108740
C 3.3181376 -3.0184285 -0.4565272
H 4.6309193 -1.3255021 -0.7369048
C -1.8674340 -0.0000797 0.8644392
C -0.2811175 -0.0003733 2.6859603
N -1.7810095 0.0002020 -0.5683528
C 2.0179587 -3.4614297 -0.1880241
H -0.0267853 -2.8874065 0.2247810
H 4.1084127 -3.7474505 -0.6075650
C -2.9694157 -0.0003165 1.7189360
C -1.3711900 -0.0007431 3.5613086
H 0.7372542 -0.0002761 3.0594376
C -2.9498381 0.0005985 -1.5433910
H 1.8115863 -4.5253523 -0.1297282
C -2.6904439 -0.0006747 3.0842262
H -3.9920459 -0.0002241 1.3676566
H -1.1955496 -0.0010471 4.6320953
C -2.3891865 0.0011173 -2.9635085
C -3.7590778 -1.2801081 -1.2842243
C -3.7589517 1.2811833 -1.2832017
H -3.5142402 -0.0008339 3.7897238
H -1.7828176 0.8890966 -3.1558935
H -1.7829247 -0.8867916 -3.1565597
H -3.2407679 0.0014633 -3.6486913
H -4.1936152 -1.3113002 -0.2841866
H -4.5749276 -1.3096444 -2.0107888
H -3.1348056 -2.1659997 -1.4307754
H -4.5748219 1.3113770 -2.0097176
H -4.1934565 1.3116236 -0.2831252
H -3.1345758 2.1671125 -1.4290754

1r radical from SET reduction of **1**$^+$
46
Energy = -999.1848840171
C 3.3852516 3.0191308 -0.3002655
C 2.0591332 3.4594251 -0.2142565
C 3.6879712 1.6546086 -0.3078530
H 4.1886793 3.7478896 -0.3625428
C 1.0081829 2.5382238 -0.1295371
H 1.8450697 4.5244843 -0.2127469
C 2.6398328 0.7346019 -0.2310896
H 4.7206001 1.3222065 -0.3727507
C 1.3096890 1.1844383 -0.1391032
H -0.0212234 2.8789084 -0.0566816
C 2.6397010 -0.7348351 -0.2328917
C 0.3428781 -0.0001431 -0.0526346
C 1.3095536 -1.1846675 -0.1415592
C 3.6879542 -1.6547911 -0.3084701
C -0.5217178 -0.0014649 1.1906278
N -0.6197811 0.0010911 -1.1793540
C 1.0080658 -2.5384181 -0.1318704
C 3.3854653 -3.0193350 -0.2978298

H	4.7204303	-1.3223555	-0.3756075
C	-1.8599876	-0.0016314	0.7669840
C	-0.1933752	-0.0007383	2.5345849
N	-1.8612291	-0.0001831	-0.6285386
C	2.0592719	-3.4596129	-0.2128889
H	-0.0215884	-2.8790881	-0.0625065
H	4.1891569	-3.7481350	-0.3561346
C	-2.9049088	-0.0011332	1.7023209
C	-1.2288722	0.0004363	3.4805322
H	0.8466303	-0.0009569	2.8495479
C	-3.0518722	0.0004741	-1.5239224
H	1.8454249	-4.5247145	-0.2089434
C	-2.5627110	0.0006604	3.0573681
H	-3.9471417	-0.0024639	1.4090090
H	-0.9969083	0.0010200	4.5410973
C	-2.5813045	0.0027304	-2.9817068
C	-3.8736327	-1.2738556	-1.2596167
C	-3.8748627	1.2732652	-1.2560458
H	-3.3589550	0.0027122	3.7965969
H	-1.9817492	0.8886382	-3.2012340
H	-1.9809918	-0.8820424	-3.2037649
H	-3.4694480	0.0032849	-3.6218179
H	-4.2334667	-1.3279062	-0.2296661
H	-4.7422430	-1.2879403	-1.9254251
H	-3.2652881	-2.1607343	-1.4619024
H	-4.7435244	1.2883579	-1.9217615
H	-4.2346835	1.3241019	-0.2259209
H	-3.2673892	2.1612925	-1.4558952

2r radical from SET reduction of $\mathbf{2}^+$

46

Energy = -999.1947617671

C	-2.5615070	-2.6661238	-1.9797613
C	-1.3475609	-2.4288938	-2.6360045
C	-2.8396535	-2.0677592	-0.7478732
H	-3.2963943	-3.3240577	-2.4350152
C	-0.3844997	-1.5873257	-2.0677511
H	-1.1519390	-2.9036960	-3.5931612
C	-1.8830395	-1.2220340	-0.1825415
H	-3.7849683	-2.2575763	-0.2467426
C	-0.6665290	-0.9893389	-0.8489007
H	0.5585019	-1.4003414	-2.5741261
C	-1.8970742	-0.4396366	1.0604826
C	0.2233230	-0.0606591	-0.0253134
C	-0.6897234	0.2786361	1.1582307
C	-2.8658965	-0.3191127	2.0588020
C	1.5181254	-0.7041786	0.4214486
N	0.7815645	1.0993363	-0.7903343
C	-0.4257038	1.0971398	2.2464664
C	-2.6104605	0.5228722	3.1447098
H	-3.8019360	-0.8676904	1.9983282
C	2.5736275	0.1244182	-0.0164751
C	1.7564886	-1.8621627	1.1389120
N	2.1293491	1.2190409	-0.7114920
C	-1.4028171	1.2238626	3.2407795
H	0.5177355	1.6294647	2.3280708
H	-3.3560420	0.6307397	3.9274174
C	3.9073119	-0.2152024	0.2722678
C	3.0864750	-2.2030020	1.4276086
H	0.9380600	-2.4941699	1.4739084
C	0.0399209	2.2635483	-1.3457167
H	-1.2206579	1.8677408	4.0963049
C	4.1419040	-1.3819849	0.9960470
H	4.7240346	0.4167885	-0.0634453
H	3.3017575	-3.1072028	1.9888531
C	-1.4376886	1.9180845	-1.5516431
C	0.1734408	3.4571536	-0.3809639
C	0.6742066	2.6119075	-2.7027050
H	5.1644026	-1.6648135	1.2313241
H	-1.5642222	1.0970286	-2.2613126
H	-1.9289466	1.6557873	-0.6114203
H	-1.9372763	2.8034991	-1.9561932
H	1.2297192	3.6582574	-0.1821452
H	-0.2791925	4.3475783	-0.8295942
H	-0.3344711	3.2491821	0.5649916
H	0.1453099	3.4650407	-3.1394863
H	1.7279280	2.8692445	-2.5793774
H	0.5968581	1.7615844	-3.3878924

2p⁺ P..C adduct of $\mathbf{2}^+$ and PMe_3

59

Energy = -1460.263142509

C	1.0885290	3.4246226	-2.1548549
C	0.4168677	2.3695228	-2.7837978
C	1.6710363	3.2573956	-0.8954803
H	1.1663833	4.3839316	-2.6580139

C 0.3059835 1.1236708 -2.1550592
H -0.0139563 2.5173659 -3.7696138
C 1.5674469 2.0141990 -0.2677656
H 2.2000582 4.0801094 -0.4228499
C 0.8794932 0.9642145 -0.9021544
H -0.1940652 0.2952599 -2.6503727
C 2.0951760 1.5255845 1.0129031
C 0.8582075 -0.2662456 -0.0025455
C 1.7319649 0.1730769 1.1693113
C 2.8512776 2.1637212 1.9977768
C -0.5563817 -0.6141421 0.4388440
N 1.2284907 -1.5418742 -0.6974936
C 2.0917830 -0.5452307 2.3001472
C 3.2338929 1.4352643 3.1272500
H 3.1409799 3.2053416 1.8923914
C -0.7822093 -1.9720962 0.0044642
C -1.4670887 0.1023554 1.1194830
N 0.2718753 -2.4739851 -0.6123915
C 2.8586621 0.0956463 3.2800518
H 1.7918370 -1.5814942 2.4259109
H 3.8279109 1.9155856 3.8991999
C -1.9952999 -2.6410193 0.3656737
C -2.8469855 -0.4768292 1.3216830
H -1.2584163 1.0974281 1.5013658
C 2.5646778 -1.9584282 -1.2137961
H 3.1631712 -0.4507908 4.1675863
C -2.9589830 -1.9560619 1.0251676
H -2.1088679 -3.7012359 0.1587458
H -3.2362210 -0.2465023 2.3237838
C 3.4471681 -0.7271783 -1.4376920
C 3.2264449 -2.9099271 -0.2010831
C 2.3418719 -2.6792679 -2.5524063
H -3.8592320 -2.4597182 1.3624616
H 3.0156661 -0.0526031 -2.1810387
H 3.6187480 -0.1753438 -0.5099880
H 4.4175291 -1.0705064 -1.8074143
H 2.5660959 -3.7568197 0.0069321
H 4.1667070 -3.2912946 -0.6117266
H 3.4450679 -2.3892924 0.7355248
H 3.3083811 -2.9948162 -2.9569877
H 1.7108665 -3.5603382 -2.4174704
H 1.8628265 -2.0076015 -3.2719573
P -4.0038304 0.4340734 0.1764100
C -5.6927408 -0.1629259 0.3824314
H -5.7361255 -1.2266589 0.1359296
H -6.0086633 -0.0124638 1.4183264
H -6.3581803 0.3910037 -0.2853782
C -3.4611985 0.1527216 -1.5159066
H -2.4613263 0.5758630 -1.6421618
H -3.4335421 -0.9220539 -1.7116129
H -4.1567739 0.6361034 -2.2064759
C -3.9491117 2.2029893 0.5264091
H -4.2408691 2.3819033 1.5646774
H -2.9346938 2.5755143 0.3615374
H -4.6388646 2.7268279 -0.1407169

2^{-} two-electron reduction of cation **2**$^{+}$
46
Energy = -999.2705453770
C -2.7023937 -2.6750815 -1.9142232
C -1.5043622 -2.4881804 -2.6170975
C -2.9178729 -2.0327381 -0.6911315
H -3.4716043 -3.3242759 -2.3253466
C -0.5016754 -1.6562775 -2.1065214
H -1.3562724 -2.9950680 -3.5676180
C -1.9142426 -1.2043324 -0.1818103
H -3.8534658 -2.1745928 -0.1549037
C -0.7122954 -1.0207463 -0.8909452
H 0.4262505 -1.4898158 -2.6463081
C -1.8584092 -0.3932715 1.0420463
C 0.2331181 -0.0874461 -0.1517835
C -0.6228089 0.2834637 1.0753889
C -2.7764470 -0.2506580 2.0868982
C 1.5368230 -0.7187761 0.3145621
N 0.7969620 0.9738637 -1.0582368
C -0.2781879 1.0632537 2.1733891
C -2.4442101 0.5731894 3.1657371
H -3.7272964 -0.7788027 2.0730209
C 2.5743607 0.2319607 0.0252702
C 1.7652235 -1.8864682 1.0088893
N 2.1669967 1.2984999 -0.6566616
C -1.2024975 1.2209802 3.2116144
H 0.6955929 1.5421174 2.2184007
H -3.1475675 0.6996844 3.9850651
C 3.8879625 -0.0824430 0.4926312
C 3.0700284 -2.1904081 1.4530521

H 0.9415641 -2.5686094 1.2204887
C 0.0355556 2.1945379 -1.4081235
H -0.9536339 1.8439616 4.0673040
C 4.1026408 -1.2691284 1.1874017
H 4.7128773 0.5996471 0.2945375
H 3.2730497 -3.1112767 1.9928358
C -1.4046053 1.8303781 -1.7853220
C 0.0264998 3.2661487 -0.2962302
C 0.7333355 2.7803178 -2.6503448
H 5.1122377 -1.4958348 1.5304431
H -1.4228045 1.0700783 -2.5720990
H -1.9633536 1.4534725 -0.9237328
H -1.9143393 2.7288796 -2.1526997
H 1.0458644 3.3899116 0.0810261
H -0.3309455 4.2236702 -0.6952202
H -0.6247870 2.9755523 0.5317812
H 0.2128046 3.6853609 -2.9872925
H 1.7707781 3.0244820 -2.4110657
H 0.7273973 2.0417938 -3.4595000

2 ion pair of cation $\mathbf{2}^+$ and BF_4^-
51
Energy = -1423.883648735
C 3.6046810 -4.6700731 0.9382483
C 3.4253761 -3.4472189 1.5939601
C 2.7329729 -5.0802466 -0.0745418
H 4.4319416 -5.3127242 1.2242994
C 2.3642491 -2.6032259 1.2459625
H 4.1112676 -3.1505149 2.3811942
C 1.6770467 -4.2409879 -0.4316281
H 2.8782977 -6.0347309 -0.5718473
C 1.5139252 -3.0118658 0.2315281
H 2.2221567 -1.6555512 1.7579329
C 0.6181116 -4.3853923 -1.4363342
C 0.2720813 -2.2964411 -0.3151973
C -0.2115968 -3.2474783 -1.4010885
C 0.3361793 -5.4214535 -2.3274330
C -0.7107391 -1.9634211 0.7663565
N 0.6176254 -0.8728270 -0.7725870
C -1.3347519 -3.1326659 -2.2086036
C -0.7783659 -5.3018925 -3.1613581
H 0.9638256 -6.3066909 -2.3696082
C -0.7533101 -0.5659515 0.8781247
C -1.4725691 -2.7554000 1.6039766
N 0.0688179 0.0263157 -0.0692631
C -1.6068282 -4.1757485 -3.1007220
H -1.9710732 -2.2544637 -2.1602145
H -1.0102886 -6.0989911 -3.8615445
C -1.5498396 0.1011925 1.8100517
C -2.2790996 -2.0998240 2.5460862
H -1.4599379 -3.8382715 1.5378604
C 1.4820931 -0.4636913 -1.9515060
H -2.4726087 -4.1088332 -3.7522612
C -2.3168564 -0.6995262 2.6503516
H -1.5809415 1.1829486 1.8450446
H -2.8981467 -2.6936823 3.2114818
C 2.7038800 -1.3809419 -2.0475209
C 0.6168409 -0.5797952 -3.2226515
C 1.8995552 0.9928197 -1.7343828
H -2.9637887 -0.2394001 3.3895206
H 3.2910191 -1.0426213 -2.9056424
H 3.3292331 -1.3243862 -1.1536402
H 2.4195843 -2.4198968 -2.2249254
H -0.3213847 -0.0363122 -3.0985603
H 1.1856573 -0.1286180 -4.0411108
H 0.4152021 -1.6220998 -3.4737840
H 1.0339127 1.6563439 -1.7475610
H 2.4338921 1.1145820 -0.7876226
H 2.5708681 1.2642587 -2.5536309
B -2.7258249 1.2176425 -1.7243419
F -2.5042542 -0.1864064 -1.6150425
F -1.5924701 1.8148674 -2.3281985
F -3.8672417 1.4473893 -2.5153184
F -2.9149401 1.7587497 -0.4337595

$\mathbf{2}^+$
46
Energy = -999.0312778363
C -2.5235776 -2.5679501 -2.0708447
C -1.2776911 -2.3560682 -2.6709883
C -2.8391912 -1.9917033 -0.8371479
H -3.2568845 -3.1925310 -2.5715605
C -0.3131373 -1.5578646 -2.0450255
H -1.0537650 -2.8173435 -3.6274447
C -1.8875949 -1.1871958 -0.2105994
H -3.8089781 -2.1660473 -0.3811400

C	-0.6394666	-0.9817707	-0.8275021
H	0.6564941	-1.3992125	-2.5080837
C	-1.9303626	-0.4362997	1.0481993
C	0.2323552	-0.1029371	0.0683673
C	-0.7099556	0.2448666	1.2203184
C	-2.9341078	-0.3006694	2.0070888
C	1.5275029	-0.7310055	0.4869106
N	0.7780558	1.1224477	-0.6545753
C	-0.4616905	1.0461445	2.3235887
C	-2.6976398	0.5171796	3.1157805
H	-3.8832970	-0.8161479	1.8968751
C	2.5638015	0.0943731	0.0201892
C	1.8166825	-1.8854792	1.1890639
N	2.0461308	1.1897707	-0.6548526
C	-1.4783196	1.1836867	3.2761638
H	0.4889936	1.5544649	2.4553749
H	-3.4721330	0.6346052	3.8674084
C	3.9171022	-0.1876350	0.2285174
C	3.1707747	-2.1812832	1.4064584
H	1.0370932	-2.5416159	1.5611374
C	0.0082411	2.2575440	-1.3209467
H	-1.3150534	1.8085960	4.1482206
C	4.2026388	-1.3504582	0.9364635
H	4.6937969	0.4703089	-0.1450769
H	3.4299383	-3.0809395	1.9555108
C	-1.4873520	1.9563124	-1.3781842
C	0.2794807	3.5112116	-0.4720831
C	0.5827034	2.4066848	-2.7371800
H	5.2341445	-1.6234814	1.1304325
H	-1.7071348	1.0813895	-1.9919270
H	-1.9207808	1.8282907	-0.3848802
H	-1.9652745	2.8233564	-1.8416849
H	1.3519221	3.7115755	-0.4126796
H	-0.2143092	4.3610505	-0.9502453
H	-0.1277978	3.3958754	0.5361589
H	0.0574368	3.2279978	-3.2311974
H	1.6495801	2.6354748	-2.7070394
H	0.4224702	1.4934057	-3.3177732

3a$^+$ P..C adduct of **1**$^+$ and PMe_3

59

Energy = -1460.243317198

C	2.2394356	-2.7465111	2.7885312
C	1.1765298	-1.9167167	3.1634866
C	2.9136601	-2.5490354	1.5801883
H	2.5516390	-3.5479987	3.4517076
C	0.7574408	-0.8774283	2.3237391
H	0.6790478	-2.0759082	4.1156628
C	2.5012966	-1.5103450	0.7424631
H	3.7476505	-3.1893265	1.3070262
C	1.4174143	-0.6945349	1.1172288
H	-0.0531227	-0.2187833	2.6247152
C	3.0187575	-1.0401329	-0.5493069
C	1.1252466	0.3313026	0.0225850
C	2.2485655	0.0608083	-0.9705012
C	4.0818978	-1.4907501	-1.3341985
C	-0.2738438	0.1704054	-0.5538179
N	1.1513721	1.7130965	0.5656757
C	2.5249162	0.7246265	-2.1564080
C	4.3632634	-0.8222237	-2.5286978
H	4.6847128	-2.3400521	-1.0253531
C	-0.9491019	1.3983739	-0.2703753
C	-0.8435457	-0.8493178	-1.2289470
N	-0.0462626	2.2090966	0.3559523
C	3.5969802	0.2757037	-2.9366532
H	1.9284105	1.5748020	-2.4754545
H	5.1897155	-1.1573115	-3.1487060
C	-2.2471435	1.6319930	-0.8123078
C	-2.3109655	-0.7695080	-1.5707120
H	-0.2859638	-1.7389148	-1.5037639
C	-0.3054075	3.6291890	0.8175002
H	3.8364816	0.7830940	-3.8664547
C	-2.8815812	0.6247095	-1.4679107
H	-2.7173588	2.6068863	-0.7691930
H	-2.5262141	-1.2122249	-2.5535333
C	0.9143162	4.1313450	1.5924080
C	-0.5175954	4.4981132	-0.4323228
C	-1.5335964	3.6141985	1.7392273
H	-3.8392615	0.8098540	-1.9431511
H	1.1160219	3.5071549	2.4654413
H	1.8062737	4.1478437	0.9639612
H	0.6941711	5.1498994	1.9260363
H	-1.3981607	4.2014560	-1.0051757
H	-0.6503772	5.5362965	-0.1155445
H	0.3603015	4.4445480	-1.0826989
H	-1.6984371	4.6281153	2.1140093

H	-2.4407248	3.2909240	1.2252525
H	-1.3584093	2.9547333	2.5945265
P	-3.2357591	-1.8803179	-0.3800877
C	-5.0037133	-1.8705890	-0.7401422
H	-5.3857845	-0.8505776	-0.6490866
H	-5.1699636	-2.2346669	-1.7576409
H	-5.5272951	-2.5177736	-0.0313196
C	-2.9576014	-1.2685130	1.2895201
H	-1.8887337	-1.3264771	1.5110137
H	-3.2916221	-0.2297537	1.3512975
H	-3.5166184	-1.8794725	2.0026405
C	-2.6056725	-3.5666726	-0.4991379
H	-2.7551836	-3.9432884	-1.5145834
H	-1.5385103	-3.5704817	-0.2615967
H	-3.1367508	-4.2083013	0.2089605

$3b^+$ PMe_3 aided H^+-shift from *para*-C to N
59
Energy = -1460.282017725

C	2.4351286	-2.8501255	3.0201832
C	1.9237657	-1.6289777	3.4750163
C	2.5621143	-3.1115645	1.6529810
H	2.7419168	-3.6030689	3.7405685
C	1.5215567	-0.6438680	2.5650462
H	1.8412462	-1.4447948	4.5421997
C	2.1671978	-2.1274886	0.7443196
H	2.9651860	-4.0608021	1.3111744
C	1.6470405	-0.9053202	1.2088345
H	1.1280823	0.3071298	2.9131580
C	2.1969809	-2.0983493	-0.7260337
C	1.2548651	-0.0145595	0.0384702
C	1.6834803	-0.8622838	-1.1626606
C	2.6375320	-3.0415571	-1.6560772
C	-0.2121582	0.3477496	-0.0219150
N	1.8818797	1.3420834	0.1128094
C	1.6045380	-0.5514208	-2.5135606
C	2.5637589	-2.7277561	-3.0164949
H	3.0354278	-4.0004484	-1.3356236
C	-0.3284751	1.7214223	-0.3330425
C	-1.3082867	-0.4613061	0.1442503
N	0.9141513	2.2870392	-0.4697453
C	2.0568124	-1.4955205	-3.4438808
H	1.2032737	0.4024224	-2.8464525
H	2.9066960	-3.4493138	-3.7524215
C	-1.6132596	2.2862630	-0.4587014
C	-2.6006812	0.0991225	0.0063372
H	-1.1682207	-1.5114575	0.3906826
C	1.2373930	3.7229018	-0.1547365
H	2.0113713	-1.2712418	-4.5055348
C	-2.7244468	1.4686756	-0.2934716
H	-1.7625010	3.3337111	-0.6777718
H	2.6883987	1.3533456	-0.5129090
C	2.7564300	3.9039745	-0.2923591
C	0.5599821	4.6294328	-1.1940225
C	0.7927195	4.0536883	1.2769832
H	-3.7071662	1.9183597	-0.3926471
H	3.2987220	3.3324386	0.4640607
H	3.0983729	3.6047189	-1.2894190
H	2.9931181	4.9627266	-0.1569209
H	-0.5216887	4.6929754	-1.0677715
H	0.9590407	5.6420332	-1.0831904
H	0.7781885	4.2795540	-2.2073887
H	1.0723626	5.0814126	1.5281888
H	-0.2922874	3.9574321	1.3859309
H	1.2808688	3.3750194	1.9831586
P	-4.0182860	-0.9402316	0.2238600
C	-5.5385877	0.0112766	0.0595590
H	-5.5716814	0.7969506	0.8181788
H	-5.5972110	0.4542195	-0.9375844
H	-6.3863680	-0.6642110	0.2028826
C	-3.9889195	-1.7117211	1.8576145
H	-3.0588689	-2.2739612	1.9772682
H	-4.0411259	-0.9338828	2.6233619
H	-4.8374710	-2.3935264	1.9619323
C	-4.0280216	-2.2637867	-1.0064511
H	-4.1016678	-1.8253206	-2.0047093
H	-3.0979573	-2.8337610	-0.9312301
H	-4.8760363	-2.9314608	-0.8302666

3p deprotonation of **$3a^+$**
58
Energy = -1459.772474700

C	-1.9357747	-2.9340853	-2.6057220
C	-1.5801004	-1.7203535	-3.2080612
C	-1.9822898	-3.0532065	-1.2134927
H	-2.1857186	-3.7895324	-3.2280076

C	-1.2561264	-0.6061976	-2.4246042
H	-1.5593158	-1.6455677	-4.2924177
C	-1.6639794	-1.9381829	-0.4333204
H	-2.2675233	-3.9966777	-0.7540098
C	-1.2948318	-0.7240354	-1.0428086
H	-0.9866906	0.3408114	-2.8853098
C	-1.6555427	-1.7475548	1.0255482
C	-0.9591808	0.3453104	0.0051727
C	-1.2803953	-0.4183719	1.2980549
C	-1.9643589	-2.6237015	2.0697507
C	0.5100884	0.7606907	-0.0548303
N	-1.7046206	1.6023459	-0.1541670
C	-1.2263783	0.0510132	2.6025504
C	-1.9017900	-2.1506763	3.3838929
H	-2.2545470	-3.6528908	1.8716449
C	0.5390098	2.1899099	-0.2148396
C	1.6383779	0.0051819	0.0219621
N	-0.7399613	2.6048851	-0.2620842
C	-1.5404036	-0.8235572	3.6497412
H	-0.9525858	1.0840032	2.8016868
H	-2.1433402	-2.8168826	4.2080783
C	1.8204576	2.8276594	-0.2888677
C	2.9301797	0.6382518	-0.0542080
H	1.5548195	-1.0742159	0.1412353
C	-1.3296215	3.9618244	-0.4156156
H	-1.5075040	-0.4723959	4.6780765
C	2.9614579	2.0599131	-0.2085753
H	1.9279016	3.8961518	-0.4070804
C	-2.1972603	3.9507262	-1.6869503
C	-2.2005708	4.2331369	0.8242085
C	-0.2706200	5.0589131	-0.5376183
H	3.9195381	2.5697183	-0.2679429
H	-1.5721104	3.7784658	-2.5700990
H	-2.9353694	3.1503600	-1.6202008
H	-2.7013026	4.9169463	-1.7970892
H	-1.5775095	4.2639364	1.7249472
H	-2.7066324	5.1983162	0.7146365
H	-2.9371803	3.4362538	0.9360905
H	-0.7940952	6.0141871	-0.6447522
H	0.3585600	5.1195178	0.3548002
H	0.3597817	4.9210702	-1.4205144
P	4.3591278	-0.3257247	0.0544216
C	5.8494924	0.6865033	-0.0945471
H	5.8569662	1.2001947	-1.0588719
H	5.8832592	1.4222924	0.7125061
H	6.7263581	0.0369741	-0.0256241
C	4.4462759	-1.6175106	-1.2276997
H	3.5369421	-2.2237712	-1.1799753
H	4.5025689	-1.1425357	-2.2102942
H	5.3163953	-2.2631472	-1.0737424
C	4.4874894	-1.2512916	1.6197039
H	4.5657535	-0.5427608	2.4481678
H	3.5797493	-1.8476311	1.7502847
H	5.3566321	-1.9163637	1.6128760

3$^+$ direct N..P adduct of **1**$^+$ and PMe_3

59

Energy = -1460.275411407

C	-4.1534801	-1.8162683	0.5951827
C	-3.0542323	-2.4257923	1.2094466
C	-4.0389769	-0.5510836	0.0086877
H	-5.1172819	-2.3162480	0.6062699
C	-1.8023659	-1.7975248	1.2042894
H	-3.1759369	-3.3822890	1.7080106
C	-2.7919694	0.0728735	0.0020814
H	-4.9120711	-0.0624541	-0.4135581
C	-1.6722055	-0.5811791	0.5485127
H	-0.9599732	-2.2435850	1.7237224
C	-2.3820770	1.4158655	-0.4262532
C	-0.4207056	0.2730096	0.3759162
C	-1.0147483	1.5903909	-0.1264574
C	-3.1320252	2.4586390	-0.9682937
C	0.4646948	0.2385632	1.5978487
N	0.5649019	-0.2982876	-0.6773150
C	-0.4071276	2.8215206	-0.3258500
C	-2.5071312	3.6886894	-1.1920948
H	-4.1856183	2.3277509	-1.1977039
C	1.7413164	-0.2197240	1.2684903
C	0.1168328	0.4874069	2.9207524
N	1.9300512	-0.3298789	-0.1439783
C	-1.1620215	3.8728062	-0.8617421
H	0.6298315	2.9890660	-0.0606007
H	-3.0763447	4.5140377	-1.6090637
C	2.6885911	-0.4791260	2.2568604
C	1.0699078	0.2617630	3.9173701
H	-0.8796604	0.8364526	3.1750734

C 2.9201587 0.7154316 -0.7363288
H -0.6969898 4.8418820 -1.0147951
C 2.3371282 -0.2289954 3.5856109
H 3.6718534 -0.8614748 2.0056160
H 0.8177796 0.4479686 4.9564711
C 2.3764520 1.2942903 -2.0429381
C 3.2071001 1.8672506 0.2434730
C 4.2248477 -0.0491665 -0.9914720
H 3.0604474 -0.4255713 4.3710756
H 2.2418505 0.5310875 -2.8086628
H 1.4263262 1.8110351 -1.8976733
H 3.1096387 2.0133052 -2.4196670
H 3.7894342 1.5340769 1.1051746
H 3.7882805 2.6298149 -0.2827011
H 2.2932711 2.3310709 0.6205987
H 4.9881469 0.6417419 -1.3638518
H 4.5993390 -0.5030845 -0.0696996
H 4.0888508 -0.8406943 -1.7334924
P 0.3363136 -1.7726708 -1.4590809
C -1.3126002 -1.8658891 -2.1821211
H -2.0622873 -2.1687266 -1.4518275
H -1.5742691 -0.8939107 -2.6073948
H -1.2642375 -2.6062580 -2.9867317
C 0.6919851 -3.2382456 -0.4636616
H 1.5958371 -3.0477075 0.1207100
H -0.1402967 -3.4780112 0.1961243
H 0.8718572 -4.0776176 -1.1415396
C 1.4590423 -1.8692415 -2.8609362
H 1.2220205 -1.0945998 -3.5919809
H 2.4958637 -1.7861348 -2.5354378
H 1.3054755 -2.8525399 -3.3171054

4a⁺ direct P..N adduct of **2⁺** and PMe_3
59
Energy = -1460.264835473
C 1.3732113 -2.6592153 -3.1868033
C 0.2247283 -1.8732096 -3.3045947
C 2.3005844 -2.4221253 -2.1649858
H 1.5685816 -3.4372032 -3.9188121
C -0.0429967 -0.8667173 -2.3653359
H -0.4539791 -2.0293730 -4.1378845
C 2.0366922 -1.4129159 -1.2404398
H 3.2207227 -2.9976436 -2.1178057
C 0.8397602 -0.6754774 -1.3172804
H -0.9017813 -0.2165539 -2.4917859
C 2.8771306 -0.8123136 -0.1964890
C 0.7345691 0.2687618 -0.1124054
C 2.1730106 0.2635861 0.3860250
C 4.2051270 -1.0576013 0.1469293
C 0.0983731 1.6074924 -0.3926741
N -0.3041835 -0.4210517 0.8187695
C 2.8364244 1.1775494 1.1937366
C 4.8431192 -0.1832912 1.0324382
H 4.7499503 -1.8897578 -0.2895126
C -1.2564990 1.5446592 -0.0695233
C 0.6386786 2.7798328 -0.9025344
N -1.5525218 0.2437227 0.4501714
C 4.1771592 0.9429412 1.5232666
H 2.3399833 2.0677172 1.5642760
H 5.8792120 -0.3573044 1.3072538
C -2.1076780 2.6312193 -0.2254889
C -0.2039808 3.8841717 -1.0839454
H 1.6928098 2.8385463 -1.1562056
C -0.1463871 -0.4111387 2.3304710
H 4.7034360 1.6468459 2.1605563
C -1.5577820 3.8083984 -0.7491457
H -3.1510409 2.5929556 0.0705680
H 0.2013462 4.8108233 -1.4775493
C 1.0530685 -1.3007448 2.6967626
C -0.0595807 0.9898756 2.9499010
C -1.3983926 -1.0963377 2.8990741
H -2.1946624 4.6782216 -0.8761594
H 1.0002659 -1.5218525 3.7672718
H 1.0015377 -2.2450281 2.1460144
H 2.0148235 -0.8321083 2.4994397
H -0.9310808 1.5886059 2.6671073
H -0.0525319 0.9014751 4.0413707
H 0.8424229 1.5221509 2.6530756
H -1.2368537 -1.3019800 3.9605374
H -2.2719174 -0.4468754 2.8212297
H -1.5919395 -2.0506956 2.3974534
P -2.8259113 -0.7047823 -0.1280690
C -2.2406705 -2.3862835 -0.4107079
H -1.1821395 -2.4167659 -0.1391185
H -2.8129315 -3.0839835 0.2045970
H -2.3626266 -2.6367708 -1.4663023

C	-3.5374751	0.0133293	-1.6245094
H	-4.0640329	0.9386951	-1.3851357
H	-2.7809544	0.2115837	-2.3827023
H	-4.2600776	-0.7128415	-2.0096377
C	-4.2066892	-0.7284557	1.0310081
H	-3.9777626	-1.3265549	1.9125501
H	-4.4518126	0.2947276	1.3288871
H	-5.0650732	-1.1664837	0.5110338

4b$^+$ C-to-N H-shift of adduct **2p$^+$**
59
Energy = -1460.278176286

C	1.7582739	-1.8855972	3.4900075
C	1.3798865	-0.5443909	3.6235224
C	1.8796632	-2.4711288	2.2278736
H	1.9478003	-2.4802363	4.3790635
C	1.1296905	0.2396687	2.4924777
H	1.2775041	-0.1099690	4.6137020
C	1.6368341	-1.6871936	1.0972769
H	2.1492277	-3.5194157	2.1327839
C	1.2905404	-0.3285997	1.2348174
H	0.8262282	1.2767254	2.6019083
C	1.6419767	-2.0371494	-0.3296433
C	0.9950303	0.2776397	-0.1516398
C	1.2910189	-0.8976323	-1.0736525
C	1.9332481	-3.2374513	-0.9816766
C	-0.4594783	0.7202709	-0.1791656
N	1.6648681	1.5349386	-0.6170366
C	1.2246419	-0.9310143	-2.4580696
C	1.8687517	-3.2736346	-2.3771647
H	2.2109229	-4.1264207	-0.4221260
C	-0.5035136	2.1244683	-0.2085651
C	-1.6126641	-0.0262338	-0.1199047
N	0.7718539	2.6310802	-0.2233740
C	1.5191238	-2.1333677	-3.1109435
H	0.9662025	-0.0371905	-3.0186185
H	2.0969422	-4.1976653	-2.9008040
C	-1.7269676	2.8082911	-0.1965545
C	-2.8566851	0.6467913	-0.0972957
H	-1.5552481	-1.1123402	-0.1013601
C	3.0914634	1.8495863	-0.2897028
H	1.4799725	-2.1834444	-4.1952830
C	-2.8942461	2.0558989	-0.1360082
H	-1.7701810	3.8918054	-0.2351429
H	0.9303432	3.4499127	-0.8053764
C	3.9402363	0.6217132	-0.6344554
C	3.4906972	3.0061382	-1.2259394
C	3.3419585	2.2727266	1.1692338
H	-3.8464102	2.5760840	-0.1267172
H	3.7090587	-0.2212081	0.0221908
H	3.7885813	0.3135840	-1.6723105
H	4.9947594	0.8782247	-0.4946294
H	2.9822971	3.9404201	-0.9610189
H	4.5659006	3.1902192	-1.1412254
H	3.2552846	2.7539536	-2.2643255
H	4.3506378	2.6908141	1.2513887
H	2.6257033	3.0402050	1.4776734
H	3.2691279	1.4251554	1.8524517
P	-4.3435067	-0.3188526	-0.0177669
C	-5.7979258	0.7427478	-0.0428064
H	-5.7871271	1.4128875	0.8201412
H	-5.8198756	1.3255433	-0.9668079
H	-6.6881758	0.1096820	0.0050427
C	-4.3704423	-1.3039963	1.4965512
H	-3.4793031	-1.9364028	1.5324244
H	-4.3719184	-0.6342930	2.3601215
H	-5.2628319	-1.9357340	1.5145792
C	-4.4289886	-1.4565340	-1.4183676
H	-4.4692916	-0.8811266	-2.3464729
H	-3.5393034	-2.0918976	-1.4257727
H	-5.3196740	-2.0849239	-1.3316765

4c$^+$ C-to-N-*t*Bu H-shift of adduct **2p$^+$**
59
Energy = -1460.266347096

C	1.8845526	-1.8656998	3.4338251
C	1.3295556	-0.5877204	3.5676079
C	2.0967781	-2.4284240	2.1724439
H	2.1373356	-2.4346646	4.3236548
C	0.9916899	0.1626997	2.4366170
H	1.1545064	-0.1777980	4.5575722
C	1.7672583	-1.6793672	1.0420523
H	2.4963025	-3.4341179	2.0796511
C	1.2483689	-0.3766106	1.1832181
H	0.5478091	1.1482847	2.5394354
C	1.8131900	-2.0188370	-0.3867849

C	0.8973623	0.1865410	-0.1841670
C	1.2943015	-0.9353850	-1.1210428
C	2.2559919	-3.1627266	-1.0497824
C	-0.5235182	0.6914996	-0.2435867
N	1.6202383	1.5323165	-0.6229250
C	1.1983929	-0.9796041	-2.5044683
C	2.1755366	-3.2028321	-2.4456391
H	2.6594676	-4.0077940	-0.4996417
C	-0.4924585	2.1191864	-0.2827257
C	-1.6904046	-0.0174321	-0.1386760
N	0.7211900	2.6730462	-0.3830101
C	1.6517668	-2.1262920	-3.1685782
H	0.7730767	-0.1530353	-3.0688632
H	2.5205115	-4.0851114	-2.9765246
C	-1.7277009	2.8190948	-0.1962337
C	-2.9232090	0.6847908	-0.0791537
H	-1.6613132	-1.1044374	-0.0911196
C	3.0794072	1.8758821	-0.2162780
H	1.5896089	-2.1819094	-4.2509140
C	-2.9042779	2.1024501	-0.1014497
H	-1.7413582	3.9038751	-0.2188243
H	1.6788898	1.4282186	-1.6476182
C	3.9604323	0.6532779	-0.4694527
C	3.4753384	3.0160143	-1.1634949
C	3.1404347	2.3404281	1.2352701
H	-3.8391847	2.6524840	-0.0501174
H	3.7284119	-0.1633418	0.2163363
H	3.8700531	0.2939232	-1.4996133
H	5.0005119	0.9491058	-0.3054450
H	2.8486503	3.8940317	-1.0010323
H	4.5202663	3.2768442	-0.9756249
H	3.3889226	2.7021221	-2.2108119
H	4.1117170	2.8177779	1.3940232
H	2.3541102	3.0726349	1.4321861
H	3.0515268	1.5085420	1.9342892
P	-4.4260073	-0.2214021	0.0374424
C	-5.8422158	0.8916862	0.1049824
H	-5.7687347	1.5380905	0.9827518
H	-5.8828699	1.5005840	-0.8013130
H	-6.7526490	0.2904481	0.1760584
C	-4.4528116	-1.2613042	1.5192469
H	-3.5905562	-1.9337012	1.5053806
H	-4.3959904	-0.6240412	2.4051723
H	-5.3707658	-1.8551624	1.5464765
C	-4.6407320	-1.3285524	-1.3784120
H	-4.6996625	-0.7339510	-2.2933718
H	-3.7817212	-2.0022366	-1.4436409
H	-5.5528291	-1.9202564	-1.2595256

4d$^+$ C…N ring-opening of **4c$^+$**

59

Energy = -1460.270462899

C	1.5882906	-3.8436731	2.6806316
C	0.4388194	-3.1136749	3.0095422
C	2.3364882	-3.5160900	1.5457700
H	1.9108064	-4.6574733	3.3233165
C	0.0034077	-2.0598650	2.2030579
H	-0.1139262	-3.3610806	3.9112181
C	1.9047386	-2.4721764	0.7307179
H	3.2452818	-4.0642127	1.3128673
C	0.7155333	-1.7489679	1.0377832
H	-0.8613339	-1.4774967	2.5075264
C	2.4954560	-1.8799934	-0.4662339
C	0.5460529	-0.6780221	0.0495057
C	1.6681428	-0.7869184	-0.8700939
C	3.6252156	-2.2186164	-1.2005242
C	-0.5820297	0.1705624	-0.0850711
N	1.6535121	2.0225537	-0.1940431
C	1.9810805	-0.0671209	-2.0344167
C	3.9421787	-1.4721686	-2.3446271
H	4.2540188	-3.0531898	-0.9029797
C	-0.5657439	1.5401013	-0.5883384
C	-1.8652744	-0.3599049	0.2346649
N	0.4704619	2.4066431	-0.5635862
C	3.1232599	-0.4183215	-2.7609613
H	1.3374834	0.7290565	-2.3925288
H	4.8268225	-1.7247105	-2.9216606
C	-1.7993426	2.1592631	-0.9695646
C	-3.0382745	0.2893986	-0.0959856
H	-1.8960365	-1.3618585	0.6458045
C	2.6936245	3.0337084	0.1302144
H	3.3743961	0.1344235	-3.6613256
C	-3.0078822	1.5654291	-0.7444935
H	-1.7369766	3.1514957	-1.4048805
H	1.7956767	1.0661383	0.1538022
C	3.9777408	2.2372611	0.3812736

C	2.8464254	3.9734291	-1.0712635
C	2.2608678	3.8098081	1.3851671
H	-3.9261315	2.0690614	-1.0303318
H	3.8499833	1.5402613	1.2178486
H	4.2625563	1.6690410	-0.5095460
H	4.7902012	2.9224919	0.6352831
H	1.9037483	4.4850969	-1.2800003
H	3.6127154	4.7212568	-0.8487614
H	3.1498950	3.4112853	-1.9598001
H	3.0172735	4.5597982	1.6347127
H	1.3081566	4.3156842	1.2036102
H	2.1465598	3.1342521	2.2394216
P	-4.6136257	-0.4876777	0.2154055
C	-5.6265844	0.5922914	1.2464930
H	-5.1273176	0.7468182	2.2062185
H	-5.7566423	1.5558895	0.7471677
H	-6.6052829	0.1317655	1.4069983
C	-4.3889069	-2.0625280	1.0601793
H	-3.8137002	-2.7481568	0.4333325
H	-3.8771212	-1.9062370	2.0130335
H	-5.3749333	-2.4952534	1.2501777
C	-5.4851599	-0.7719523	-1.3391204
H	-5.6093293	0.1784122	-1.8648271
H	-4.9001766	-1.4549249	-1.9603533
H	-6.4679555	-1.2057664	-1.1349246

4e^{+} N..H..C proton transfer of **4d**$^{+}$

59

Energy = -1460.276610510

C	-1.9759929	4.3073180	-1.6836479
C	-1.6811610	3.3051737	-2.6161832
C	-1.4408767	4.2655416	-0.3945009
H	-2.6276416	5.1284393	-1.9684282
C	-0.8403704	2.2400939	-2.2736063
H	-2.1044441	3.3585979	-3.6150074
C	-0.6041688	3.2006653	-0.0484034
H	-1.6741853	5.0484234	0.3217563
C	-0.3058401	2.1954472	-0.9910253
H	-0.6127993	1.4647677	-3.0009313
C	0.0834407	2.8832838	1.2079198
C	0.6099329	1.1518869	-0.3797595
C	0.7999964	1.6806321	1.0395881
C	0.1136508	3.5551177	2.4328440
C	0.0391949	-0.2526530	-0.3901805
N	3.0184413	-2.0104094	-0.1548408
C	1.5349074	1.1358397	2.0848592
C	0.8595578	3.0098348	3.4799105
H	-0.4311063	4.4846580	2.5731665
C	0.8797618	-1.3783928	-0.4685510
C	-1.3323638	-0.4636280	-0.2609186
N	2.2730727	-1.1368834	-0.6459200
C	1.5632185	1.8117314	3.3101808
H	2.0768149	0.2019325	1.9629557
H	0.8939424	3.5200304	4.4382022
C	0.3534867	-2.6766816	-0.4519165
C	-1.8637830	-1.7597830	-0.2350725
H	-1.9814752	0.4060320	-0.1928488
C	4.4705055	-1.7909390	-0.3941083
H	2.1364330	1.4033139	4.1374016
C	-1.0152900	-2.8745242	-0.3384158
H	1.0344040	-3.5164584	-0.5402846
H	1.5780450	1.1174182	-0.8960076
C	4.9422734	-3.0664634	-1.1181224
C	4.7912891	-0.5366599	-1.2048435
C	5.0935882	-1.7247829	1.0127514
H	-1.4133331	-3.8836906	-0.3360304
H	4.6706659	-3.9577508	-0.5450098
H	4.4911517	-3.1346225	-2.1131963
H	6.0304856	-3.0363201	-1.2291424
H	4.4407816	0.3640324	-0.6922527
H	5.8751558	-0.4634867	-1.3372010
H	4.3193394	-0.5719925	-2.1909629
H	6.1837012	-1.6903831	0.9256418
H	4.7587367	-0.8259689	1.5408838
H	4.8126487	-2.6040445	1.5993710
P	-3.6388567	-1.9381902	-0.0809444
C	-4.1775180	-1.2372200	1.4913257
H	-3.7111049	-1.7898399	2.3106343
H	-3.8760203	-0.1876081	1.5436484
H	-5.2659647	-1.3057828	1.5698736
C	-4.1088960	-3.6731418	-0.1507569
H	-3.8024131	-4.1046306	-1.1067961
H	-3.6420845	-4.2197453	0.6723028
H	-5.1961879	-3.7406276	-0.0562744
C	-4.4511152	-1.0444155	-1.4209469
H	-4.1555290	0.0077725	-1.3905782

H -4.1508750 -1.4789413 -2.3774807
H -5.5358050 -1.1179573 -1.3039886

4p.$HPMe_3^+$ loose complex of **4p** and PMe_3H^+
72
Energy = -1921.487607046

C	2.0292608	-4.3788759	1.3197247
C	1.5358977	-3.5297457	2.3181820
C	2.1711778	-3.9277816	0.0056922
H	2.2936163	-5.4027588	1.5686814
C	1.1880056	-2.2080483	2.0215695
H	1.4204019	-3.9029486	3.3318717
C	1.8287819	-2.6055944	-0.2892958
H	2.5331923	-4.5985222	-0.7689798
C	1.3628254	-1.7463109	0.7227761
H	0.7975360	-1.5537039	2.7951155
C	1.8297796	-1.8762781	-1.5635288
C	0.9965374	-0.3756667	0.1297204
C	1.3625056	-0.5672176	-1.3359471
C	2.2046393	-2.2824749	-2.8459316
C	-0.4720962	-0.0873279	0.3961564
N	1.5810014	0.8642632	0.7618944
C	1.2579401	0.3316337	-2.3876485
C	2.1076055	-1.3688357	-3.8982550
H	2.5692185	-3.2898915	-3.0272852
C	-0.5020113	0.8557886	1.4833826
C	-1.6022490	-0.6398511	-0.1325003
N	0.6859179	1.3535229	1.7912960
C	1.6367217	-0.0706347	-3.6729875
H	0.8963004	1.3408339	-2.2204490
H	2.3987372	-1.6682564	-4.9010353
C	-1.7697825	1.1567025	2.0771808
C	-2.8713176	-0.2904197	0.4221409
H	-1.5302283	-1.3479290	-0.9541901
C	3.0142362	0.9775230	1.1675096
H	1.5653693	0.6278744	-4.5019205
C	-2.9082185	0.5941147	1.5435591
H	-1.8327009	1.8216065	2.9338863
H	0.8965274	2.3304319	-0.1900785
C	3.3176503	0.2871918	2.5093287
C	3.9091899	0.4010444	0.0674279
C	3.2985140	2.4837905	1.3050707
H	-3.8669728	0.8425963	1.9949684
H	2.5913939	0.6041527	3.2625324
H	3.2816291	-0.7998514	2.4153818
H	4.3215972	0.5689711	2.8450384
H	3.7092831	0.8751950	-0.8978839
H	4.9545448	0.5847122	0.3354277
H	3.7732176	-0.6785779	-0.0367766
H	4.3360914	2.6397058	1.6166339
H	3.1532952	2.9886546	0.3426198
H	2.6349917	2.9298101	2.0499367
P	-4.3615942	-0.9335689	-0.2199381
C	-5.4545108	0.3595239	-0.8747867
H	-5.6599822	1.0867880	-0.0841914
H	-4.9537566	0.8652711	-1.7043818
H	-6.3979041	-0.0735619	-1.2201933
C	-5.3316001	-1.8056590	1.0390003
H	-4.7560432	-2.6578471	1.4088327
H	-5.5353470	-1.1260650	1.8710281
H	-6.2792563	-2.1512049	0.6158836
C	-4.0248617	-2.0930105	-1.5624783
H	-3.4985390	-1.5851715	-2.3742848
H	-3.4197693	-2.9252543	-1.1946924
H	-4.9751817	-2.4797992	-1.9397991
P	0.0450416	3.4690636	-0.4023838
C	0.8379340	4.5062569	-1.6552458
H	0.9789948	3.9296007	-2.5737461
H	0.2201552	5.3820898	-1.8699090
H	1.8145723	4.8331850	-1.2877072
C	-1.5640512	2.9425239	-1.0301689
H	-2.1652394	3.8210501	-1.2805486
H	-1.4180971	2.3306528	-1.9238587
H	-2.0669936	2.3457210	-0.2643455
C	-0.1683696	4.4477066	1.0969321
H	-0.7239623	5.3623008	0.8685695
H	-0.7064254	3.8481912	1.8331083
H	0.8158336	4.7035895	1.4960778

4p C…H deprotonation of **2p**$^+$
58
Energy = -1459.800921332

C	-2.0413809	-3.1816442	-2.4565346
C	-1.6577320	-2.0371860	-3.1678963
C	-2.0792023	-3.1749890	-1.0596305

H	-2.3166087	-4.0829133	-2.9980365
C	-1.3029111	-0.8648385	-2.4915413
H	-1.6397634	-2.0619777	-4.2543149
C	-1.7254366	-2.0036011	-0.3849756
H	-2.3851871	-4.0648496	-0.5151427
C	-1.3386118	-0.8588719	-1.1049137
H	-1.0163766	0.0325340	-3.0326719
C	-1.6889792	-1.6867718	1.0489639
C	-0.9833691	0.2849034	-0.1659955
C	-1.2881162	-0.3448768	1.2059767
C	-1.9534260	-2.4824532	2.1670078
C	0.4797632	0.7000254	-0.2075005
N	-1.6152205	1.5847427	-0.5693747
C	-1.1036590	0.1932117	2.4734583
C	-1.8047654	-1.9250742	3.4394342
H	-2.2614885	-3.5191246	2.0550473
C	0.4905011	2.1438209	-0.2520364
C	1.6142275	-0.0438047	-0.1244271
N	-0.7060540	2.6748588	-0.3713796
C	-1.3781275	-0.6000404	3.5927283
H	-0.7545670	1.2146101	2.5939165
H	-2.0103623	-2.5285405	4.3195635
C	1.7637545	2.8035136	-0.1885244
C	2.8892811	0.6195792	-0.0787319
H	1.5546753	-1.1311814	-0.0817702
C	-3.0188449	1.9423624	-0.2550129
H	-1.2550641	-0.1869797	4.5903714
C	2.9112779	2.0518285	-0.1063165
H	1.8169082	3.8885688	-0.2164104
C	-3.9245034	0.7273043	-0.4822364
C	-3.2057812	2.4738345	1.1813239
C	-3.4134970	3.0470580	-1.2519653
H	3.8675536	2.5675742	-0.0662772
H	-3.8188754	0.3429799	-1.5008557
H	-3.7011816	-0.0795861	0.2217398
H	-4.9658129	1.0301224	-0.3282859
H	-2.4595683	3.2480045	1.3831775
H	-4.2059719	2.9082910	1.2935439
H	-3.0962273	1.6742723	1.9179300
H	-4.4557952	3.3444154	-1.0890768
H	-2.7677709	3.9187637	-1.1234588
H	-3.3038782	2.6796329	-2.2777291
P	4.3315671	-0.3293586	0.0129895
C	5.8047264	0.7155702	0.0857258
H	5.8684118	1.3335709	-0.8129919
H	5.7639775	1.3571047	0.9691192
H	6.6888927	0.0751436	0.1456708
C	4.5395568	-1.4575352	-1.4010049
H	3.6477898	-2.0855674	-1.4845641
H	4.6421746	-0.8679825	-2.3155280
H	5.4180812	-2.0961210	-1.2667788
C	4.3718772	-1.4216944	1.4691129
H	4.3722218	-0.8089574	2.3740849
H	3.4744499	-2.0470076	1.4668966
H	5.2576511	-2.0639359	1.4519113

4$^+$ final product cation of **2**$^+$ and PMe_3

59

Energy = -1460.277726580

C	4.1450244	2.1588755	1.5795867
C	3.1342621	1.5261854	2.3126995
C	3.9844335	2.4197256	0.2166712
H	5.0681219	2.4439561	2.0760626
C	1.9358961	1.1508635	1.6945144
H	3.2822721	1.3244819	3.3695474
C	2.7937063	2.0375342	-0.4056740
H	4.7780719	2.9035425	-0.3458807
C	1.7740819	1.4103025	0.3400245
H	1.1545731	0.6579179	2.2677077
C	2.3638156	2.1317309	-1.8030905
C	0.5704233	1.1346846	-0.5489325
C	1.0801631	1.5626431	-1.9200929
C	3.0155110	2.6327180	-2.9321110
C	0.0081043	-0.2676216	-0.4825053
N	2.9607373	-2.0601959	-0.5435676
C	0.4438292	1.4803663	-3.1506568
C	2.3753775	2.5484247	-4.1704780
H	4.0058561	3.0730429	-2.8557379
C	0.8177354	-1.4056790	-0.6737948
C	-1.3434998	-0.4655395	-0.2124298
N	2.1856254	-1.1992538	-1.0094097
C	1.1032081	1.9754532	-4.2820537
H	-0.5434125	1.0341155	-3.2426173
H	2.8712859	2.9284653	-5.0590743
C	0.2754577	-2.6929616	-0.6291149
C	-1.8952658	-1.7584506	-0.1617726

H	-1.9688679	0.4084720	-0.0440713
C	4.3814369	-1.9012584	-0.9528668
H	0.6241376	1.9148580	-5.2550524
C	-1.0792965	-2.8769389	-0.3773713
H	0.9295078	-3.5406678	-0.8037379
H	-0.2407873	1.8168104	-0.2556662
C	4.8066330	-3.2981795	-1.4393135
C	4.6059814	-0.8406144	-2.0285645
C	5.1231380	-1.5451562	0.3504630
H	-1.4864049	-3.8821308	-0.3505717
H	4.6034305	-4.0516895	-0.6727289
H	4.2676958	-3.5704942	-2.3527671
H	5.8791325	-3.2950759	-1.6566140
H	4.3115017	0.1480423	-1.6729866
H	5.6673594	-0.8199602	-2.2956982
H	4.0219176	-1.0631045	-2.9267560
H	6.2005739	-1.5290463	0.1586269
H	4.8112030	-0.5596489	0.7081718
H	4.9147772	-2.2869785	1.1273167
P	-3.6445372	-1.9370395	0.1701219
C	-4.0397989	-1.1811773	1.7593953
H	-3.4860662	-1.6939141	2.5497156
H	-3.7561086	-0.1257016	1.7421264
H	-5.1141552	-1.2640374	1.9456529
C	-4.1014628	-3.6769128	0.2138031
H	-3.8941338	-4.1435479	-0.7522501
H	-3.5460587	-4.1896167	1.0029548
H	-5.1723818	-3.7456930	0.4239010
C	-4.5987759	-1.1060230	-1.1154544
H	-4.3180036	-0.0502201	-1.1515545
H	-4.3859171	-1.5725353	-2.0804617
H	-5.6657410	-1.1895076	-0.8907718

BF_4^- counter anion in **1** and **2**

5

Energy = -424.8195909236

B	-0.4675061	-0.2862278	-0.0000240
F	-1.8832201	-0.2863414	0.0000007
F	0.0042110	-1.6210343	0.0000033
F	0.0043688	0.3811564	1.1558280
F	0.0043602	0.3811489	-1.1558080

KBF_4 : contact ion pair of K^+ and BF_4^-

6

Energy = -1024.726876638

B	-0.4958967	-0.2643462	0.0000486
F	-1.8770131	-0.3314507	0.0002907
F	0.0968145	-1.5624534	-0.0000213
F	0.0000686	0.4235241	1.1476965
F	-0.0003116	0.4235136	-1.1477087
K	2.4385521	-0.1200854	-0.0003058

$(KPPh_2)_2$: $KPPh_2$ dimer in solution

48

Energy = -2809.956058411

P	-2.2908930	1.1989926	1.6232406
C	-2.9368483	0.0362232	0.3655787
C	-2.6715937	-1.3449684	0.5215279
C	-3.1569043	-2.2937874	-0.3785838
C	-3.9170993	-1.8931699	-1.4800266
C	-4.2040029	-0.5347744	-1.6505194
C	-3.7361150	0.4105501	-0.7387186
H	-4.0065232	1.4545343	-0.8709222
K	0.0549235	-0.0000047	-0.3759127
P	2.2420661	-0.7565605	1.9977524
C	1.5084072	-2.2591053	1.2788099
C	1.5020041	-2.6650637	-0.0798458
C	0.7741933	-3.7758778	-0.5139888
C	0.0360986	-4.5466804	0.3838443
C	0.0517987	-4.1881925	1.7392568
C	0.7698206	-3.0815406	2.1726134
H	0.7638457	-2.8299726	3.2333708
H	-0.5041743	-4.7790685	2.4635711
H	-0.5289240	-5.4091177	0.0437040
H	0.7948932	-4.0404979	-1.5689752
H	2.0851624	-2.1106370	-0.8116556
C	2.9741997	0.1489725	0.5850978
C	3.8375628	-0.4225239	-0.3773970
C	4.3599281	0.3347256	-1.4251958
C	4.0660453	1.6976310	-1.5362099
C	3.2444762	2.2955062	-0.5773833
C	2.7053894	1.5324894	0.4582987
H	2.0425023	2.0109217	1.1784290
K	-0.1056465	0.4317974	3.8681638
C	-0.8388687	3.5383072	1.4368455
C	-0.0915378	4.5467387	0.8427982

C	0.0285874	4.6233116	-0.5519864
C	-0.6353140	3.6731305	-1.3275571
C	-1.3927736	2.6611583	-0.7328331
C	-1.5043686	2.5397024	0.6752803
H	-1.9155826	1.9591366	-1.3784053
H	-0.5729477	3.7163424	-2.4126446
H	0.6166572	5.4087857	-1.0167540
H	0.4049050	5.2824431	1.4714212
H	-0.9150719	3.5077958	2.5239343
H	2.9987911	3.3517676	-0.6457601
H	4.4736223	2.2830237	-2.3553734
H	5.0127010	-0.1398284	-2.1540007
H	4.1138824	-1.4695730	-0.2899909
H	-4.8091273	-0.2100088	-2.4936529
H	-4.2833438	-2.6264862	-2.1927312
H	-2.9183375	-3.3432042	-0.2284425
H	-2.0571320	-1.6744754	1.3585461

$(KPPh_2)_2^+$: radical cation
48
Energy = -2809.818192292

P	0.5270066	-0.7761222	1.7078998
K	2.7527795	3.2116698	-1.3866640
P	0.2744281	0.6817550	-0.2980909
K	-2.8944320	0.0592067	-0.6141895
C	-2.0754168	-1.9254801	2.1289266
C	-3.4287528	-1.8258888	2.4557151
C	-4.0004157	-0.5816310	2.7639901
C	-3.1938172	0.5613543	2.7518227
C	-1.8417975	0.4677315	2.4104437
C	-1.2509945	-0.7805538	2.0867969
H	-1.2256194	1.3638986	2.4088088
H	-3.6172530	1.5291305	3.0091311
H	-5.0515310	-0.5106131	3.0272500
H	-4.0395151	-2.7245368	2.4824075
H	-1.6486888	-2.8999683	1.9106302
C	1.9305390	-0.3161668	-2.2884327
C	3.1305254	-0.7066665	-2.8721818
C	4.3384571	-0.5661635	-2.1693667
C	4.3199230	-0.0614352	-0.8694801
C	3.1121129	0.3085638	-0.2684686
C	1.8917364	0.2128308	-0.9800844
H	3.1118041	0.6337701	0.7661973
H	5.2455692	0.0222877	-0.3051851
H	5.2765595	-0.8629112	-2.6290232
H	3.1310515	-1.1216147	-3.8764366
H	0.9989307	-0.4188906	-2.8402399
C	0.5076449	2.3086713	0.4835569
C	-0.2726194	3.3851428	-0.0027822
C	-0.1066281	4.6810223	0.4753334
C	0.8595247	4.9501349	1.4636586
C	1.6137032	3.8978323	1.9848616
C	1.4373219	2.5905452	1.5171454
H	1.9873734	1.7776095	1.9789790
H	2.3400835	4.0892058	2.7709678
H	1.0050976	5.9632616	1.8261505
H	-0.7182525	5.4860253	0.0770374
H	-0.9975552	3.1913374	-0.7922155
C	-0.0818552	-2.7473511	-0.2825187
C	0.2871595	-3.7732906	-1.1502771
C	1.5662053	-4.3287130	-1.0758498
C	2.4675636	-3.8556443	-0.1175490
C	2.0919589	-2.8360180	0.7539651
C	0.8099332	-2.2597549	0.6911371
H	2.8024115	-2.4625859	1.4873814
H	3.4651306	-4.2809656	-0.0513075
H	1.8570389	-5.1236361	-1.7564607
H	-0.4201426	-4.1330935	-1.8932346
H	-1.0789682	-2.3226200	-0.3594268

$K(PPh_2)_2$: radical
47
Energy = -2209.945128981

P	0.3777061	-0.7745939	-0.9833383
C	-0.7141630	-2.2077987	-0.8136670
C	-0.2730444	-3.5259883	-0.5790131
C	-1.1785072	-4.5822617	-0.5049445
C	-2.5500635	-4.3570064	-0.6615131
C	-3.0022206	-3.0567642	-0.9025022
C	-2.1007648	-1.9989457	-0.9801402
H	-2.4716541	-0.9933038	-1.1577624
K	0.7268799	2.2379962	-1.8767540
P	-0.2912714	0.2732621	1.5258262
C	0.5382196	1.8576012	1.2299076
C	-0.0721893	3.1354231	1.2211346
C	0.6732704	4.2966389	1.0135726

C	2.0605159	4.2322642	0.8128069
C	2.6904662	2.9816530	0.8442540
C	1.9469230	1.8178147	1.0434877
H	2.4531519	0.8563918	1.0618978
H	3.7670308	2.9109135	0.7077914
H	2.6375515	5.1383804	0.6530562
H	0.1703705	5.2608118	1.0192593
H	-1.1403378	3.2166736	1.3983988
C	-2.0389596	0.5676978	1.1403502
C	-2.9744212	-0.2594154	1.8005080
C	-4.3260296	-0.2397019	1.4707431
C	-4.7957110	0.6107846	0.4637809
C	-3.8894806	1.4397141	-0.2039201
C	-2.5362988	1.4152935	0.1263152
H	-1.8544555	2.0615579	-0.4192663
H	-4.2379234	2.1004049	-0.9943938
H	-5.8501347	0.6275615	0.2033108
H	-5.0167468	-0.8944539	1.9957954
H	-2.6191088	-0.9374844	2.5732439
H	-4.0650588	-2.8613642	-1.0206710
H	-3.2538176	-5.1822861	-0.5972462
H	-0.8102879	-5.5896384	-0.3251199
H	0.7883134	-3.7243910	-0.4645077
C	2.0251743	-1.3144094	-0.4493756
C	3.1428005	-0.9006472	-1.2040088
C	4.4437829	-1.1503865	-0.7720697
C	4.6653368	-1.8374246	0.4264516
C	3.5704734	-2.2604644	1.1863020
C	2.2693291	-1.9960808	0.7626195
H	1.4284215	-2.2937043	1.3809220
H	3.7323644	-2.7868811	2.1238499
H	5.6780675	-2.0406158	0.7631636
H	5.2859816	-0.8199517	-1.3748831
H	2.9812350	-0.3853646	-2.1499865

$K(PPh_2)_2^+$: K^+ cation complex
47
Energy = -2209.849351977

P	0.4796410	-0.5184321	-0.5476853
C	-0.6691168	-1.9364215	-0.6592335
C	-0.4785998	-3.1271485	0.0560597
C	-1.4306839	-4.1431091	-0.0119069
C	-2.5797406	-3.9785350	-0.7895463
C	-2.7721671	-2.7975304	-1.5083952
C	-1.8187166	-1.7827833	-1.4483539
H	-1.9718010	-0.8613685	-2.0032541
K	0.7920641	2.3497470	-2.1745108
P	-0.1279822	0.2605832	1.4760457
C	0.5396711	1.9625499	1.2779923
C	-0.2129981	3.1177204	1.5485228
C	0.3603535	4.3855857	1.4249913
C	1.6967186	4.5256323	1.0430256
C	2.4668702	3.3842767	0.7998180
C	1.8945698	2.1154373	0.9174055
H	2.5096913	1.2351804	0.7421971
H	3.5145819	3.4792549	0.5279030
H	2.1385889	5.5130473	0.9508425
H	-0.2411471	5.2659901	1.6319244
H	-1.2509742	3.0282922	1.8539455
C	-1.9081343	0.4115278	1.0677747
C	-2.7638977	-0.5138384	1.6834740
C	-4.1166172	-0.5617205	1.3466910
C	-4.6331590	0.3216432	0.3989658
C	-3.7926399	1.2545663	-0.2141819
C	-2.4398097	1.2932480	0.1141163
H	-1.7947011	2.0241757	-0.3659352
H	-4.1915014	1.9461539	-0.9509148
H	-5.6869724	0.2874647	0.1391006
H	-4.7656872	-1.2880401	1.8265070
H	-2.3619907	-1.2064662	2.4177970
H	-3.6627292	-2.6664325	-2.1159631
H	-3.3210572	-4.7708493	-0.8369928
H	-1.2773556	-5.0628875	0.5452650
H	0.4118781	-3.2587823	0.6634519
C	2.1091066	-1.2929639	-0.2145752
C	2.9318481	-1.5301558	-1.3284350
C	4.1882908	-2.1129329	-1.1704869
C	4.6450388	-2.4516294	0.1053438
C	3.8390816	-2.2109728	1.2193911
C	2.5767512	-1.6374321	1.0620984
H	1.9542847	-1.4542161	1.9337469
H	4.1908286	-2.4715583	2.2134484
H	5.6271192	-2.8980311	0.2304167
H	4.8121455	-2.2961752	-2.0405110
H	2.5817724	-1.2641541	-2.3237146

$KPPh_2$ monomer

24

Energy = -1404.962675497

P	-0.1637606	0.6340507	1.5000609
K	0.6713660	1.9977525	-1.2339979
C	-1.6468932	0.0151796	0.6225224
C	-2.6981259	0.9242339	0.3562418
C	-1.8810932	-1.3392145	0.2901002
C	-3.9033010	0.5099350	-0.2116136
H	-2.5594997	1.9744517	0.6105553
C	-3.0794766	-1.7519949	-0.2890380
H	-1.1182688	-2.0806448	0.5119902
C	-4.1020315	-0.8323109	-0.5470968
H	-4.6836517	1.2416997	-0.4089615
H	-3.2245663	-2.8043665	-0.5234148
H	-5.0361196	-1.1573264	-0.9968792
C	1.2254947	-0.2618616	0.7353387
C	2.4999161	-0.1132308	1.3484453
C	1.2071446	-0.9740257	-0.4931354
C	3.6569007	-0.6349966	0.7813807
H	2.5645075	0.4277094	2.2912752
C	2.3712059	-1.5012728	-1.0567860
H	0.2614570	-1.1304457	-1.0070971
C	3.6090838	-1.3402811	-0.4300000
H	4.6067642	-0.5058659	1.2958628
H	2.3038919	-2.0487630	-1.9949225
H	4.5108427	-1.7642804	-0.8621257

$PMe_3H^+BF_4^-$: contact ion pair

19

Energy = -886.5172918428

P	-0.2398936	-0.1258389	-0.6032392
C	-1.3387655	-1.1016491	0.4409558
H	-2.3687355	-0.7645035	0.3005712
H	-1.2604508	-2.1557743	0.1627303
H	-1.0530040	-0.9782605	1.4889812
C	-0.3988874	1.6182545	-0.1945254
H	0.2453577	2.1894289	-0.8655560
H	-1.4394656	1.9239743	-0.3301654
H	-0.1020753	1.7752458	0.8459536
C	1.4521803	-0.6976132	-0.4064087
H	1.5082968	-1.7493616	-0.6969000
H	2.0954157	-0.1045541	-1.0580479
H	1.7478631	-0.5830012	0.6402454
H	-0.6404476	-0.3054379	-1.9298785
B	0.8360896	0.4607116	-3.8973046
F	1.2873076	1.3089479	-2.8387073
F	1.4165745	0.8538668	-5.1054053
F	1.1918909	-0.8724406	-3.5770796
F	-0.5778521	0.5530327	-3.9654294

$(PPh_2)_2$

46

Energy = -1609.982299811

P	0.6713061	0.4711531	0.8568655
C	1.3254875	-1.2371153	0.6982949
C	2.2178853	-1.6243263	-0.3126147
C	2.6142837	-2.9555870	-0.4277805
C	2.1195259	-3.9148239	0.4602334
C	1.2320577	-3.5370835	1.4688056
C	0.8401846	-2.2040932	1.5900252
H	0.1418558	-1.9107567	2.3691904
H	0.8433113	-4.2792213	2.1603539
H	2.4262778	-4.9527713	0.3652861
H	3.3033667	-3.2472433	-1.2158746
H	2.5957537	-0.8815512	-1.0092703
C	2.0849660	1.4721752	0.2432270
C	3.3243001	1.3501422	0.8955709
C	4.4013484	2.1550052	0.5312841
C	4.2561538	3.1082562	-0.4810513
C	3.0279860	3.2446870	-1.1283553
C	1.9509110	2.4311647	-0.7705940
H	1.0069764	2.5386797	-1.2992356
H	2.9064502	3.9790235	-1.9200048
H	5.0955166	3.7375465	-0.7630118
H	5.3551055	2.0397215	1.0390482
H	3.4464750	0.6132459	1.6857040
P	-0.6265232	0.3980801	-0.9736301
C	-1.5621155	-1.1052991	-0.4880425
C	-1.2619787	-2.3010503	-1.1557731
C	-1.8757414	-3.4948749	-0.7775657
C	-2.8020129	-3.5034421	0.2662067
C	-3.1126632	-2.3143659	0.9317896
C	-2.4946093	-1.1217168	0.5599345
H	-2.7300975	-0.1999776	1.0841335
H	-3.8315388	-2.3183923	1.7467657

H	-3.2817180	-4.4326115	0.5610969
H	-1.6293583	-4.4168022	-1.2966922
H	-0.5335355	-2.2951316	-1.9620375
C	-1.8364863	1.7296107	-0.5988835
C	-1.5263375	2.8306700	0.2115423
C	-2.4413159	3.8721696	0.3788192
C	-3.6816350	3.8258769	-0.2578650
C	-4.0018145	2.7325257	-1.0682316
C	-3.0853749	1.6982170	-1.2436709
H	-3.3425959	0.8534819	-1.8781105
H	-4.9666744	2.6867167	-1.5659519
H	-4.3955216	4.6335902	-0.1235638
H	-2.1849245	4.7150117	1.0148696
H	-0.5722366	2.8747267	0.7310297

PMe_3H^+
14
Energy = -461.6566914888

P	0.0001093	-0.0001007	-0.3209478
C	-1.3302927	-1.0747205	0.2314788
H	-2.2833067	-0.6963292	-0.1465669
H	-1.1603381	-2.0862277	-0.1458201
H	-1.3478148	-1.0883623	1.3247768
C	-0.2656873	1.6893719	0.2312227
H	0.5388569	2.3254620	-0.1462368
H	-1.2263165	2.0478292	-0.1468458
H	-0.2694648	1.7114787	1.3245236
C	1.5958216	-0.6144883	0.2321817
H	1.7448096	-1.6292704	-0.1451648
H	2.3868044	0.0383438	-0.1453017
H	1.6161540	-0.6222348	1.3254468
H	0.0004972	-0.0004796	-1.7174936

PMe_3
13
Energy = -461.2159485900

P	-0.6881056	0.0058659	0.1106211
C	0.1787003	1.6307816	-0.0974585
H	-0.2484001	2.1648993	-0.9523554
H	0.0244420	2.2431708	0.7968048
H	1.2554184	1.4989182	-0.2595205
C	-0.0423066	-0.8794450	-1.3840480
H	-0.3429769	-1.9313005	-1.3428376
H	-0.4774736	-0.4371096	-2.2859654
H	1.0510662	-0.8221574	-1.4491043
C	0.3947269	-0.7546589	1.4083012
H	0.2482239	-0.2306795	2.3582984
H	0.1112232	-1.8023702	1.5514328
H	1.4550619	-0.7053833	1.1322541

*t*BuPMe$_3^+$ phosphonium
26
Energy = -619.0258004585

C	-1.0837519	-0.0000392	-0.0000304
C	-1.5796551	1.0999928	0.9600560
C	-1.5799947	0.2816600	-1.4325697
C	-1.5799442	-1.3814138	0.4724627
H	-1.2530769	2.0958329	0.6467596
H	-2.6742866	1.0900240	0.9524838
H	-1.2518339	0.9251051	1.9889320
H	-2.6746779	0.2793346	-1.4201206
H	-1.2528635	1.2605124	-1.7950384
H	-1.2530475	-0.4870421	-2.1388180
H	-2.6745940	-1.3696020	0.4679971
H	-1.2526547	-2.1848896	-0.1937814
H	-1.2531241	-1.6083016	1.4913925
P	0.7572796	-0.0000407	-0.0001330
C	1.3962974	-1.2709920	-1.1100611
H	1.0564615	-1.0831939	-2.1311300
H	2.4890985	-1.2406802	-1.0844739
H	1.0574611	-2.2575632	-0.7857250
C	1.3958224	1.5968762	-0.5455712
H	2.4885819	1.5595866	-0.5327813
H	1.0564070	1.8098534	-1.5617685
H	1.0563610	2.3867266	0.1283359
C	1.3959914	-0.3260589	1.6555681
H	2.4888337	-0.3183602	1.6171439
H	1.0562605	0.4473264	2.3482017
H	1.0565909	-1.3046736	2.0026841

*t*Bu$^+$ cation $C(CH_3)_3^+$
13
Energy = -157.7131521247

C	0.0000375	-0.0000331	0.0000817
C	0.9237244	-1.1293720	0.0010744
C	-1.4398475	-0.2353552	-0.0008044

C	0.5163396	1.3646906	-0.0001379
H	0.6768038	-1.7738971	0.8609465
H	0.6861806	-1.7636185	-0.8694053
H	1.9762524	-0.8515892	0.0073751
H	-1.8750991	0.3005206	-0.8604430
H	-1.7254698	-1.2858384	-0.0065351
H	-1.8701201	0.2873023	0.8699311
H	1.1906667	1.4743414	-0.8658461
H	-0.2504194	2.1374680	-0.0006990
H	1.1913300	1.4753916	0.8647851

TS1a⁺ TS for P..C addition of **1⁺** and PMe_3

59

Energy = -1460.234620468

C	1.0493335	3.3234615	-2.7175002
C	0.1510369	2.2856818	-2.9898199
C	1.9423296	3.2393836	-1.6458462
H	1.0562955	4.2043970	-3.3525244
C	0.1231393	1.1419901	-2.1830067
H	-0.5265163	2.3667630	-3.8343939
C	1.9217537	2.0980934	-0.8415403
H	2.6407105	4.0476637	-1.4492753
C	1.0039939	1.0668838	-1.1144710
H	-0.5689253	0.3333549	-2.4012664
C	2.7276861	1.7178017	0.3249660
C	1.1357347	-0.0439878	-0.0692802
C	2.3014861	0.4554530	0.7791140
C	3.7673862	2.3791980	0.9809288
C	-0.1518758	-0.2957877	0.6827553
N	1.4443200	-1.3396112	-0.7213888
C	2.8949383	-0.1631978	1.8690277
C	4.3691221	1.7585679	2.0788921
H	4.1078782	3.3550507	0.6468743
C	-0.5121569	-1.6384159	0.4080661
C	-0.8976489	0.5060177	1.4934776
N	0.4790582	-2.1534626	-0.4074597
C	3.9415532	0.5009263	2.5194834
H	2.5609823	-1.1373302	2.2149244
H	5.1811571	2.2588666	2.5982335
C	-1.6466677	-2.2179798	1.0274482
C	-2.1751811	0.0101422	1.9680835
H	-0.5783612	1.5121323	1.7444892
C	0.5338511	-3.5674098	-0.9541746
H	4.4247299	0.0368695	3.3739359
C	-2.4285795	-1.4142185	1.8152501
H	-1.8866937	-3.2690478	0.9298241
H	-2.5837926	0.4772599	2.8588562
C	1.7505145	-3.6941780	-1.8713974
C	0.6750301	-4.5248302	0.2394786
C	-0.7538293	-3.8098798	-1.7559374
H	-3.2872642	-1.8415878	2.3231383
H	1.6904393	-2.9993630	-2.7119205
H	2.6800429	-3.5059366	-1.3311606
H	1.7640604	-4.7175653	-2.2573703
H	-0.1839379	-4.4884795	0.9117383
H	0.7632169	-5.5440412	-0.1461710
H	1.5793840	-4.2910870	0.8085553
H	-0.7042516	-4.8113441	-2.1915077
H	-1.6508762	-3.7527047	-1.1368130
H	-0.8381399	-3.0843144	-2.5705860
P	-3.6361059	1.0329622	0.5297272
C	-5.3900722	1.3861671	0.9000520
H	-5.8866583	0.4620629	1.2084577
H	-5.4476114	2.1105016	1.7167327
H	-5.8950060	1.7916418	0.0162397
C	-3.6287791	-0.0668119	-0.9180378
H	-2.5929345	-0.2891592	-1.1868616
H	-4.1347654	-1.0030366	-0.6693552
H	-4.1324147	0.4066388	-1.7660380
C	-2.8901879	2.5961678	-0.0324894
H	-2.9083742	3.3260575	0.7812026
H	-1.8536952	2.4136280	-0.3306501
H	-3.4441225	2.9934137	-0.8886993

TS1m⁺ TS for *t*Bu⁺ transfer from **1⁺** to PMe_3

59

Energy = -1460.207369706

C	-0.7479769	-4.5430030	3.0041731
C	-1.6331587	-3.4626474	2.9207740
C	0.2707217	-4.7152415	2.0630035
H	-0.8571136	-5.2612246	3.8113435
C	-1.5144761	-2.5244199	1.8888909
H	-2.4205000	-3.3523164	3.6599865
C	0.3944027	-3.7836325	1.0315825
H	0.9490087	-5.5602334	2.1361396

C	-0.5022213	-2.7003936	0.9577805
H	-2.2055373	-1.6887760	1.8220182
C	1.3312944	-3.7021346	-0.0941260
C	-0.1632842	-1.8270091	-0.2460340
C	1.0166327	-2.5683560	-0.8676116
C	2.3860760	-4.5309225	-0.4785667
C	-1.2880350	-1.4336198	-1.1555685
N	0.3315109	-0.4828296	0.2587588
C	1.7308059	-2.2414110	-2.0103961
C	3.1090969	-4.2066610	-1.6298692
H	2.6426194	-5.4129117	0.1009368
C	-1.3632856	-0.0369017	-1.1261145
C	-2.1712774	-2.1605479	-1.9388194
N	-0.3449478	0.4358111	-0.2447040
C	2.7876294	-3.0769662	-2.3903778
H	1.4785442	-1.3674129	-2.6043886
H	3.9313648	-4.8436191	-1.9419317
C	-2.2969775	0.6908959	-1.8576566
C	-3.1199304	-1.4476646	-2.6824791
H	-2.1342556	-3.2443366	-1.9786027
C	0.1028720	2.4929572	0.2370938
H	3.3598433	-2.8478354	-3.2839889
C	-3.1825747	-0.0470210	-2.6438778
H	-2.3405755	1.7731559	-1.8246934
H	-3.8242954	-1.9910092	-3.3044778
C	1.2208404	2.1740661	1.1858538
C	0.4336062	2.8556268	-1.1998196
C	-1.2790688	2.7511083	0.8076560
H	-3.9313249	0.4705319	-3.2343675
H	0.9862236	2.5187546	2.1950903
H	1.3768491	1.0871742	1.2391271
H	2.1592562	2.6194100	0.8492739
H	-0.3125050	3.5378449	-1.6132882
H	1.4181488	3.3233695	-1.2529205
H	0.4712212	1.9717092	-1.8439845
H	-1.1988204	3.3352633	1.7258235
H	-1.9106072	3.2888745	0.0976655
H	-1.7802800	1.8170856	1.0788154
P	0.6445089	5.3318127	0.7527214
C	2.2536318	5.9141558	0.0744600
H	3.0645211	5.2937055	0.4687112
H	2.2462941	5.8199456	-1.0159168
H	2.4446786	6.9599195	0.3392105
C	0.7794411	5.7936369	2.5298063
H	-0.1713992	5.5963026	3.0346585
H	1.5553160	5.1870734	3.0069934
H	1.0318517	6.8531438	2.6488059
C	-0.5322544	6.5914429	0.1058644
H	-0.6183260	6.4843543	-0.9798346
H	-1.5201064	6.4275250	0.5473290
H	-0.2007797	7.6092524	0.3395794

TS1b$^+$ 1,2-*t*Bu-shift of cation **1**$^+$

46

Energy = -998.9725514649

C	-3.2930475	0.4540018	3.2314115
C	-2.0197555	0.8004530	3.6975970
C	-3.5046132	0.1322077	1.8880607
H	-4.1297152	0.4399265	3.9236781
C	-0.9247835	0.8272141	2.8260617
H	-1.8802719	1.0533182	4.7442708
C	-2.4173846	0.1618728	1.0135582
H	-4.4980975	-0.1304639	1.5361832
C	-1.1385261	0.5100216	1.4925497
H	0.0629782	1.0971115	3.1890637
C	-2.3273721	-0.0948432	-0.4281551
C	-0.1225042	0.4399441	0.3648700
C	-0.9953789	0.1031081	-0.8404327
C	-3.2973549	-0.4507406	-1.3673990
C	0.8648144	1.5490305	0.2000956
N	0.7865219	-0.7695713	0.5863073
C	-0.6155928	-0.0350167	-2.1682623
C	-2.9174634	-0.5995314	-2.7042380
H	-4.3308466	-0.6050182	-1.0710445
C	2.1359201	0.9656521	0.2798943
C	0.7388460	2.9149678	-0.0039893
N	2.0020759	-0.4309433	0.5132997
C	-1.5933845	-0.3892539	-3.1059019
H	0.4095049	0.1467408	-2.4816713
H	-3.6631849	-0.8719785	-3.4452218
C	3.3138571	1.6972140	0.1561206
C	1.9140748	3.6677154	-0.1303292
H	-0.2335662	3.3933774	-0.0651342
C	2.1160621	-3.0133327	-0.5226743
H	-1.3246562	-0.4928612	-4.1527450
C	3.1810370	3.0708234	-0.0535724

H	4.2866461	1.2212955	0.2258576
H	1.8432643	4.7393156	-0.2901988
C	0.7903774	-3.2736294	-1.0918756
C	3.1887872	-2.5227185	-1.3975492
C	2.4275597	-3.4569411	0.8360087
H	4.0680387	3.6880733	-0.1545918
H	0.0217673	-3.4118130	-0.3341330
H	0.5004614	-2.5266286	-1.8349025
H	0.9049395	-4.2232960	-1.6507414
H	4.0284333	-2.1002545	-0.8476608
H	3.5544570	-3.4227844	-1.9286037
H	2.8206240	-1.8480277	-2.1733169
H	2.8201843	-4.4868686	0.7070516
H	3.2280419	-2.8724261	1.2907966
H	1.5434148	-3.5226257	1.4708752

TS1$^+$ TS for direct N..P adition of **1$^+$** and PMe_3

59

Energy = -1460.244926139

C	-4.3343711	-0.8455185	1.0135907
C	-3.3574815	-1.2740712	1.9158388
C	-4.0141748	0.0262932	-0.0345679
H	-5.3633150	-1.1653489	1.1476917
C	-2.0218123	-0.8779072	1.7562149
H	-3.6365143	-1.9056389	2.7536359
C	-2.6897264	0.4312847	-0.1864125
H	-4.7921305	0.3951577	-0.6962517
C	-1.6940522	-0.0712943	0.6767627
H	-1.2649538	-1.1851688	2.4708938
C	-2.0798523	1.4348739	-1.0685765
C	-0.3403134	0.4744225	0.2944955
C	-0.7187142	1.5614988	-0.7324631
C	-2.6400907	2.2672806	-2.0362969
C	0.5992153	0.8946143	1.3853015
N	0.5301984	-0.4724381	-0.5506048
C	0.0817667	2.5278336	-1.3169698
C	-1.8267631	3.2260045	-2.6499605
H	-3.6908128	2.1892486	-2.3000004
C	1.8910485	0.5402603	0.9851775
C	0.3792981	1.5548599	2.5838272
N	1.7802963	-0.1116490	-0.2720404
C	-0.4843832	3.3660864	-2.2867524
H	1.1171260	2.6546713	-1.0185360
H	-2.2490815	3.8837463	-3.4036657
C	3.0040446	0.8198615	1.7759329
C	1.4866749	1.8451058	3.3894622
H	-0.6232280	1.8370491	2.8901262
C	2.8866470	-0.2582502	-1.2913461
H	0.1232311	4.1355514	-2.7529884
C	2.7754153	1.4791780	2.9876926
H	4.0085818	0.5326850	1.4930688
H	1.3442349	2.3530947	4.3377969
C	2.2621959	-0.5836846	-2.6505961
C	3.6020979	1.1007557	-1.4155593
C	3.8924879	-1.3295307	-0.8447476
H	3.6207379	1.7007657	3.6314654
H	1.7025846	-1.5185218	-2.6492120
H	1.5828893	0.2143863	-2.9611775
H	3.0707329	-0.6649147	-3.3818351
H	4.0536594	1.4282006	-0.4783364
H	4.3996162	0.9911186	-2.1554981
H	2.9156943	1.8735439	-1.7683542
H	4.6920655	-1.3895182	-1.5884803
H	4.3461144	-1.0790297	0.1171273
H	3.4299500	-2.3124211	-0.7631812
P	-0.0971382	-2.5925921	-0.0788750
C	-1.4696593	-2.6604232	-1.2723650
H	-2.3863453	-2.2963123	-0.8031260
H	-1.2299908	-2.0260933	-2.1292310
H	-1.6174136	-3.6917456	-1.6077291
C	-0.6075231	-3.7005813	1.3064958
H	0.1216804	-3.6375957	2.1182300
H	-1.5913822	-3.4165593	1.6782655
H	-0.6400177	-4.7299103	0.9313674
C	1.2098780	-3.6257109	-0.8287339
H	1.6109835	-3.1900441	-1.7426920
H	2.0181805	-3.7756508	-0.1085556
H	0.7724623	-4.6009796	-1.0669808

TS2m$^+$ TS for *t*Bu$^+$ transfer from **2$^+$** to PMe_3 59

Energy = -1460.210374701

C	-0.9464297	-2.6578112	3.0156867
C	-0.9719045	-1.3083373	3.3856215
C	-1.1165163	-3.0430547	1.6831528

H	-0.7947478	-3.4171722	3.7769650
C	-1.1684055	-0.3108046	2.4237963
H	-0.8420947	-1.0325992	4.4275235
C	-1.3083198	-2.0528295	0.7186777
H	-1.0970183	-4.0935127	1.4082142
C	-1.3320896	-0.6975694	1.1018946
H	-1.1934169	0.7360761	2.7128466
C	-1.4859615	-2.1381411	-0.7346640
C	-1.5481599	0.1855019	-0.1213274
C	-1.6090051	-0.8341872	-1.2552166
C	-1.5232117	-3.2345958	-1.5968212
C	-2.6792433	1.1693788	-0.0610177
N	-0.3575842	1.1266424	-0.2956598
C	-1.7682637	-0.6021088	-2.6133435
C	-1.6798962	-3.0057931	-2.9667226
H	-1.4317405	-4.2474647	-1.2158244
C	-2.1136359	2.4432561	-0.1972195
C	-4.0495621	1.0264952	0.0824585
N	-0.7068492	2.3269851	-0.3237445
C	-1.8007780	-1.7067708	-3.4725497
H	-1.8672027	0.4059755	-3.0058846
H	-1.7102658	-3.8493731	-3.6497798
C	-2.8670521	3.6139641	-0.1958069
C	-4.8248486	2.1944438	0.0878864
H	-4.5144407	0.0515860	0.1886204
C	1.8842048	0.7611749	-0.1503045
H	-1.9240877	-1.5534980	-4.5401390
C	-4.2466794	3.4654880	-0.0489505
H	-2.4008805	4.5874688	-0.3039352
H	-5.9015442	2.1138314	0.2014313
C	1.7534590	-0.7150511	-0.4045467
C	2.1163396	1.6788010	-1.3105214
C	1.9499095	1.3482432	1.2339253
H	-4.8837643	4.3438448	-0.0402542
H	1.7613350	-1.2904224	0.5220538
H	0.8282697	-0.9210615	-0.9486018
H	2.5648914	-1.0545699	-1.0520987
H	3.1752784	1.9505031	-1.3569866
H	1.8362544	1.2105729	-2.2562258
H	1.5540800	2.6074930	-1.1719260
H	1.9685600	0.5738995	2.0017864
H	2.8345007	1.9822271	1.3281989
H	1.0924160	2.0057699	1.4053251
P	4.7599305	0.1660645	0.2326298
C	5.5759584	-0.3053580	-1.3488273
H	5.1242310	-1.2263288	-1.7313469
H	5.4221994	0.4857971	-2.0895089
H	6.6515623	-0.4661464	-1.2167732
C	5.3120997	-1.1691998	1.3736442
H	4.9762014	-0.9412694	2.3897650
H	4.8629192	-2.1186372	1.0664398
H	6.4028401	-1.2732455	1.3719028
C	5.7664516	1.6039792	0.7870987
H	5.6289607	2.4381830	0.0918193
H	5.4289888	1.9237907	1.7781166
H	6.8321567	1.3547619	0.8367743

TS2$^+$ direct P..C addition of **2$^+$** and PMe_3

59

Energy = -1460.249293091

C	0.3839228	3.5194607	-1.8753498
C	-0.2281629	2.4160134	-2.4806431
C	1.1278714	3.3762534	-0.7009217
H	0.2841499	4.5008287	-2.3292598
C	-0.1158783	1.1418865	-1.9120605
H	-0.7924149	2.5488926	-3.3985177
C	1.2515740	2.1060302	-0.1348522
H	1.6044998	4.2391390	-0.2451346
C	0.6223756	1.0062636	-0.7461067
H	-0.5828290	0.2828917	-2.3860176
C	1.9811139	1.6373053	1.0498108
C	0.8500671	-0.2496729	0.0925937
C	1.8010456	0.2459995	1.1772178
C	2.7727006	2.3224183	1.9726204
C	-0.4483292	-0.7965968	0.6417525
N	1.3088831	-1.4423937	-0.7010577
C	2.3808638	-0.4713007	2.2126072
C	3.3746702	1.6004852	3.0067583
H	2.9233165	3.3949310	1.8915488
C	-0.5796536	-2.1189433	0.1239715
C	-1.3755400	-0.2530198	1.4760006
N	0.4752850	-2.4548912	-0.6381610
C	3.1830612	0.2196963	3.1282196
H	2.2233515	-1.5410022	2.3150896
H	3.9984473	2.1186973	3.7288928
C	-1.6844746	-2.9313436	0.4859604

C	-2.5784023	-1.0080151	1.7347908
H	-1.2485746	0.7313834	1.9153613
C	2.5994855	-1.6332007	-1.4443181
H	3.6576255	-0.3205566	3.9415006
C	-2.6427117	-2.3859301	1.2986820
H	-1.7405600	-3.9629822	0.1536245
H	-3.1625844	-0.7359526	2.6064363
C	3.3318316	-0.2985977	-1.5971567
C	3.4588516	-2.6284619	-0.6459651
C	2.2566286	-2.2036520	-2.8288659
H	-3.4898545	-2.9852052	1.6165935
H	2.7529757	0.4169117	-2.1852102
H	3.5802657	0.1449043	-0.6303019
H	4.2684009	-0.4953436	-2.1258710
H	2.9144276	-3.5648970	-0.4969911
H	4.3770822	-2.8391708	-1.2021014
H	3.7315114	-2.2119134	0.3278559
H	3.1841626	-2.3560566	-3.3879764
H	1.7374874	-3.1598601	-2.7368742
H	1.6244164	-1.5052617	-3.3860882
P	-4.0908923	0.1341641	0.2500638
C	-5.9092109	0.2516742	0.4134170
H	-6.3290117	-0.7537821	0.5042906
H	-6.1554549	0.8216181	1.3130601
H	-6.3416209	0.7477367	-0.4628174
C	-3.7875316	-0.7163620	-1.3305118
H	-2.7101225	-0.7800372	-1.5039802
H	-4.1935398	-1.7301319	-1.2860913
H	-4.2556030	-0.1728124	-2.1572582
C	-3.4908980	1.8395340	0.0019337
H	-3.6826923	2.4313527	0.9008735
H	-2.4149959	1.8203431	-0.1935535
H	-3.9998053	2.3009189	-0.8506591

TS3b$^+$ TS for **3p** protonation with PMe_3H^+
72
Energy = -1921.466497907

C	-1.0296474	3.4276393	2.8741865
C	-0.8204544	2.1862351	3.4878747
C	-1.0275203	3.5449606	1.4812808
H	-1.2029651	4.3074389	3.4877651
C	-0.5933238	1.0401572	2.7167450
H	-0.8374406	2.1148751	4.5720618
C	-0.8099699	2.3980186	0.7127625
H	-1.2029381	4.5098501	1.0123026
C	-0.5825015	1.1548662	1.3345630
H	-0.4419128	0.0708653	3.1838588
C	-0.8249544	2.1959031	-0.7448752
C	-0.3663239	0.0484617	0.3049449
C	-0.5949702	0.8311044	-1.0068730
C	-1.0467570	3.0914044	-1.7955383
C	1.0288217	-0.5650062	0.3232142
N	-1.2542245	-1.1086056	0.4993938
C	-0.5901936	0.3494161	-2.3096782
C	-1.0354871	2.6040075	-3.1063857
H	-1.2259699	4.1467679	-1.6058236
C	0.8685043	-1.9877216	0.2185170
C	2.2518145	0.0360223	0.3660443
N	-0.4485602	-2.2543429	0.2753263
C	-0.8124633	1.2446855	-3.3626827
H	-0.4180560	-0.7053748	-2.5076642
H	-1.2070931	3.2852480	-3.9350762
C	2.0468252	-2.7867434	0.1149977
C	3.4358546	-0.7636364	0.2842515
H	2.3145617	1.1195341	0.4521288
C	-1.2213128	-3.5116174	0.1393917
H	-0.8131814	0.8857687	-4.3883334
C	3.2822003	-2.1701570	0.1470995
H	2.0092903	-3.8623344	0.0252775
H	-2.7971781	-0.5832469	-0.1612706
C	-2.2024416	-3.6025005	1.3221996
C	-1.9949375	-3.4494957	-1.1914618
C	-0.3259379	-4.7533930	0.1440515
H	4.1632293	-2.8014757	0.0736370
H	-1.6541178	-3.6453695	2.2685422
H	-2.8565673	-2.7299963	1.3465984
H	-2.8041454	-4.5115547	1.2219889
H	-1.2996007	-3.4137815	-2.0363293
H	-2.6290181	-4.3354525	-1.2978974
H	-2.6225005	-2.5557160	-1.2207713
H	-0.9747925	-5.6338063	0.1135742
H	0.3253530	-4.7903700	-0.7328996
H	0.2797650	-4.8109389	1.0527075
P	4.9995313	0.0062785	0.2946003
C	6.3265625	-1.2171220	0.3261830
H	6.2330661	-1.8459422	1.2146989

H	6.2841987	-1.8390078	-0.5711882
H	7.2856222	-0.6928192	0.3526658
C	5.2003377	1.0923416	1.7338919
H	4.3904560	1.8271984	1.7452228
H	5.1503185	0.4930027	2.6462001
H	6.1587428	1.6171578	1.6839717
C	5.2721788	1.0697535	-1.1534990
H	5.2535971	0.4552972	-2.0571552
H	4.4665560	1.8075020	-1.2086052
H	6.2321846	1.5888907	-1.0761970
P	-4.0742952	0.1202287	-0.3356300
C	-4.1811655	0.8904870	-1.9652805
H	-5.1259366	1.4312795	-2.0690822
H	-3.3412621	1.5804994	-2.0870546
H	-4.1168129	0.1158211	-2.7336231
C	-5.4399048	-1.0538910	-0.1473851
H	-5.3693452	-1.8189753	-0.9251887
H	-5.3690660	-1.5328406	0.8328780
H	-6.4011990	-0.5393595	-0.2313996
C	-4.2288798	1.3917598	0.9385646
H	-5.1875241	1.9092551	0.8436630
H	-4.1594097	0.9184876	1.9213163
H	-3.4078299	2.1055403	0.8358716

TS3p⁺ deprotonation TS of **3a⁺** with PMe_3

72

Energy = -1921.438040190

C	2.4536738	-3.4889407	-2.8364135
C	2.5213869	-3.7415334	-1.4609393
C	2.1897378	-2.2031009	-3.3162449
H	2.6154442	-4.3016038	-3.5390179
C	2.3129855	-2.7109468	-0.5364520
H	2.7402449	-4.7461336	-1.1099787
C	1.9966060	-1.1707312	-2.3953180
H	2.1457458	-2.0150399	-4.3857130
C	2.0543404	-1.4338989	-1.0123126
H	2.3619356	-2.9043589	0.5316289
C	1.7535645	0.2665513	-2.5895055
C	1.7191612	-0.1781161	-0.2144034
C	1.6607408	0.8768039	-1.3242184
C	1.6670705	1.0426333	-3.7483953
C	0.3678909	-0.3066720	0.4838009
N	2.6549190	0.1833052	0.8626843
C	1.5392116	2.2526341	-1.1976011
C	1.5134786	2.4267192	-3.6212369
H	1.7370283	0.5885086	-4.7331852
C	0.5693909	0.1484792	1.8328014
C	-0.8141173	-0.7670854	0.0187657
N	1.8950469	0.3925545	1.9613827
C	1.4633908	3.0307578	-2.3594840
H	1.5264822	2.7181729	-0.2159438
H	1.4512394	3.0437126	-4.5130743
C	-0.5632295	0.1730984	2.7104367
C	-2.0230750	-0.6690667	0.8603890
H	-0.8891928	-1.1303249	-1.0036182
C	2.6925356	0.8633819	3.1505218
H	1.3699827	4.1103284	-2.2832654
C	-1.7784361	-0.2454934	2.2486105
H	-0.4919252	0.5098008	3.7341864
H	-2.6592300	0.5471726	0.2112521
C	3.7660876	-0.2010749	3.4256906
C	3.3381968	2.2019300	2.7581750
C	1.8306181	1.0561308	4.3975899
H	-2.6218377	-0.2089225	2.9330299
H	3.2994003	-1.1532091	3.6992212
H	4.3845712	-0.3515074	2.5402475
H	4.3923870	0.1321054	4.2587965
H	2.5676277	2.9506169	2.5443219
H	3.9486012	2.5633948	3.5912731
H	3.9647898	2.0742546	1.8743920
H	2.4931377	1.3862204	5.2027403
H	1.0702365	1.8290513	4.2559470
H	1.3568243	0.1243133	4.7174192
P	-3.2468126	-1.9179713	0.6144706
C	-4.7590352	-1.4771397	1.4990697
H	-4.5752385	-1.4788464	2.5762503
H	-5.0918488	-0.4829167	1.1895949
H	-5.5385888	-2.2089342	1.2713238
C	-2.7680146	-3.5845755	1.1513318
H	-1.8619348	-3.8807759	0.6149049
H	-2.5566898	-3.5649204	2.2239733
H	-3.5663537	-4.3039122	0.9468041
C	-3.6366562	-2.0248897	-1.1480547
H	-3.9883068	-1.0557739	-1.5110586
H	-2.7485534	-2.3232456	-1.7104848
H	-4.4192759	-2.7720556	-1.3027727

P -2.7637196 2.0469495 -0.5723360
C -2.0711213 1.8994463 -2.2514239
H -2.6598405 1.1779168 -2.8255212
H -2.0887753 2.8652476 -2.7660976
H -1.0389412 1.5459428 -2.1856981
C -4.4292751 2.7624588 -0.7795660
H -4.3791105 3.7125444 -1.3213367
H -5.0587067 2.0652352 -1.3402909
H -4.8791420 2.9319633 0.2026537
C -1.7701585 3.3105703 0.2848360
H -1.7381362 4.2427126 -0.2886815
H -2.2041983 3.5020789 1.2696889
H -0.7559764 2.9268351 0.4150293

TS4a$^+$ TS for P..N addition of **2**$^+$ and PMe_3
59
Energy = -1460.244662790
C 1.8516658 -2.9736745 -2.5912803
C 0.4952348 -2.6345728 -2.6229705
C 2.7469078 -2.2683079 -1.7823334
H 2.2157930 -3.7836482 -3.2161207
C 0.0083207 -1.5919258 -1.8265142
H -0.1809303 -3.1752677 -3.2781707
C 2.2590716 -1.2318940 -0.9860508
H 3.8030792 -2.5215590 -1.7835759
C 0.8876634 -0.9155968 -0.9940881
H -1.0382681 -1.3123525 -1.8836939
C 2.9525918 -0.3002664 -0.0877446
C 0.6042759 0.2409860 -0.0321989
C 2.0074253 0.5733043 0.4799270
C 4.3097402 -0.1503628 0.2038882
C -0.0710240 1.4394576 -0.6550015
N -0.4077369 -0.0677629 1.0527336
C 2.4006699 1.6320129 1.2863514
C 4.7026106 0.8913963 1.0474614
H 5.0505033 -0.8179566 -0.2264166
C -1.3030019 1.6318954 -0.0380400
C 0.3737399 2.3205618 -1.6327764
N -1.5595345 0.6077232 0.9323351
C 3.7607574 1.7815988 1.5764150
H 1.6717019 2.3282518 1.6907399
H 5.7544974 1.0232770 1.2829118
C -2.1238075 2.7157861 -0.3389659
C -0.4461160 3.4031108 -1.9653860
H 1.3363153 2.1825917 -2.1155917
C -0.1878805 -0.9020200 2.2858552
H 4.0886343 2.5976568 2.2129757
C -1.6735031 3.6029878 -1.3186639
H -3.0653075 2.8773558 0.1767226
H -0.1199875 4.1093719 -2.7222325
C 0.6364180 -2.1425117 1.9285985
C 0.5338973 -0.0670284 3.3620742
C -1.5450711 -1.3289714 2.8586878
H -2.2800856 4.4659216 -1.5744432
H 0.7588182 -2.7486879 2.8307361
H 0.1385443 -2.7496913 1.1670996
H 1.6310490 -1.8736522 1.5677687
H 0.0047112 0.8754414 3.5317427
H 0.5411276 -0.6336611 4.2986565
H 1.5657287 0.1460936 3.0834548
H -1.3567412 -1.9262600 3.7545378
H -2.1481120 -0.4640007 3.1411861
H -2.1050725 -1.9548088 2.1617403
P -3.2260548 -0.4345965 -0.0409590
C -3.0340496 -2.2508127 -0.1342184
H -1.9825312 -2.5286648 -0.0187042
H -3.6190711 -2.7297238 0.6540276
H -3.3858789 -2.5993564 -1.1088745
C -3.7329208 0.1211387 -1.7016746
H -3.9213548 1.1965181 -1.6896351
H -2.9375396 -0.0883959 -2.4219508
H -4.6440538 -0.4076219 -2.0027717
C -4.5947835 -0.0507681 1.0795674
H -4.3606123 -0.4261426 2.0782255
H -4.7234442 1.0339712 1.1295166
H -5.5215315 -0.5094005 0.7212640

TS4b$^+$ TS for **4p** protonation with PMe_3H^+
72
Energy = -1921.482073194
C 1.1155843 3.0786877 3.4199546
C 0.2450867 1.9931893 3.5766514
C 1.5362535 3.4790074 2.1490414
H 1.4659116 3.6163632 4.2966351
C -0.2204171 1.2908431 2.4597115
H -0.0712395 1.6981442 4.5733990

C	1.0695109	2.7769890	1.0351580
H	2.2093299	4.3247108	2.0359255
C	0.1927763	1.6891569	1.1975452
H	-0.9021290	0.4541872	2.5726319
C	1.3189933	2.9676168	-0.3996667
C	-0.1706028	1.0772949	-0.1482006
C	0.5841544	2.0059136	-1.1200334
C	2.1406938	3.8783298	-1.0690248
C	0.3277225	-0.3436628	-0.3550115
N	-1.6473219	0.8445870	-0.3064477
C	0.7043514	1.9125749	-2.5010845
C	2.2272521	3.8072269	-2.4612203
H	2.7111898	4.6237416	-0.5211911
C	-0.7340661	-1.0637183	-0.9911232
C	1.5488803	-0.9030434	-0.1154964
N	-1.8659639	-0.3676837	-1.0708170
C	1.5210595	2.8289807	-3.1719160
H	0.1694281	1.1458046	-3.0537774
H	2.8584452	4.5098690	-2.9980947
C	-0.4954445	-2.4093746	-1.3979340
C	1.7835377	-2.2579064	-0.5078982
H	2.3325838	-0.3181333	0.3636840
C	-2.5882907	1.9293286	-0.7003545
H	1.6117738	2.7797804	-4.2534539
C	0.7406600	-2.9751698	-1.1555136
H	-1.2683160	-2.9779894	-1.9072800
H	-2.9692833	-1.2528449	-0.2589983
C	-2.2728800	3.1925178	0.1068773
C	-2.5773331	2.2460679	-2.2085418
C	-3.9943163	1.4383308	-0.3135987
H	0.9126778	-4.0000052	-1.4724202
H	-2.2921755	2.9896491	1.1815345
H	-1.2938543	3.6023457	-0.1550317
H	-3.0271224	3.9529752	-0.1200447
H	-2.6447781	1.3152614	-2.7794195
H	-3.4375695	2.8763994	-2.4598427
H	-1.6695382	2.7785271	-2.4987697
H	-4.7324825	2.2240090	-0.5052293
H	-4.2700758	0.5583409	-0.9020896
H	-4.0214446	1.1867299	0.7525183
P	3.3612161	-2.9583927	-0.2363543
C	3.3967225	-4.6931040	-0.7335014
H	2.6570550	-5.2611901	-0.1643975
H	3.1861194	-4.7804412	-1.8020665
H	4.3933455	-5.0946273	-0.5312633
C	3.8444750	-2.8635321	1.5086769
H	3.8155781	-1.8196480	1.8333518
H	3.1398580	-3.4435260	2.1097634
H	4.8575962	-3.2528814	1.6439083
C	4.6637068	-2.0946623	-1.1579726
H	4.4657814	-2.1891788	-2.2286574
H	4.6498897	-1.0355149	-0.8858188
H	5.6454491	-2.5155625	-0.9220470
P	-3.6562617	-2.1069444	0.7685006
C	-5.4323791	-1.7989035	0.9488734
H	-5.8485626	-2.4470730	1.7259349
H	-5.5952096	-0.7530789	1.2190827
H	-5.9321079	-2.0043498	-0.0016436
C	-3.4328984	-3.8631298	0.3786474
H	-3.9124718	-4.0872693	-0.5781258
H	-2.3639194	-4.0763105	0.2984876
H	-3.8766263	-4.4853555	1.1613166
C	-2.8512290	-1.7855033	2.3578230
H	-3.2581826	-2.4356054	3.1372174
H	-1.7780545	-1.9655719	2.2505276
H	-3.0106039	-0.7396974	2.6311253

TS4c⁺ TS for **4p** protonation at N-*t*Bu
72
Energy = -1921.485181803

C	3.8945576	-3.1102561	-0.2393367
C	3.2387848	-2.9146607	0.9825989
C	3.6004073	-2.3084993	-1.3442420
H	4.6309389	-3.9035013	-0.3314563
C	2.2868351	-1.9006158	1.1261998
H	3.4708362	-3.5586848	1.8258974
C	2.6519829	-1.2927986	-1.2003617
H	4.0942012	-2.4823519	-2.2963372
C	2.0218140	-1.0773897	0.0387256
H	1.7760437	-1.7517886	2.0725282
C	2.1027414	-0.3466675	-2.1791440
C	0.9919328	0.0507723	-0.0823934
C	1.1315403	0.4520855	-1.5426020
C	2.3923335	-0.1666625	-3.5325589
C	-0.3768635	-0.4458706	0.3455880
N	1.1105219	1.2069784	0.9067233

C	0.4333889	1.4116760	-2.2622085
C	1.7010168	0.8161581	-4.2455618
H	3.1415977	-0.7778271	-4.0278942
C	-0.5667538	0.0126159	1.6927350
C	-1.2644037	-1.2686489	-0.2884545
N	0.3595446	0.8677232	2.1167823
C	0.7257805	1.5977441	-3.6179086
H	-0.3322122	2.0158143	-1.7881160
H	1.9174889	0.9706579	-5.2986345
C	-1.6994461	-0.4744283	2.4142360
C	-2.4236837	-1.7048433	0.4150315
H	-1.0759357	-1.5903280	-1.3093187
C	2.4216490	1.8736685	1.2352560
H	0.1896520	2.3514305	-4.1872479
C	-2.5949216	-1.3021668	1.7714596
H	-1.8604964	-0.1799740	3.4468480
H	0.0824348	2.1757712	0.5142038
C	3.2815541	1.0394106	2.1955757
C	3.1911417	2.1618402	-0.0557034
C	2.0588190	3.2051829	1.9153910
H	-3.4665271	-1.6468591	2.3244521
H	2.6835227	0.7199355	3.0526145
H	3.6985997	0.1601091	1.7023729
H	4.1114223	1.6575569	2.5539258
H	2.5867016	2.7352932	-0.7647608
H	4.0786744	2.7505497	0.1961856
H	3.5237886	1.2413397	-0.5410430
H	2.9771886	3.7207718	2.2106919
H	1.5150185	3.8612750	1.2262729
H	1.4486427	3.0288552	2.8036751
P	-3.6332643	-2.7160087	-0.3453859
C	-5.2467602	-1.8950809	-0.4506050
H	-5.5680420	-1.6083339	0.5546212
H	-5.1519698	-0.9965003	-1.0656634
H	-5.9896047	-2.5678392	-0.8890518
C	-3.9077160	-4.2515900	0.5748866
H	-2.9803074	-4.8293741	0.5941960
H	-4.1986419	-4.0100202	1.6008615
H	-4.7020449	-4.8374954	0.1035685
C	-3.1283197	-3.1520996	-2.0225224
H	-3.0112682	-2.2489700	-2.6264235
H	-2.1858739	-3.7045753	-1.9975780
H	-3.9018852	-3.7821943	-2.4692812
P	-1.3017464	3.0057163	0.6081665
C	-1.2129594	4.5417507	-0.3710141
H	-1.0323586	4.3028921	-1.4229219
H	-2.1440541	5.1100259	-0.2876046
H	-0.3845170	5.1563244	-0.0070713
C	-2.7505558	2.0999389	-0.0247158
H	-3.6417402	2.7337454	0.0144397
H	-2.5686817	1.7916296	-1.0575322
H	-2.9014141	1.2050881	0.5851815
C	-1.7296377	3.5270398	2.2973651
H	-2.6456284	4.1265928	2.2961152
H	-1.8629337	2.6364943	2.9149900
H	-0.9084983	4.1187627	2.7095073

TS4d$^+$ TS for **4c$^+$** C..N ring opening

59

Energy = -1460.260532233

C	2.1415842	-3.8586578	0.8524708
C	1.1849168	-3.3136913	1.7168010
C	2.5872500	-3.1532740	-0.2728296
H	2.5383534	-4.8496010	1.0526342
C	0.6638749	-2.0366906	1.4844225
H	0.8490599	-3.8864655	2.5758072
C	2.0690808	-1.8840447	-0.5123119
H	3.3137954	-3.5999024	-0.9454626
C	1.1206372	-1.3224048	0.3799240
H	-0.0748184	-1.6068828	2.1553552
C	2.2889266	-0.9254727	-1.6034524
C	0.7357088	0.0254502	-0.0864477
C	1.4626095	0.2007869	-1.3711576
C	3.1096690	-0.9701461	-2.7263657
C	-0.6193415	0.5410767	0.1442958
N	1.5048344	1.6212240	0.8715817
C	1.4431892	1.2713947	-2.2606387
C	3.0983032	0.1144735	-3.6131469
H	3.7492511	-1.8267536	-2.9193383
C	-0.7110821	1.8058647	0.8256420
C	-1.7701945	-0.1562552	-0.1728707
N	0.4087683	2.2938891	1.3745629
C	2.2742218	1.2209412	-3.3874529
H	0.7861261	2.1235212	-2.1011837
H	3.7349313	0.0926206	-4.4925893
C	-1.9949757	2.3858502	1.0176399

C	-3.0422351	0.4140318	0.0458248
H	-1.6748090	-1.1391549	-0.6301098
C	2.6801587	1.5471112	1.8247098
H	2.2727949	2.0449922	-4.0942318
C	-3.1267891	1.7030782	0.6298386
H	-2.0707756	3.3628334	1.4832637
H	1.8323903	2.0172941	-0.0204019
C	3.8009107	0.7968367	1.1027037
C	3.1067303	2.9921474	2.1265440
C	2.2362216	0.8273008	3.0954005
H	-4.0958604	2.1663963	0.7871998
H	3.5288245	-0.2446494	0.9186526
H	4.0464223	1.2732905	0.1466323
H	4.6984333	0.8125983	1.7267769
H	2.2964819	3.5357835	2.6164341
H	3.9805978	2.9741573	2.7848612
H	3.3824461	3.5175069	1.2053277
H	3.0610048	0.8306657	3.8133845
H	1.3784847	1.3372404	3.5410149
H	1.9670233	-0.2104191	2.8839955
P	-4.4812606	-0.4751787	-0.4609066
C	-5.9759641	0.3847984	0.0615659
H	-5.9831119	0.4930901	1.1487908
H	-6.0255747	1.3695510	-0.4093164
H	-6.8424925	-0.2047716	-0.2497858
C	-4.4883936	-2.1329423	0.2609305
H	-3.5755607	-2.6610533	-0.0280866
H	-4.5279373	-2.0508117	1.3497464
H	-5.3550063	-2.6927670	-0.1017242
C	-4.5392579	-0.6755589	-2.2580396
H	-4.5871243	0.3101084	-2.7276637
H	-3.6336410	-1.1889821	-2.5927415
H	-5.4152084	-1.2654274	-2.5425881

TS4e$^+$ TS for N..H..C H^+ transfer
59
Energy = -1460.248010630

C	1.3140527	-3.9120615	2.2214901
C	0.4400661	-2.9784286	2.7947474
C	1.9292342	-3.6585196	0.9919510
H	1.5180278	-4.8434986	2.7417655
C	0.1697818	-1.7676559	2.1510487
H	-0.0227898	-3.1948340	3.7533123
C	1.6535654	-2.4587385	0.3351912
H	2.6055574	-4.3900771	0.5582515
C	0.7732039	-1.5110339	0.9194300
H	-0.4915740	-1.0366525	2.6101734
C	2.1194552	-1.9201846	-0.9430295
C	0.7056498	-0.3087137	0.0641389
C	1.5224911	-0.6486426	-1.1376011
C	2.9856430	-2.4374849	-1.9063129
C	-0.5077132	0.5080924	-0.0315317
N	1.7871786	2.0015996	0.2085532
C	1.7961383	0.1059477	-2.2755561
C	3.2556623	-1.6800729	-3.0507976
H	3.4451138	-3.4135539	-1.7753799
C	-0.4468464	1.9346400	-0.1430761
C	-1.7796574	-0.0793414	0.0361015
N	0.6862430	2.6465372	0.2149378
C	2.6673921	-0.4233574	-3.2343353
H	1.3385342	1.0792121	-2.4272565
H	3.9279722	-2.0726496	-3.8081412
C	-1.6259274	2.6843621	-0.3498109
C	-2.9386074	0.6695997	-0.1706219
H	-1.8281857	-1.1545236	0.1891408
C	2.9109349	2.6178440	0.9861449
H	2.8894562	0.1474537	-4.1313877
C	-2.8563049	2.0650947	-0.3966839
H	-1.5410816	3.7622041	-0.4452814
H	1.5353038	0.7116655	0.3529361
C	4.0965597	1.6633582	0.8191171
C	3.2090966	3.9930246	0.3720761
C	2.4970809	2.7473673	2.4597518
H	-3.7491869	2.6551936	-0.5711414
H	3.8651802	0.6766733	1.2352347
H	4.3496643	1.5430650	-0.2384580
H	4.9684214	2.0599297	1.3465500
H	2.3396324	4.6487585	0.4641548
H	4.0541493	4.4445952	0.8997157
H	3.4704298	3.8939463	-0.6860809
H	3.3167028	3.1887508	3.0343243
H	1.6152790	3.3880130	2.5504415
H	2.2684213	1.7642446	2.8853563
P	-4.5103555	-0.1844546	-0.2128795
C	-5.8672070	0.9942564	-0.1132241
H	-5.7775111	1.5865371	0.8006403

H -5.8587543 1.6523162 -0.9853756
H -6.8080400 0.4373258 -0.0959605
C -4.6180265 -1.3243561 1.1806514
H -3.8029404 -2.0508913 1.1320768
H -4.5504099 -0.7608760 2.1142076
H -5.5726043 -1.8559023 1.1373004
C -4.6654481 -1.1298393 -1.7425785
H -4.6142102 -0.4469957 -2.5943771
H -3.8462115 -1.8509663 -1.8065265
H -5.6206778 -1.6621478 -1.7516200

TS4p$^+$ TS for **2p$^+$** deprotonation with PMe_3
72
Energy = -1921.463812747
C 3.1789434 -2.1762821 -2.9532423
C 3.0044023 -2.9116548 -1.7739369
C 2.8377501 -0.8228150 -3.0104587
H 3.5889030 -2.6644474 -3.8328392
C 2.4784266 -2.3052740 -0.6279850
H 3.2831033 -3.9612946 -1.7500213
C 2.3214551 -0.2134094 -1.8643081
H 2.9795761 -0.2596279 -3.9288321
C 2.1479163 -0.9593204 -0.6837189
H 2.3448811 -2.8693635 0.2908841
C 1.9073372 1.1733144 -1.6102497
C 1.5137413 -0.1006175 0.3989217
C 1.4820404 1.2806487 -0.2714435
C 1.8968654 2.2968475 -2.4412061
C 0.0914720 -0.5321705 0.7281047
N 2.1196673 -0.1985625 1.7604950
C 1.0422804 2.4923737 0.2442928
C 1.4671711 3.5189719 -1.9167189
H 2.2227043 2.2294940 -3.4755805
C -0.0100217 -0.4763834 2.1708457
C -0.9276084 -0.9097370 -0.0695585
N 1.1464543 -0.2468114 2.7473541
C 1.0462027 3.6194139 -0.5857194
H 0.7183131 2.5701496 1.2776426
H 1.4615434 4.4022361 -2.5488039
C -1.2805720 -0.7546112 2.7793654
C -2.2638982 -1.1296792 0.5174896
H -0.7946376 -0.9702271 -1.1478916
C 3.4245635 0.3790047 2.1770943
H 0.7190036 4.5785923 -0.1949661
C -2.3362544 -1.0952411 1.9906612
H -1.3877572 -0.7087835 3.8593007
H -2.9263115 0.1591007 0.0990434
C 4.3966999 0.3495356 0.9940358
C 3.2789725 1.8203551 2.7019315
C 3.9645451 -0.5209720 3.3015296
H -3.2938622 -1.3055419 2.4600243
H 4.5494991 -0.6691333 0.6282383
H 4.0455181 0.9734062 0.1666460
H 5.3602131 0.7467481 1.3274604
H 2.4970105 1.8622807 3.4658280
H 4.2231618 2.1472382 3.1504296
H 3.0283340 2.5126916 1.8943186
H 4.9385640 -0.1497962 3.6367972
H 3.2760261 -0.5245348 4.1499119
H 4.0816595 -1.5475730 2.9401027
P -3.2616436 -2.3524050 -0.2797073
C -4.9529242 -2.2692988 0.3492362
H -4.9724777 -2.5411386 1.4072103
H -5.3448009 -1.2559156 0.2282164
H -5.5798866 -2.9699663 -0.2084975
C -2.6822509 -4.0574175 -0.0708698
H -1.6658432 -4.1365510 -0.4669044
H -2.6694883 -4.3005987 0.9950710
H -3.3371247 -4.7559453 -0.5998248
C -3.3107629 -2.0191844 -2.0560219
H -3.6979179 -1.0129635 -2.2357425
H -2.3092332 -2.1047684 -2.4843866
H -3.9651321 -2.7470807 -2.5426131
P -3.1815682 1.7966095 -0.2942450
C -2.1543123 2.1997663 -1.7447329
H -2.4820655 1.6003860 -2.5992484
H -2.2373691 3.2607256 -1.9997394
H -1.1100760 1.9644154 -1.5239098
C -4.8826931 2.2887902 -0.7351757
H -4.9227925 3.3506920 -0.9985592
H -5.2252444 1.6971050 -1.5891400
H -5.5482323 2.1038126 0.1125644
C -2.6605887 2.9241224 1.0387136
H -2.6884647 3.9657863 0.7027973
H -3.3281793 2.7997006 1.8954377

H	-1.6450166	2.6655648	1.3451496

TS4⁺ TS for final aryl rotation of **4e⁺**

59

Energy = -1460.257547325

C	1.7478180	4.3737650	-0.7598162
C	2.4674116	3.7446529	0.2602711
C	0.4385799	3.9821350	-1.0487135
H	2.1969993	5.1985098	-1.3054047
C	1.9072753	2.6745055	0.9704523
H	3.4610944	4.0991042	0.5182763
C	-0.1251837	2.9264856	-0.3290974
H	-0.1387072	4.5132701	-1.8002574
C	0.6277120	2.2350155	0.6432628
H	2.4646889	2.2274837	1.7904480
C	-1.5100937	2.4488231	-0.2693244
C	-0.1978886	1.0713763	1.2051996
C	-1.5937988	1.4445457	0.7174514
C	-2.6637956	2.9518280	-0.8739663
C	0.3579089	-0.2785143	0.6770368
N	-2.3928906	-2.0859343	-0.4605020
C	-2.8316531	1.0369481	1.1942255
C	-3.9062102	2.4925649	-0.4308520
H	-2.6036465	3.7200566	-1.6397750
C	-0.3973931	-1.4089800	0.2854166
C	1.7435438	-0.3893433	0.5374699
N	-1.8085779	-1.5232271	0.4900900
C	-3.9899244	1.5702185	0.6167475
H	-2.8999450	0.3234540	2.0067388
H	-4.8159340	2.8858018	-0.8753102
C	0.2388395	-2.5585162	-0.2181378
C	2.3783208	-1.5294688	0.0296319
H	2.3511876	0.4647603	0.8068108
C	-3.8189543	-2.4398536	-0.2395430
H	-4.9644922	1.2691326	0.9899154
C	1.6136588	-2.6345048	-0.3591276
H	-0.3882056	-3.4002342	-0.4899922
H	-0.1597308	1.0345775	2.3014646
C	-3.8891996	-3.9325484	-0.6188421
C	-4.3239191	-2.2225088	1.1857175
C	-4.6084323	-1.6051691	-1.2649678
H	2.0683817	-3.5338746	-0.7605944
H	-3.4879540	-4.0938484	-1.6233894
H	-3.3187389	-4.5401692	0.0915060
H	-4.9329034	-4.2610594	-0.5967743
H	-4.3453497	-1.1637719	1.4465150
H	-5.3409119	-2.6184843	1.2687356
H	-3.6873872	-2.7431686	1.9078035
H	-5.6623886	-1.8983176	-1.2348558
H	-4.5302682	-0.5393848	-1.0340754
H	-4.2236332	-1.7767638	-2.2745781
P	4.1575849	-1.4887264	-0.1403154
C	4.6216556	-0.0770264	-1.1649747
H	4.1843556	-0.1956773	-2.1592898
H	4.2430445	0.8436121	-0.7119171
H	5.7109355	-0.0221651	-1.2423553
C	4.7496663	-3.0035960	-0.9092193
H	4.4998691	-3.8628903	-0.2819625
H	4.2977254	-3.1199222	-1.8973952
H	5.8360308	-2.9383911	-1.0143468
C	4.9313358	-1.2993143	1.4780500
H	4.5613599	-0.3867045	1.9532620
H	4.6775176	-2.1592934	2.1025298
H	6.0164637	-1.2322464	1.3602910

References

1 *TURBOMOLE V7.3*, **2018**, a development of University of Karlsruhe and Forschungszentrum Karlsruhe GmbH, 1989-2007, TURBOMOLE GmbH, since 2007; available from http://www.turbomole.com.

2 J. Tao, J. P. Perdew, V. N. Staroverov and G. E. Scuseria, *Physical Review Letters*, 2003, **91**, 146401.

3 S. Grimme, J. Antony, S. Ehrlich and H. Krieg, *The Journal of Chemical Physics*, 2010, **132**, 154104-154119.

4 S. Grimme, S. Ehrlich and L. Goerigk, *Journal of Computational Chemistry*, 2011, **32**, 1456-1465.

5 F. Weigend, M. Häser, H. Patzelt and R. Ahlrichs, *Chemical Physics Letters*, 1998, **294**, 143-152.
6 F. Weigend and R. Ahlrichs, *Physical Chemistry Chemical Physics*, 2005, **7**, 3297-3305.
7 A. Klamt and G. Schüürmann, *Journal of the Chemical Society, Perkin Transactions 2*, 1993, 799-805.
8 K. Eichkorn, F. Weigend, O. Treutler and R. Ahlrichs, *Theoretical Chemistry Accounts*, 1997, **97**, 119-124.
9 F. Weigend, *Physical Chemistry Chemical Physics*, 2006, **8**, 1057-1065.
10 P. Deglmann, K. May, F. Furche and R. Ahlrichs, *Chemical Physics Letters*, 2004, **384**, 103-107.
11 S. Grimme, *Chemistry - A European Journal*, 2012, **18**, 9955-9964.
12 F. Eckert and A. Klamt, *AIChE Journal*, 2002, **48**, 369-385.
13 F. Eckert and A. Klamt, COSMOtherm, Version C3.0, Release 16.01; COSMOlogic GmbH & Co. KG, Leverkusen, Germany 2015.
14 Y. Zhao and D. G. Truhlar, *The Journal of Physical Chemistry A*, 2005, **109**, 5656-5667.
15 F. Weigend, F. Furche and R. Ahlrichs, *The Journal of Chemical Physics*, 2003, **119**, 12753-12762.
